# *ModuleFinder *and *CoReg*: alternative tools for linking gene expression modules with promoter sequences motifs to uncover gene regulation mechanisms in plants

**DOI:** 10.1186/1746-4811-2-8

**Published:** 2006-04-11

**Authors:** Kathryn E Holt, A Harvey Millar, James Whelan

**Affiliations:** 1 ARC Centre of Excellence in Plant Energy Biology, CMS Building M310 University of Western Australia, 35 Stirling Highway, Crawley 6009, Western Australia, Australia

## Abstract

**Background:**

Uncovering the key sequence elements in gene promoters that regulate the expression of plant genomes is a huge task that will require a series of complementary methods for prediction, substantial innovations in experimental validation and a much greater understanding of the role of combinatorial control in the regulation of plant gene expression.

**Results:**

To add to this larger process and to provide alternatives to existing prediction methods, we have developed several tools in the statistical package R. *ModuleFinder *identifies sets of genes and treatments that we have found to form valuable sets for analysis of the mechanisms underlying gene co-expression. *CoReg *then links the hierarchical clustering of these co-expressed sets with frequency tables of promoter elements. These promoter elements can be drawn from known elements or all possible combinations of nucleotides in an element of various lengths. These sets of promoter elements represent putative *cis*-acting regulatory elements common to sets of co-expressed genes and can be prioritised for experimental testing. We have used these new tools to analyze the response of transcripts for nuclear genes encoding mitochondrial proteins in *Arabidopsis *to a range of chemical stresses. *ModuleFinder *provided a subset of co-expressed gene modules that are more logically related to biological functions than did subsets derived from traditional hierarchical clustering techniques. Importantly *ModuleFinder *linked responses in transcripts for electron transport chain components, carbon metabolism enzymes and solute transporter proteins. *CoReg *identified several promoter motifs that helped to explain the patterns of expression observed.

**Conclusion:**

*ModuleFinder *identifies sets of genes and treatments that form useful sets for analysis of the mechanisms behind co-expression. *CoReg *links the clustering tree of expression-based relationships in these sets with frequency tables of promoter elements. These sets of promoter elements represent putative *cis*-acting regulatory elements for sets of genes, and can then be tested experimentally. We consider these tools, both built on an open source software product to provide valuable, alternative tools for the prioritisation of promoter elements for experimental analysis.

## Background

The regulation of gene expression is one of the most intensively studied areas of biology. The regulation of transcription, the first committed step in gene expression, is achieved via the interaction of transcription factors with *cis *acting regulatory elements (CAREs) [1]. A complete understanding of the interaction between transcription factors and regulatory sequences will ultimately lead to a picture of the regulatory networks operating in a biological system. Genome wide studies on the expression of transcription factors are currently underway in attempts to gain data that can be used to understand the complex nature of gene regulation that exists to coordinate cellular functions [2-4]. The structure of such regulatory networks (multi-component regulatory factors that have overlapping but also discrete activities) for a plant can begin to be hypothesized using the ~1,500 transcription factors in *Arabidopsis *in a combinatorial manner to achieve regulation of the 28,000 or more genes [5-7].

The completion of the *Arabidopsis *nuclear genome sequence means that the analysis of plant gene expression has changed from probing the expression of a single or few genes at a time to simultaneous analysis of the expression of virtually every gene [8]. This change in the amount of data available represents a considerable challenge for biologists to extract knowledge from these data and use it in a productive manner to investigate the mechanisms underlying gene regulation, i.e. the further dissection of a complex network of combinatorial control.

The analysis of *Arabidopsis *microarray expression data sets can be carried out from single gene analysis to whole genome approaches. At a single gene level many researchers can simply look up how their gene or genes of interest are changing under a large number of conditions. This approach has been facilitated by the use of tools such as Genevestigator, which enables complex array data to be easily interrogated for a gene of interest [9]. At a wider genome level hierarchical clustering has been applied to complete genome transcriptomic data during growth and development [10-13], following various biotic and abiotic treatments [14-16] and after alterations in transcript abundances due to changes in nutrient availability [17]. Development of analysis packages such as MAPMAN has allowed plant biologists to visualize transcriptomic data on metabolic pathways that should lead to a greater understanding and use of transcriptomic data [18].

Even though large-scale analysis like those above can and has identified novel associations of biological significance, the clustering methods used can also tend to split or miss relationships in such data. The transcripts from a group of genes may respond to a number of parameters in a similar manner, but in additional treatments their response may differ. In a hierarchical cluster analysis of all these treatments the relationship between these genes will often be masked and they will be separated to different parts of the clustering tree. This loss of association is further compounded by the fact that clustering of gene expression data is often carried out with the intent to identify co-expressed genes and then these data used to elucidate the regulation of these genes, i.e. to identify CAREs and the transcription factors that bind them. As transcription factor binding sites are small in size (6 to 10 bp [1]) compared to the large number of DNA bases in promoter regions, there is a significant challenge in identifying these regions of important sequence. Direct experimental confirmation requires considerable effort, so computational efforts to identify the most likely putative CAREs are essential. The identification of similar CAREs in co-expressed genes thus becomes crucial as it will determine the quality of input for such analysis.

An alternative approach to hierarchical clustering to analyse array expression data is to define associations based on similarities in transcript abundance in a subset of treatments. Such two way clustering or biclustering uses iterative approaches to define relationships between subsets of genes and subset of treatments. This approach has been most widely used in the analysis of transcript datasets from cancer samples [19-25]. Various approaches such as the progressive iterative signature algorithm (PISA) [26], gene expression mining server (GEMS) [25], coupled two-ways clustering (CTWC) [27] and X-Motifs [28] use this principle to search for relationships that go largely undetected using hierarchical clustering.

We have taken a biclustering approach to identify co-expressed genes and the prediction of the CAREs. Firstly we have simplified the number of genes analyzed by using only a subset, in this example those that encode proteins located in mitochondria [29,30]. Secondly we have identified genes that are co-expressed in response to subsets of treatments using a novel approach via a tool we have developed and named *ModuleFinder*. The pattern of co-expressed genes produced in *ModuleFinder *can be exported to visualize functional groups in tools such as MAPMAN. To predict CAREs we have used the hierarchical clustering produced in *ModuleFinder *and the assumption that the resulting hierarchical tree structure of the expression data is a reflection of patterns of CAREs in promoter regions. Thus the hierarchical relationships identified based on the expression data can be used to identify these promoter elements. We have developed a tool named *CoReg *to undertake this CARE prediction.

## Results and discussion

### Existing approaches are not well suited to identifying shared responses among numerous non-linear-related treatments

**Figure 1:**
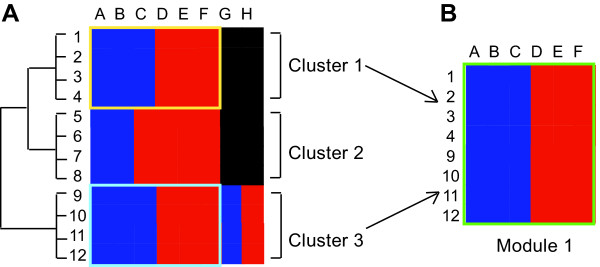
*Shared gene expression responses can be split in simple cluster analysis*. A) Classical cluster analysis groups together genes whose expression patterns are similar across all available experiments. This cluster analysis of genes 1 to 12 in treatments A to H splits the genes into three separate clusters. B) Clusters 1 and 3 (genes 1–4 and 9–12) are co-ordinately expressed in response to treatments A-F.

Cluster analysis is a useful technique for identifying genes whose expression patterns across a given set of treatments are similar. For example, such analysis will cluster together all those genes whose expression is up-regulated in response to treatments A, B and C, down regulated in response to treatments D, E and F, and unaffected by treatments G and H (Figure 1, cluster 1). However since expression data from all treatments is used in the analysis, this cluster will not include genes that are up-regulated in response to A, B, C and G, and down-regulated in response to D, E, F and H (Figure 1, cluster 3). These will be grouped together into a separate cluster since their expression patterns differ under treatments G and H (Figure 1, cluster 3). The similarity between the clusters in response to treatments A to F is masked in the analysis and the cluster tree. Yet from a biological point of view, the fact that both clusters display co-ordinated expression in response to treatments A to F is very interesting. It may indicate that they are co-regulated by a factor that is induced or activated under treatments A-C and repressed or inactivated under treatments D-F. Thus it would be informative to identify the genes of cluster 1 and 3, and the treatments A-F, as a co-ordinated gene expression module. Such a module contains more member genes, and in the analysis of this larger set it can be argued it is more likely that a biological significant mechanism might become apparent than in analysis of the two separate smaller groups produced by classical cluster analysis.

We have thus developed *ModuleFinder *in *R *with the aim of identifying gene expression modules in a way that facilitates the subsequent interpretation of results. The method was designed to allow easy visualization not only of the expression patterns of discrete modules, but also of the relationships between the modules. The aim of *ModuleFinder *is to identify gene expression responses that are shared among subsets of treatments and genes; the approach is to first identify gene clusters that are co-expressed in a small subset (often a pair) of treatments, then look for other treatments in which these gene clusters are expressed in a similar co-ordinated manner. This approach ignores the differences in treatment effects and focuses on the shared effects on gene expression, which are expected to be related to the activation of common gene regulatory pathways.

### The ModuleFinder algorithm

*ModuleFinder *takes as its input a matrix of expression data from a set of experiments, for example the set of average log expression ratios for genes from a range of experimental treatments compared to a control. It also requires a matrix of p-values associated with each data point, providing an assessment of how likely it would be to observe the gene expression values if there was really no change in experimental compared to control conditions. *P*-values may be calculated from the original expression measures via an appropriate statistical method, e.g. *t*-tests.

**Figure 2:**
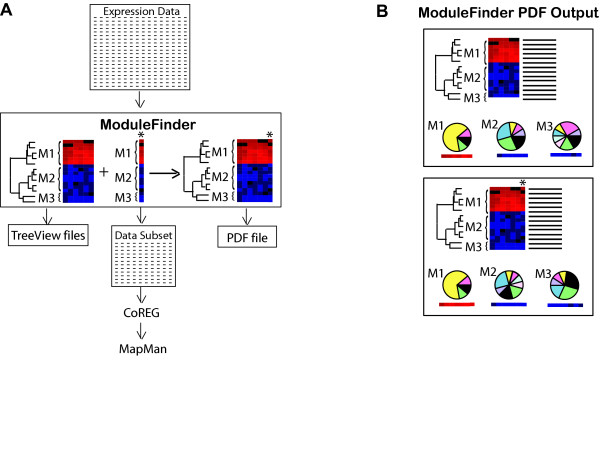
*An overview of the operation of ModuleFinder*. A) Flow diagram of *ModuleFinder*. Sets of expression data are taken as input, subsets of genes and experiments are hierarchically clustered and then experiments with similar expression profiles are added consecutively (indicated with an asterisk), expression data and TreeView cluster files are saved after each addition and the entire run is documented in a PDF file. B) The PDF output file includes a heatmap, clustering tree and functional breakdown of the modules at each stage of the run.

The algorithm begins with a subset of experiments and extracts the genes whose expression levels differ from control conditions in those experiments, according to the p-values provided. *ModuleFinder *then clusters the genes hierarchically and splits them into co-expressed modules based on the resulting clustering tree. Next, the algorithm searches for another experiment (outside the initial subset) that fits the expression patterns of these modules. The new experiment is added to the module and the genes are re-clustered. Experiments are added one by one in an iterative procedure of searching for matching experiments and re-clustering the genes, until no more experiments can be found that fit the module expression patterns. The resulting subsets of genes and experiments are referred to as gene expression modules, as they define not only gene clusters but also subsets of genes whose expression is co-ordinated in a specific subset of experiments. A general scheme of the program is illustrated in Figure 2A.

The *ModuleFinder *algorithm can be run in either a supervised or unsupervised fashion. In an unsupervised run, the algorithm first searches for pairs of experiments in which gene expression was similar (i.e. highly correlated), then builds gene expression modules based on these correlated pairs. On the other hand, the user can identify a particular subset of experiments they are interested in, and run the algorithm in a supervised manner by specifying the names of the experiments to provide an initial subset. This initial set will then be added to by iterative additions of related experiments.

The main output of *ModuleFinder *is a PDF file containing clustering trees and expression heat maps of the modules produced after the addition of each new experiment. It also includes pie charts displaying the breakdown of each module according to the functional categories of its member genes (Figure 2B). In addition, cluster files are written at each stage for easy viewing of clusters, heat maps and gene annotations in tree viewing programs compatible with TreeView and its java versions, which can run on any platform [31]. Excel-compatible, comma-separated files containing the expression data for the subsets of genes and experiments are also saved at each stage (Figure 2A).

### Using ModuleFinder to identify modules within the expression of a set of nuclear genes encoding mitochondrial proteins (NGMP)

**Figure 3:**
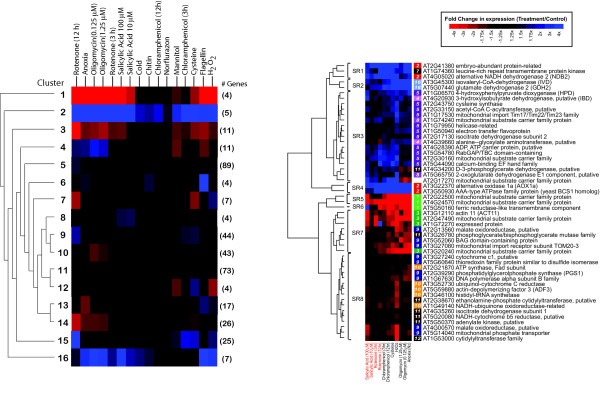
*Traditional hierarchical cluster versus ModuleFinder analysis of 374 Nuclear Genes encoding Mitochondrial Proteins (NGMPs)*. A) The 374 NGMPs were clustered into 16 clusters in response to 16 treatments. This analysis split genes that have related function in a subset of treatments. B) Using the same starting set of genes and treatments, but seeding the *ModuleFinder *with salicylic acid and rotenone treatments (indicated in red) a different grouping of genes is produced. This output contained only 51 genes, divided into 8 modules; this used a p-value cut-off of 0.1, Euclidean distance, complete linkage and a between-to-within-groups variance ratio > 4. The number to the left of the heat map indicates the cluster number that these genes belonged to in the analysis carried out in A above.

Traditional hierarchical clustering was compared to *ModuleFinder *in analysing the expression of 374 *Arabidopsis *NGMPs in a set of microarray experiments where *Arabidopsis *suspension cell cultures were subjected to 16 different chemical stresses [32]. The clustering trees and expression heat maps using a standard clustering method (hierarchical clustering using a Euclidean distance measure and the McQuitty method of linkage) are shown in Figure 3A[33]. The full clustering tree and heat map showing all individual gene responses are shown in Supplementary Figure 1A. A set of 16 clusters can be defined from this analysis ranging in size from 4 to 89 genes. For the *ModuleFinder *analysis, four experiments comprising salicylic acid at 10 and 100 μM and rotenone after 3 and 12 hours were selected as an initial experiment subset. These treatments had been shown to induce similar responses in the expression of the alternative respiratory pathway components of plant mitochondria [32]. Using a p-value cut-off of 0.1, a total of 51 (14%) of the genes could be selected in these experiments and clustered into eight modules. A further seven treatments were added by *ModuleFinder *and are shown in Figure 3B. A number of genes that were separated into several different clusters using hierarchical clustering (Figure 3A) were placed into the same modules or closely related modules by *ModuleFinder *(Figure 3B). Therefore the similarity in biological response to these treatments becomes readily apparent, with genes that are uniformly induced and genes that are uniformly repressed by the treatments are identified. Analysis of the same data set using the coupled two-way clustering (CTWC) algorithm yielded an intermediate set of results to the traditional hierarchical clustering and analysis by *ModuleFinder *(data not shown). Of the 24 genes whose transcript abundance was increased as shown in Figure 3B, 7 and 4 were placed in two close clusters, indicating that the CTWC algorithm and ModuleFinder were placing gene together that were split in the traditional hierarchical clustering [27]. However using several iterative clustering steps with treatments (sample clusters in CTWC terminology) the apparent relationships between treatments defined by ModuleFinder were not evident in the CTWC results. This may be due to the fact that the initial clustering of treatments and genes is based on the entire data set, so the problems illustrated in Figure 1 remain. Furthermore CTWC works by clustering genes into subsets, then clustering samples into subsets. Each gene subset-sample subset pair is then considered as a sub-matrix and genes and samples are re-clustered within that sub-matrix [21,27]. The result is a collection of subsets of genes and samples (gene expression modules), which theoretically should display co-ordinated expression patterns. However, this fragmentation of the data into small discrete modules makes it difficult to interpret the CTWC results and particularly difficult to see overall trends in the expression patterns displayed by ModuleFinder.

### Visualization of ModuleFinder sets in MAPMAN

**Figure 4:**
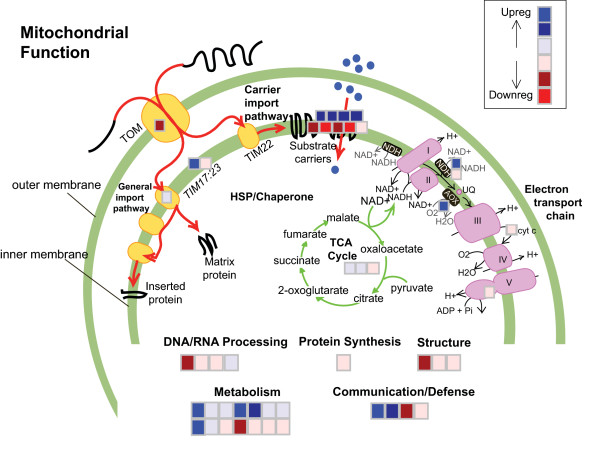
*Visualization of the expression of Nuclear Genes encoding Mitochondrial Proteins using MAPMAN*. Pictorial representation of mitochondrial functions of the changes in gene expression from *ModuleFinder *as carried out in Figure 3B. The mitochondrial outer membrane contains the Translocase of the Outer Membrane (TOM) complex and the Translocase of the Inner Membrane 17:23 and 22 (TIM17:23 and TIM22) which are responsible for the import of all mitochondrial proteins synthesised in the cytosol [60]. The substrate carriers refer to the family of mitochondrial carrier proteins characterised by six transmembrane regions and responsible for the import and export of various metabolites in and out of mitochondria [61]. The mitochondrial electron transport chain consisting of four multi-subunit electron transport complexes and the ATP synthase complex are labelled I to V. The alternative electron transport chain components, alternative NAD(P)H dehydrogenases (NDH) and alternative oxidase (Aox) are shown. The TCA cycle and a range of other functions of mitochondria are listed [62]. The boxes represent the average gene expression for the 47 genes in Fig 3B divided into the functional annotation for these genes in Mapman.

The output from *ModuleFinder *can be visualized using MAPMAN to display functional categories. MAPMAN is a Java program that allows users to annotate images with data from a text file [18]. Once the appropriate images and annotation files are loaded into the program, users can load in a file containing a list of gene identifiers with values assigned to each (e.g. an expression value from a particular experiment), and the genes are mapped onto the pathway image, coloured according to the value (e.g. expression level) in the loaded file. This helps users to recognize if a number of genes in a pathway were induced or repressed in an experiment. A number of mappings and annotated images come with the standard MAPMAN download, but users can also add their own. To facilitate functional interpretation of the results presented here, which focus specifically on this set of mitochondrial-targeted genes, a new MAPMAN annotation was developed to aid visualization of changes in expression of components of the various pathways of plant mitochondria (Figure 4). The mapping includes the classical and alternative mitochondrial electron transport chains in some detail, as well as components of the mitochondrial import machinery, substrate transporters and TCA cycle enzymes, and can be used to visualize data from any source in which genes are labelled with AGI locus identifiers. The necessary files are available as part of the *ModuleFinder *package. The annotated image highlighted that the highest up-regulated group contained two genes that together form an alternative respiratory pathway: alternative oxidase 1a (*Aox1a*) and an external class alternative NADH dehydrogenase (*NDB2*). The next most up-regulated group contained several mitochondrial substrate carriers and genes involved in metabolism. The annotated image also suggested some down-regulation of genes involved in import of proteins and substrates into the mitochondria, as well as functions associated with expression of the mitochondrial genome (DNA/RNA processing, transcription and protein synthesis).

### Building a framework for understanding the biological implications of the gene regulation observed

Combining the MAPMAN overview with a more detailed analysis using the wider literature provided an even deeper view of the biological response to rotenone and salicylic acid, showing this process was helpful for a biologists' interpretation of the dataset. Rotenone is an inhibitor of complex I function, thus preventing matrix-located NADH from the TCA cycle entering the classical respiratory chain. Salicylic acid can have a similar effect, as it appears that along with its defence signalling functions this compound can inhibit the respiratory chain in plants [34]. This effect appears to be through inhibition of the dehydrogenases of the mitochondrial electron transport chain [35]. Induction of the Aox and NADH dehydrogenase are the clearest direct response to this targeted inhibition of mitochondrial function evident from both types of cluster analysis (Figure 3). Using the classical cluster analysis it appeared that the up-regulation of gene expression in response to respiratory poisons was split, in clusters 2, 4, 5, 15 and 16, and down-regulation split into 1, 3, 10 and 14 (Fig 3A, Supplementary Figure 1A). Many of the genes in cluster 9 and 15 are involved in protein synthesis or mitochondrial biogenesis (Supplementary Figure 1B). We have previously reported that changes in protein import into mitochondria and a general up-regulation of genes encoding components involved in mitochondrial biogenesis occur as a result of chemical and environmental stresses [36,37].

Using *ModuleFinder *a larger picture of the effects of these chemical stresses on the expression of mitochondrial components becomes evident. In the defined subset of co-expressed genes the induction of the alternative transport chain components is coupled to the induction of transcripts encoding for eight different substrate dehydrogenases, providing new avenues for NADH generation, or in the case of the electron transfer flavoprotein (At1g50940), provision of electrons to ubiquinone. Significantly, the new carbon substrates for these NADH generating pathways, while including the organic acids of the TCA cycle, are likely to be generated by catabolism of amino acids. Enzymes involved in valine, isoleucine, cysteine, tyrosine, alanine and glutamate catabolism are induced. Concomitant with this change in substrate for energy generation is the upregulation of transcripts for 4 mitochondrial carrier proteins, most of unknown function. Down-regulation is observed for components of the classical electron transport chain complexes I and III, a separate set of five mitochondrial substrate carriers (most of unknown function) and lipid biosynthesis pathways for phosphotidylglyerol and phosphotidylethanolamine. Interestingly, both genes for NAD-malic enzyme (At4g00570, At2g13560) are down-regulated. This protein normally bridges the TCA cycle to allow the anaplerotic removal of organic acids for functions elsewhere in the cell. Together the insights from this analysis suggests that these simple chemical inhibitors appear to initiate the signals for a complicated re-organisation of mitochondrial function within the plant cell that can now been investigated independently.

### Searching for common regulatory elements in the promoters of co-expressed genes

Genes whose transcription is co-ordinately regulated may exhibit co-ordinated expression patterns. Thus co-expression of a group of genes may be indicative of co-regulation at the transcriptional level [38]. To determine whether this is the case for a given cluster of co-expressed genes, such as those shown above, the promoter regions of the genes need to be analyzed. Transcription factors (TFs) bind to specific DNA sequences, which are usually only 6 to 10 base pairs long [1]. These short sequences are often referred to as promoter motifs or sequence elements. Transcriptional regulation in eukaryotes most often occurs through the combinatorial action of multiple TFs [1,39,40]. For example, the induction and repression of *Arabidopsis *genes in response to red and blue light or abscisic acid (ABA) is dependent on combinations of multiple light-responsive or ABA-responsive promoter elements [41,42]. It is therefore expected that the promoter regions of co-expressed genes may share numerous TF binding sites, including some that are also present in the promoter regions of genes whose expression patterns are quite different. A limitation of this type of approach is that genes may be regulated by the same transcription factor(s) but display different pattern(s) of transcript abundance due to the fact that post-transcriptional processes that affect their transcript stability may differ.

### Aims of promoter analysis

Modules of co-expressed genes identified using *ModuleFinder *(Figure 3B), or groups of genes identified as co-expressed by other methods, provide an opportunity to discover potential regulatory sequence elements that may be responsible for the observed co-expression. The aims of such an analysis could be:

(a) to identify promoter sequence elements (possible TF binding sites) that are common to genes within a module,

(b) to identify promoter sequence elements that are common to up-regulated genes or downregulated genes but not both,

(c) to identify combinations of promoter sequence elements that are common within a module but not shared by other modules, and

(d) to use the identified promoter motifs to construct testable hypothetical models of gene regulation that explain observed expression patterns in terms of patterns of regulatory elements.

Various motif recognition tools are available which can identify promoter sequence elements that are common among a group of genes, many of them available as web-based programs [43,44]. However this becomes difficult when there are large numbers of large groups to be analyzed, as the processing times for these programs generally increase exponentially with the number of sequences taken as input data. Assuming such programs could be employed, it would be possible to build up a model of the regulatory network responsible for observed patterns of gene expression by applying these tools repeatedly to gene clusters defined by cluster analysis or gene modules defined by *ModuleFinder *analysis. Unfortunately such a process would be time consuming and error-prone. The identification of motifs conserved in multiple sequences is a complicated computing task and can consume significant processing time. To achieve the aims outlined above, this task must be repeated for each module and subset of modules and each potential motif would then have to be searched against all the other promoter sequences. Keeping track of module memberships and relationships, promoter sequences and motifs is a complicated task in itself. If this involves using the current web-based tools it requires considerable uploading, copying and pasting of gene lists and sequences, which can also introduce errors. A more attractive alternative is to try to identify sequence elements whose presence in gene promoter regions can be correlated with observed gene expression levels [45]. This approach was implemented using clustering-based methods in a novel tool called *CoReg *(Co-Regulation of Co-Expressed Genes) to undertake promoter analysis by deducing models of gene co-regulation to explain observed patterns of gene co-expression.

### The CoReg algorithm

**Figure 5:**
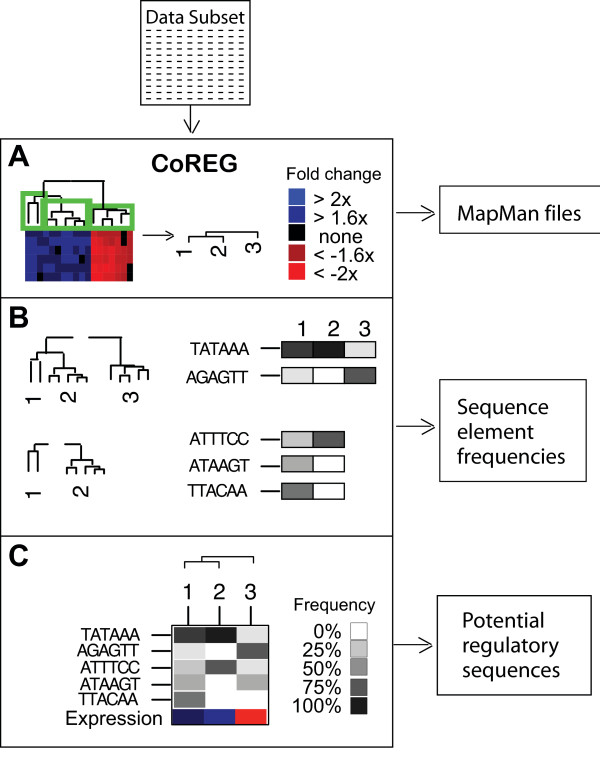
*An overview of the operation of CoReg*. The expression data output from *ModuleFinder *is taken as input (A) and the user defines the number of groups for analysis by *CoReg*. Files are also saved for visualization of expression data by MAPMAN. B) Sequence elements are identified that are unevenly distributed between the promoters of groups defined in A. The frequencies of these elements in each group are recorded. C) Various combinations of elements can be selected and saved.

**Figure 6:**
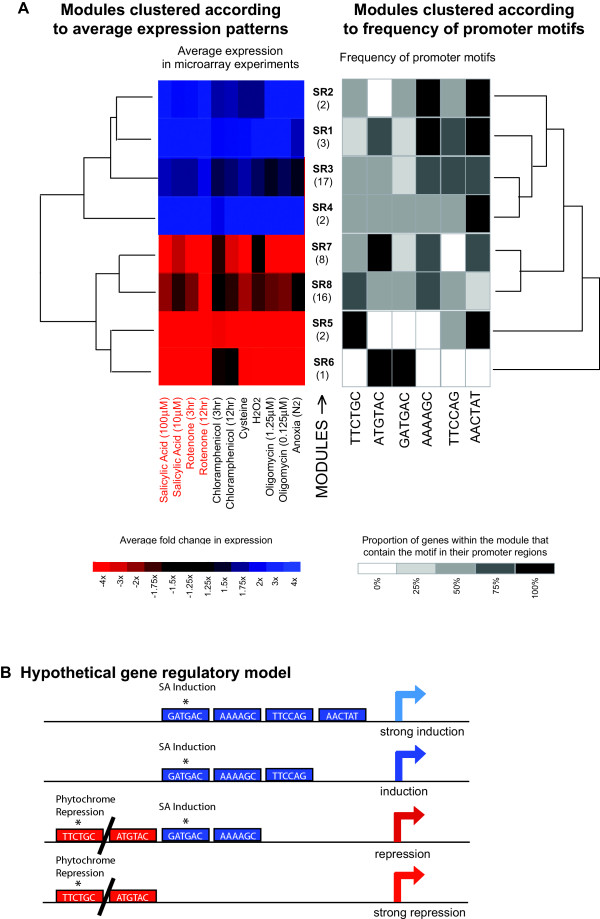
*CoReg analysis of the salicylic acid/rotenone module group from Nuclear Genes encoding Mitochondrial Proteins*. The eight modules produced by *ModuleFinder *(Figure 3) were analyzed by *CoReg*. A) From the variety of elements detected in the upstream regions, five were selected which produced a tree structure that closely resembled the tree structure produced from the expression data. B) Hypothetical models of elements governing gene expression are shown based on these elements, which can be tested by experimental analysis. The sequences previously identified as regulatory elements in plants are indicated.

*CoReg *aims to identify regulatory elements in the promoter regions of a set of co-expressed genes, which explain the observed expression patterns of those genes. It is based on the assumption that there is a relationship between the degree of similarity in gene expression and the degree of similarity in the combination of transcription factors binding within gene promoters. *CoReg *takes as its starting point a hierarchical clustering of a set of genes according to their expression in a set of experiments, for example the output of *ModuleFinder*. The user is then asked to break the hierarchical tree down into discrete groups of genes (Figure 5A). The assignment of genes into discrete groups can be recorded in a text file, which can be loaded into MAPMAN to aid interpretation of the functional significance of these groups (as indicated above). The *CoReg *algorithm then navigates down the tree, stopping at the first point at which the tree splits into two branches, and searches for sequence elements whose frequency of occurrence in promoter sequences varies greatly between the two groups of genes defined by the branches. For example, depending on the parameters set it will identify any sequence elements that are present in the promoters of all the genes in one group but none in the other group, or in promoters of >80% of the genes in one group but <20% of the second group. The two branches resulting from the first split are then each broken down into two groups and sequence elements identified in each, then the process is repeated until the specified groups are reached. This process is illustrated in Figure 5B. *CoReg *can also search for sequence elements that are 'characteristic' of each group, in that their frequency is particularly high or low in that group. A separate frequency tolerance value may be set for this purpose. The user provides CoReg with a list of sequence elements to search for, these may be known elements from databases such as PlantCare [46], Place [47], AGRIS [48] or Athamap [49], or list of all possible combinations of nucleotides ranging from 3 to X nucleotides, where X would be an upper limit to the size of a transcription factor binding site. Degenerate binding sites can also be included where N can be any nucleotide. All these potential motifs can be included in a single file and the user can select the elements that match the expression profile (Figure 5 and 6). The example provided contains a built-in list of all hexamers, that is all possible sequences of the bases A, C, G, T of length six.

The frequency of each of the identified sequence elements in each of the gene groups is then calculated, and displayed as a greyscale heatmap (dubbed frequency map) in which black corresponds to a frequency of 80–100%, shades of grey intermediate values and white 0–20% (Figure 5B–C). The gene groups are then clustered according to the frequencies of the identified elements in the promoter regions of their member genes (Figure 5C). At this point, the algorithm has done the bulk of its work, and it is up to the user to drive the selection of a final subset of the identified sequence elements. The user can choose to try random subsets of sequence elements, chosen by CoReg using random sampling methods, or can select their own subsets to try. For each subset of sequence elements, the image window is updated to display a frequency map for the subset of elements, and a hierarchical tree showing the gene groups clustered according to these frequencies. The aim here is to try to find a subset of sequence elements such that, when the gene groups are clustered according to the frequencies of the elements in the promoter regions of their member genes, the resulting tree has the same structure as the expression-based hierarchical clustering tree. It can then be proposed that the selected sequence elements capture the structure of the observed gene expression patterns, and it can be hypothesised that the sequences correspond to regulatory elements that are responsible for these patterns of gene expression. Experiments may then be designed to test these hypotheses in the laboratory.

While the criteria of tree matching provides a good visual cue to spot relationships between gene expression and the occurrence of sequence elements, it is up to the user to decide when they have found a set of sequence elements that might explain the observed expression patterns. The frequency maps themselves provide visual cues, helping the user to spot other patterns that may be useful. Thus, rather than providing the user with a definitive list of promoter elements that might be regulatory, *CoReg *is a tool for the user-driven exploration of patterns relating gene co-expression and co-regulation. *CoReg *scans for the specific elements present and thus will not identify degenerate elements.

### Using CoReg to identify putative sequence elements in subsets of co-expressed nuclear genes encoding mitochondrial proteins

We have used *CoReg *to analyze the gene expression modules identified by *ModuleFinder *analysis as described in the example above (Figure 3B). To do this, the file containing expression data for the 51 genes in the module, created during the *ModuleFinder *run, was loaded into CoReg along with the promoter sequences for these genes. The built-in list of hexamers was taken as the list of sequence elements for the search. The hierarchical clustering tree was broken down into eight groups – four up-regulated (Group 1 to 4) and four down-regulated (Group 5 to 8) in response to the various treatments. The resulting tree is shown in Figure 6A. The maximum frequency tolerance was set to 0.35 and the characteristic frequency tolerance to 0.1, meaning that at each split in the tree, any sequence element present in promoters of >65% of the genes in one group but <35% of the other group would be identified as interesting, as would any sequence elements with a frequency of >90% in one group but <10% in all other groups. A subset of 6 of these elements was identified which resulted in a clustering of the gene groups that was quite similar to the expression-based clustering (Figure 6A). This suggests that although the element-based tree did not precisely match the expression-based tree, the uniqueness of expression pattern is reflected in the uniqueness of its promoter composition, relative to the other groups. Therefore the high frequency of the elements TTCTGC and ATGTAC correlate with the down regulation of modules SR 5 to 8, while the high frequency or AAAAGC, TTCCAG and AACTAT correlate with the up regulation of modules SR 1 to 4. GATGAC is present in all except the most highly downregulated module SR5.

These correlation patterns can then be used to model gene regulatory networks that can be prioritised for experimental testing (Figure 6B). Of the six elements chosen to define the expression patterns obtained from the microarray analysis two have been previously identified to be involved in regulation of gene expression. The motif GATGAC, identified in CoReg analysis as a regulatory element present in all except the most highly downregulated module SR5, is part of two regulatory elements documented in the PlantCARE database: the As-1-box of tobacco (PlantCARE ID: NT~as-1-box) and OCS-element of *Arabidopsis *(PlantCARE ID: AT~ocs-element). These were both identified as being involved in the induction of gene expression in response to salicylic acid, auxin and oxidative stress [50-54]. The alternative oxidase gene (*Aox1a*) is a member of SR4, contains this GATGAC element and transcript abundance of *Aox *is known to be induced by salicylic acid in several species [35,55,56].

## Conclusion

Using a large number of plant microarray analyses to help pinpoint the mechanisms of gene regulation is limited by the range of tools currently available. We have developed *ModuleFinder *to identify sets of genes and treatments that in our hands contain more biologically related functions for analysis of the mechanisms behind co-expression in non-linear-related sets. We then developed *CoReg *to link the clustering tree of expression-based relationships in these gene sets with frequency tables of promoter elements. These sets of promoter elements represent putative CAREs for sets of genes, and can then be tested experimentally. We consider these tools, both built on an open source software product, provide a valuable alternative tool to those widely available for the prioritisation of promoter elements for experimental analysis.

## Methods

### Data sources and processing

The changes in gene expression in response to the addition of various compounds to *Arabidopsis *suspension cells were measured as outlined previously [32]. Data for the addition of chitin to 50 mg/mL (Sigma, Sydney) and flagellen22 peptide to 1 μM (Auspep, Parkville, Victoria) are included here and arrays were carried out as described in Clifton et al. 2005 [32]. Average gene expression levels were calculated across replicate chips; in each case, a minimum of two replicates was available. For each experimental variable or time point, the log ratio of expression under experimental conditions to appropriate control conditions was determined for each gene. These log ratios formed the input for *ModuleFinder *and *CoReg *analysis. Only a subset of the >22,000 genes on the Affymetrix gene chips were analyzed in the examples presented here. This gene subset comprised 374 genes, derived from a set of proteins identified in isolated *Arabidopsis *mitochondria by liquid chromatography-tandem mass spectrometry [30]. For *CoReg *analysis, promoter sequences were taken as the 3000 base-pair sequences upstream of each gene, retrieved from TAIR.

### Programming in R

*ModuleFinder *and *CoReg *were developed in *R*, a computer language and environment for statistical computing [57]. An advantage of *R *is that it is available as free software and runs on a wide variety of UNIX platforms and similar systems, Windows and MacOS. Most importantly *R *provides a variety of built-in statistical and graphical techniques, including a variety of cluster analysis methods and facilities for displaying cluster trees and heat maps, while also allowing users to extend *R*'s capabilities by defining their own functions.

### Statistical methods used in ModuleFinder

*ModuleFinder *filters out the genes whose expression did not change under all experiments in the initial subset. This is done by considering a matrix of p-values provided by the user, which reflects the results of a test for differential expression (including correction for multiple testing if appropriate), and filtering out all genes whose p-values are above a user-defined cut-off in any of the experiments in the subset. The default p-value cut-off is 0.05, but can be set by the user to any value between zero and one. In the examples presented here, the p-values used were derived from two-sided t-tests comparing the robust multiarray analysis-processed expression measures from replicates of control and experimental conditions [5]. In each case, a minimum of two replicates was available.

*ModuleFinder *uses *R*'s hclust function for hierarchical clustering of genes based on the expression values provided in the input expression data file. The default clustering method uses a Euclidean distance measure and the Ward linkage method [58], but can be set by the user to any of the hierarchical clustering methods available in *R*. (These include Minkowski, Canberra, maximum, minimum and Manhattan distances, and the complete, single, average, centroid and McQuitty [33] methods of linkage.)

Having defined modules containing genes that are co-ordinately expressed in response to a subset of experiments, *ModuleFinder *searches for further experiments in which these modules also display co-ordinated expression responses. For each experiment not already in the module, the variance of the gene expression measures within each module is calculated using the *var *function in R (var(x_1_,.., x_n_) = sum(x_i_-mean(x))^2^/(n-1)). A small within-module variance can be interpreted as a high level of co-expression among the genes in the module. The sum of these within-module variance measures is calculated as an overall measure of how well gene expression in the experiment fits the set of modules. A measure of between-module variance is also calculated for each experiment (between-module var = sum(mean_module i _-mean_all modules_)^2^). Large values here indicate that the modules had distinct expression patterns in the experiment. The experiment that most closely 'fits' the module structure will display co-ordinated gene expression within modules and, ideally, distinct patterns of gene expression between modules. That is, it will have small within-module variances and a large between-module variance. The algorithm thus looks for the experiment with the highest ratio of between-module variance to sum of within-module variances.

### CoReg algorithm

The primary data set used by *CoReg *is a table representing the incidence of each of a list of potential sequence elements (e.g. hexamers, known motifs) in a list of gene promoters. This is a table of sequence elements on the horizontal axis, gene names on the vertical axis and values of TRUE or FALSE indicating whether or not the element was found in a search of the gene's promoter sequence. String matching is used to search for sequence elements in promoter sequences. This table can be prepared independently, or *CoReg *can build one from a list of sequence elements and a file containing gene promoter sequences in FASTA format input by the user.

The user is then asked to input a table of expression data. The genes in this table must appear in the incidence table, and must be labelled in the same way (e.g. AGI locus identifier). *CoReg*, like *ModuleFinder*, uses *R*'s hclust function for hierarchical clustering of genes based on the expression values in this table. Distance and linkage methods can be set by the user to any of those available in *R *(see above). The user is also asked to indicate branches defined by the tree that they consider to be gene expression clusters.

The resulting hierarchical clustering tree is split into two branches, separating the genes into two discrete groups (say A and B). The incidence table is then used to determine, for each sequence element in the table, the proportion of genes in each group that contain that element. This is dubbed the 'frequency' of the element in those two groups (say f_i, A _and f_i, B_, where i denotes sequence element i). These frequencies are then compared to a user-defined tolerance level, *f*. Any sequence element that occurs with frequencies f_i, A _<*f *and f_i, B _> (1-*f*) is recorded in a list of sequence elements that may be able to explain the difference in expression patterns of the two groups. The same process (splitting into two branches and searching for elements whose frequencies are different in the two groups defined by the split) is repeated for each of the two branches in an iterative procedure, stopping when the final user-defined clusters are reached.

In addition, the frequencies of each sequence element in each of the user-defined clusters is compared to a second user-defined tolerance level *g*. Any sequence elements whose frequency is below *g *or above *1-g *in exactly one of these clusters, is added to the list of interesting sequence elements.

The gene expression clusters defined by the user are then themselves clustered, according to the frequencies of all the recorded sequence elements. The same method chosen for expression-based hierarchical clustering is used at this step. The user is then given the opportunity to select subsets of the recorded sequence elements and cluster according to those, the aim being to isolate a subset of sequence elements leading to a hierarchical structure similar to that defined by the expression-based hierarchical clustering tree.

ModuleFinder and CoReg are available for downloading from [59]. Alternatively a package will be emailed on request containing program files, instruction files and examples files. We request that users cite this manuscript if using these programs.

## Abbreviations

Aox alternative oxidase

CARE(s) *cis*-acting regulatory element(s)

NDH alternative NAD(P)H dehydrogenases

NGMP nuclear genes encoding mitochondrial proteins

TOM translocase of the outer mitochondrial membrane

TIM translocase of the inner mitochondrial membrane

## Competing interests

The author(s) declare that they have no competing interests.

## Authors' contributions

KEH was responsible for designing and writing the code and analyzing the data. AHM and JW contributed in designing the project, obtaining the expression data, interpretation of analysis and writing the manuscript.

## Supplementary Material

Additional File 1 **Supplementary Figure **1A A .eps file with a supplementary figure referred to in text Click here for file

Additional File 2 **Supplementary Figure **1B A .eps file with a supplementary figure referred to in text Click here for file

Additional File 3 **Manual.pdf **A manual that describes how to install and use programs and the outputs they produce. Click here for file

Additional File 4 **MF and CoReg code **A .zip file with containing the code Click here for file

Additional File 5 **MapMan files.zip **Annotation files for visualisation in Mapman Click here for file

Additional File 6 **User guide (htm files).zip **Instruction for use in htm format Click here for file
