# Supplementary material for: ModuleFinder and CoReg: alternative tools for linking gene expression modules with promoter sequences motifs to uncover gene regulation mechanisms in plants
Source: Plant Methods. 2006 Apr 11;2:8. doi: 10.1186/1746-4811-2-8 (PMC1479336; doi:10.1186/1746-4811-2-8)
Supplement: Additional File 3 — Manual.pdf A manual that describes how to install and use programs and the outputs they produce. [file 1746-4811-2-8-S3.pdf]

ModuleFinder and CoREG

Complementary R-based programs for the identification of co-ordinated gene expression modules and promoter elements involved in the co-regulation of co-expressed genes.

General Installation **2**

ModuleFinder
- ***About ModuleFinder*** **3**
- ***ModuleFinder Tutorial*** **9**
- ***Using ModuleFinder*** **15**

CoREG
- ***About CoREG*** **23**
- ***CoREG Tutorial*** **26**
- ***Using CoREG*** **35**
- ***CoREG and MapMan*** **41**

Disclaimer

These programs were created for the analysis of small subsets of genomic expression data from microarray experiments in Arabidopsis, as part of an Honours project in the Plant Molecular Biology Group in the Department of Biochemistry and Molecular Biology at the University of Western Australia. The Honours thesis can be downloaded here.

Although the principles and methods employed in this analysis are applicable to other data sets, the programs have not been tested with data sets involving more than 1000 genes or more than 300 microarray experiments, or data from other organisms. Currently, CoREG is not designed for use with genes other than those from Arabidopsis, labelled with their Agi locus identifier (ATxGxxxxx). Anyone interested in using CoREG with other genomes can make the necessary modifications to the code themselves, or request help from me.

Any queries, email Kathryn Holt at pup2308-modules@yahoo.com.au.

Installation

Before you can run ModuleFinder and CoREG you will need to do the following:

1) Install a copy of the statistical package R. You can download the latest version from http://cran.r-project.org/mirrors.html, where you'll also find installation instructions and additional help with R.
2) Install a number of R packages. You can download packages from CRAN and Bioconductor from within R, via the Packages menu.
    a. To use the Windows-based graphical interface for either program you will need the package *tkWidgets* from Bioconductor.
    b. To use ModuleFinder you will need the packages *gregmisc* from CRAN, and *ctc* from Bioconductor.
    c. To use CoREG you will also need the following packages from CRAN:
        i. *cluster*
        ii. *combinat*
        iii. *e1071*
        iv. *gregmisc*
        v. *maptree*
        vi. *rpart*

Using the programs

Each time you want to use the programs, you will need to first open R, then select 'Source R code…' from the 'File' menu, and locate the file "ModuleFinder.R" and/or "CoREG.R" (Windows) or "ModuleFinderMAC.R" and/or "CoREGMAC.R" (Mac). You will then be asked to locate your data file(s) and set some parameters.

For details, read the tutorials and user guides for ModuleFinder or CoREG.

ModuleFinder Overview

Why ModuleFinder?

What are the benefits?

How does it work?

Overview

Schema

Specifics

Why was ModuleFinder created?

In order to better characterise the transcriptional responses of various pathways under a variety of conditions, it is desirable to define subsets of genes whose expression is co-ordinated during the response to particular subsets of conditions. In this way, the relationships between conditions can be linked to the co-ordinated expression of subsets of genes, giving a much clearer picture of the mechanisms of responses to various conditions at the level of gene expression. It is also desirable to be able to ask specific questions about any similarity in expression responses to a subset of conditions of interest, for example, which genes are regulated in the same way in response to two different sorts of abiotic stresses. To accomplish these aims, there is a need for a method of analysis of gene expression data that allows experiments and genes to be analysed together in an integrated way, capable of identifying subsets of both genes and experiments under which gene expression is co-ordinated, despite a lack of overall co-ordination across the entire data set. The resulting subsets of genes would thus constitute gene expression modules specific to particular subsets of conditions, which would arguably be more easily interpretable and more biologically significant than gene clusters derived from simple clustering methods across a broader set of experiments.

What are the benefits?

1. Shared responses can be lost in simple cluster analysis

Cluster analysis is a useful technique for identifying genes whose expression patterns across a given set of stresses are similar. For example, the analysis will cluster together all those genes whose expression is upregulated in response to conditions A, B and C, downregulated in response to conditions D, E and F, and unaffected by conditions G and H. However since expression data from *all* experiments is used in the analysis, this cluster won't include genes that are upregulated in response to A, B, C and G, and downregulated in response to D, E, F and H. These will be grouped together into a separate cluster since their expression patterns differ under stresses G and H. The similarity between the clusters in response to stresses A to F is lost in the analysis. Yet from a biological point of view, the fact that both clusters display co-ordinated expression in response to stresses A to F is very interesting. It may indicate that they are co-regulated by a factor that is induced or activated under stresses A-C and repressed or inactivated under stresses D-F. Thus it would be informative to

identify both clusters of genes, and the stresses A-F, as a gene expression module. Such a module is more likely to have biological significance than the two separate groups produced by cluster analysis.

Shared gene expression responses can be split up in simple cluster analysis

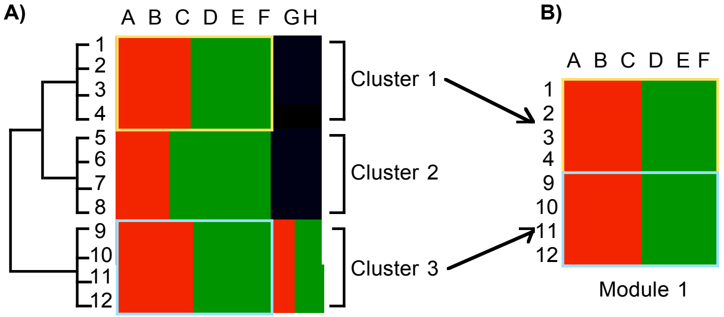

A) Cluster analysis groups together genes whose expression patterns are similar across all available experiments. Thus cluster analysis of genes 1 to 12 in treatments A to H above, splits the genes into the three separate clusters shown in the figure. The splitting of genes 1-4 and 9-12 into separate clusters is due only to their differential expression in experiments G and H. The shared expression response of Cluster 1 and 3 genes, very interesting from a biological point of view, is lost in this analysis.
B) Clusters 1 and 3 (genes 1-4, 9-12) are co-ordinately expressed in response to treatments A-F, thus this combination of genes and treatments can be identified as a single gene expression module in an alternative method of analysis.

2. Existing approaches are inadequate for identifying shared responses among numerous non-linear-related sets of conditions

The majority of methods that involve analysis of both genes and samples from microarray data are aimed at identifying genes that can discriminate between two or three groups of samples. These are generally driven by the needs of cancer researchers, who wish to identify genes that can be used in diagnostic or prognostic tests in cancer patients. Unfortunately these methods are not appropriate for analysing sets of conditions that do not fall into discrete categories which aren't related in any linear way such as a time course or multiple dilutions of one or two treatments. In this case, to identify gene expression patterns that are shared among subsets of the conditions requires the analysis of genes and samples to be integrated in a way that doesn't depend on defined relationships between the samples.

A handful of techniques have been developed to achieve this, including coupled two-way clustering (*CTWC*) and conserved gene expression motifs (*XMotif*). *CTWC* works by clustering genes into subsets, then clustering samples into subsets. Each gene subset-sample subset pair is then considered as a submatrix and genes and samples are re-clustered within that submatrix (Getz et al., 2000). The result is a collection of subsets of genes and samples (gene expression modules), which theoretically should display co-ordinated expression patterns. However, this fragmentation of the data into small discrete modules makes it difficult to interpret the results and particularly difficult to see overall trends in the expression patterns. *XMotif* also attempts to

identify subsets of genes and samples in which the expression of the genes is co-ordinated, but using a much more complicated algorithm (Murali and Kasif, 2003). The computer code for this algorithm was not available for this study but, like *CTWC*, this method is liable to produce a large set of fragmented modules that is difficult to interpret. ModuleFinder was developed, with the aim of identifying gene expression modules in a way that facilitates interpretation of results. It also needed to allow easy visualisation not only of the expression patterns of discrete modules, but also of the relationships between the modules.

How does it work?

1. General procedure

a) Input

The algorithm takes as its input a matrix of summarised expression data from a set of experiments. The data should be average log ratios from replicate experimental and control samples. It also requires a matrix of p-values associated with each data point, providing an estimate of how likely the observed gene expression values would be if there was really no change in experimental compared to control conditions. These must be calculated from the original microarray data using t-tests or similar, and loaded into ModuleFinder.

b) Processing

The algorithm begins with a subset of experiments and extracts the genes whose expression changed in those experiments. It then clusters the genes and splits them into co-expressed modules. Next the algorithm searches for another experiment (outside the initial subset) which fits the expression patterns of these modules. The new experiment is added to the module and the genes are re-clustered. Experiments are added one by one in an iterative procedure of searching for matching experiments and re-clustering the genes, until no more can be found that fit the module expression patterns. The resulting subsets of genes and experiments are referred to as gene expression modules, as they define not only gene clusters but subsets of genes whose expression is co-ordinated under a subset of experiments. The algorithm is thus named ModuleFinder.

c) Output

The output of the algorithm is a PDF file containing clustering trees and expression heat maps produced after the addition of each new experiment. It also includes pie charts displaying the breakdown of each module according to the functional categories of its member genes. Figure 3.2 provides a graphical representation of the algorithm.

2. Schema

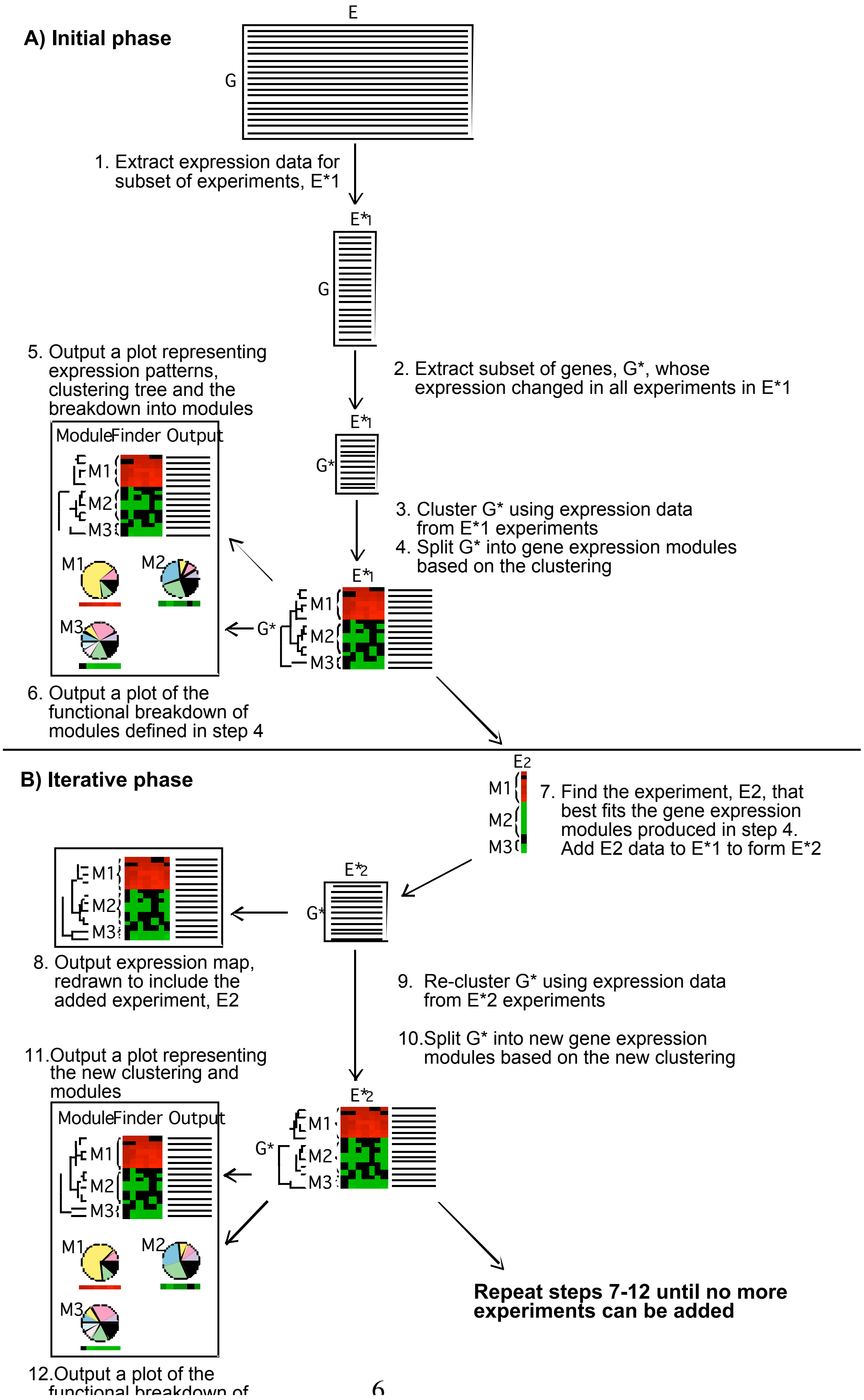

A) Initial phase
E
G
1. Extract expression data for subset of experiments, E*1
E*1
G
2. Extract subset of genes, G*, whose expression changed in all experiments in E*1
E*1
G*
3. Cluster G* using expression data from E*1 experiments
4. Split G* into gene expression modules based on the clustering
E*1
M1
M2
M3
G*
5. Output a plot representing expression patterns, clustering tree and the breakdown into modules
ModuleFinder Output
M1
M2
M3
M1
M2
M3
6. Output a plot of the functional breakdown of modules defined in step 4
B) Iterative phase
E2
M1
M2
M3
7. Find the experiment, E2, that best fits the gene expression modules produced in step 4. Add E2 data to E*1 to form E*2
E*2
G*
M1
M2
M3
8. Output expression map, redrawn to include the added experiment, E2
9. Re-cluster G* using expression data from E*2 experiments
10.Split G* into new gene expression modules based on the new clustering
11.Output a plot representing the new clustering and modules
E*2
G*
M1
M2
M3
ModuleFinder Output
M1
M2
M3
M1
M2
M3
Repeat steps 7-12 until no more experiments can be added
12.Output a plot of the
functional breakdown of

3. Specifics

a) Initial phase: identifying gene expression response patterns shared by an initial subset of experiments (See figure A above)

1. The algorithm begins with a subset of one or more experiments, which can be user-defined or selected by the algorithm. If no subset is defined by the user, the algorithm will select the pair of experiments that are most highly correlated across the whole gene set.
2. The algorithm filters out the genes whose expression did not change under all experiments in the subset. This is done by considering the matrix of p-values provided by the user, and filtering out all genes whose p-values are above a set cut-off in any of the experiments in the subset. The default p-value cut-off is 0.05, but can be set by the user to any value between 0 and 1.
3. The remaining genes, whose expression changed under all experiments in the subset, are clustered based on their expression levels in those experiments. The default clustering method uses a Euclidean distance measure and the complete linkage method, but can be set by the user to any of the hierarchical clustering methods available in *R*. (These include Minkowski, Canberra, maximum, minimum and Manhattan distances, and the complete, single, average, centroid, Ward and McQuitty methods of linkage.)
4. The resulting clustering tree is used to split the genes into a given number of clusters. The number of clusters can be user-defined, or chosen by the algorithm so that the number of clusters is closest to the average number of genes in each cluster.
5. The clustering tree and corresponding expression heat map is drawn in the PDF output file. In the figures produced, each gene is labelled by the module it appears in, its functional category, its Agi locus and a descriptive name.
6. The combination of each gene cluster and the experiment subset produces a gene expression module. For each module, the algorithm draws in the PDF file a pie chart representing the functional breakdown of its member genes. Below the pie charts, expression heat maps for the corresponding module are displayed.

b) Iterative phase: identifying further experiments that share the identified gene expression response patterns (See figure B above)

7. Having defined modules containing genes that are co-ordinately expressed in response to a subset of experiments, the algorithm searches for further experiments in which these modules also display co-ordinated expression responses. For each experiment not already in the module, the variance of the gene expression measures within each module is calculated. A small within-module variance can be interpreted as a high level of co-expression among the genes in the module. The sum of these within-module variance measures is calculated as an overall measure of how well gene expression in the experiment fits the set of modules. A measure of between-module variance is also calculated for each experiment. Large values here indicate that the

modules had distinct expression patterns in the experiment. The experiment that most closely 'fits' the module structure will display co-ordinated gene expression within modules and, ideally, distinct patterns of gene expression between modules. That is, it will have small within-module variances and a large between-module variance. The algorithm thus looks for the experiment with the highest ratio of between-module variance to sum of within-module variances.

8. The clustering tree already defined is redrawn in the PDF file, beside a heat map including expression data from the new experiment.
9. The genes are then re-clustered based on expression data from the new experiment as well as the initial experiment subset. The same clustering method is used in the initial and iterative phases.
10. The new clustering tree is then used to split the genes into a given number of clusters. The number of clusters is the same in the initial and iterative phases.
11. The new clustering tree and corresponding expression heat map is drawn in the PDF file.
12. Pie charts representing the functional breakdowns of the new modules are drawn in the PDF file.

Steps 7 to 12 are repeated until no more experiments can be found that have a sufficiently high ratio of between-module to within-module variance. The default cut-off value for the ratio is 4, but can be user-defined.

Getz, G., Levine, E., and Domany, E. (2000). Coupled two-way clustering analysis of gene microarray data. Proc Natl Acad Sci U S A **97,** 12079-12084.

Murali, T.M., and Kasif, S. (2003). Extracting conserved gene expression motifs from gene expression data. Pac Symp Biocomput**,** 77-88.

ModuleFinder Tutorial

This tutorial will take you step-by-step through a run of ModuleFinder on a Windows PC, using a set of data from stress-related experiments from AtGenExpress. You will need to locate the two data files that came with ModuleFinder – “AtGenExpress_DataLogR.txt” and “AtGenExpress_Pvals.txt”.

You should already have installed R and the necessary packages as outlined in the installation guide.

1. Create a new folder, call it something like ‘ModuleFinder Tutorial’ and copy the two data files into it.
2. Open R.
3. Set the current directory in R to the one you just created in step 1.

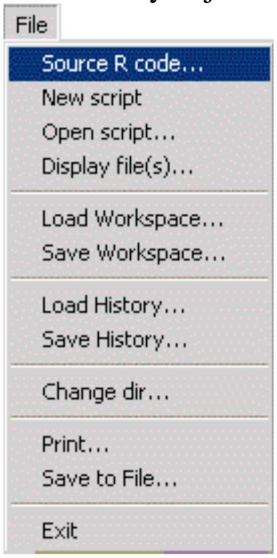

4. Load in the ModuleFinder R code, by going up to the file menu and selecting “Source R Code…”

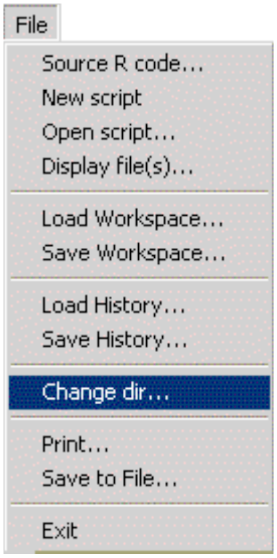

5. R will now load in the packages it requires. If you don't have necessary packages installed, do this now.
   (See the installation guide for details.)

6. A window will pop up, asking you to locate the data file you want to load.
   If you followed Step 1 and set the current directory correctly, you should only see the two AtGenExpress files. Double-click on the data file, then click Finish:

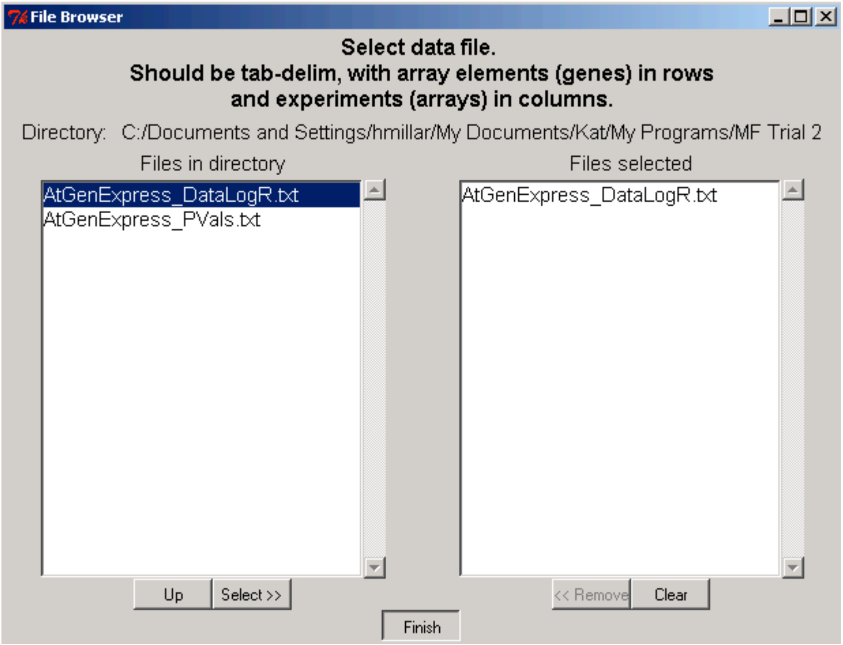

7. A second window will pop up, this time asking you to locate the file of p-values. Double-click the PVals file and click Finish.

8. Another window will pop up to say you need to indicate which columns contain gene information rather than data. Click OK.
In the window that comes up, on the left you will see a list of the names of all the columns in the data file. The first 5 contain information about the genes, the rest contain average log ratios of the experimental vs control conditions from lots of different experiments. Select the first 5 column names, and move them over to the right hand box by clicking on "Select >>". Then click Finish:

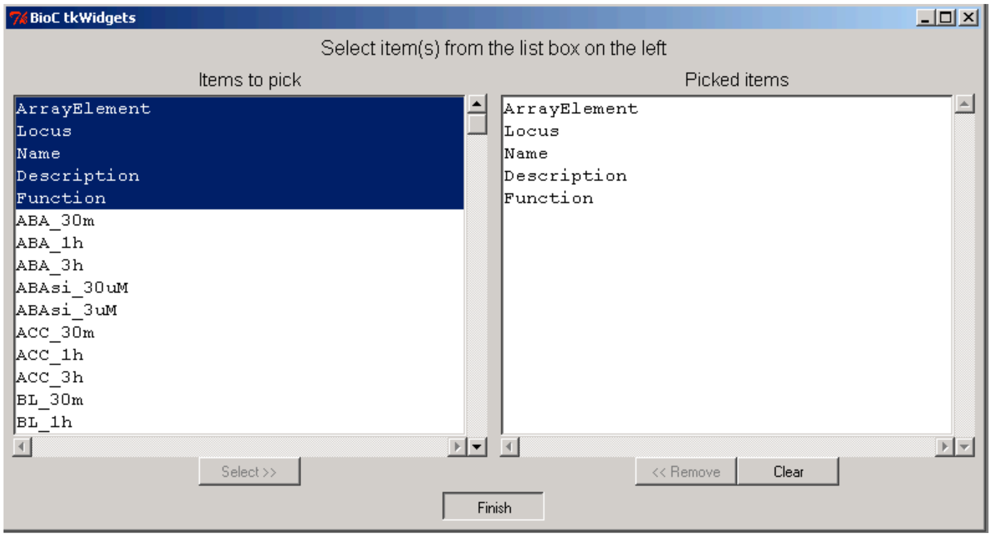

9. You will now be asked to select a combination of these gene information columns which together will provide an informative, unique label for each gene. Since the data in this file is from Affymetrix GeneChips, on which single genes can be represented by more than one array element, the Locus alone will not be a unique label. So select Locus, then ArrayElement, and then Name, to create our unique label. (It's handy to include Name here, so you can more easily recognize genes of interest in the output files.)

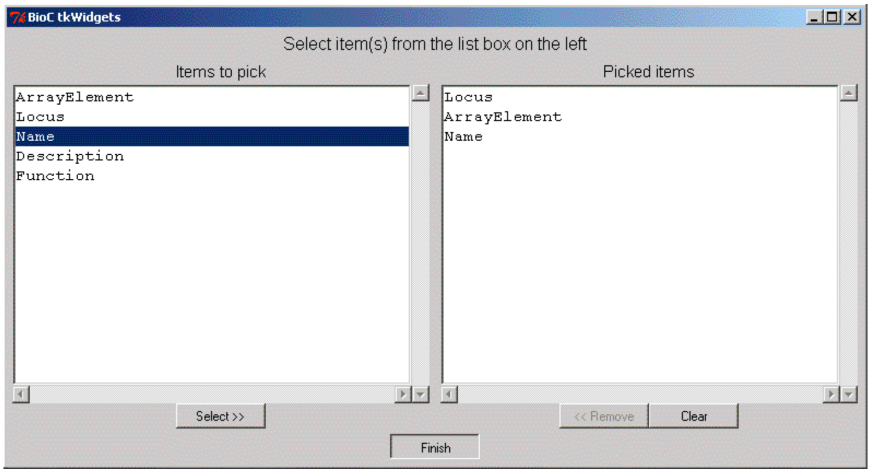

10. Next you will be asked to select a starting set of experiments. You can try different combinations of experiments later, but for this example scroll down and double-click on “Osmotic_Shoot_24h” and then “Salt_Shoot_24h”. Click Finish.

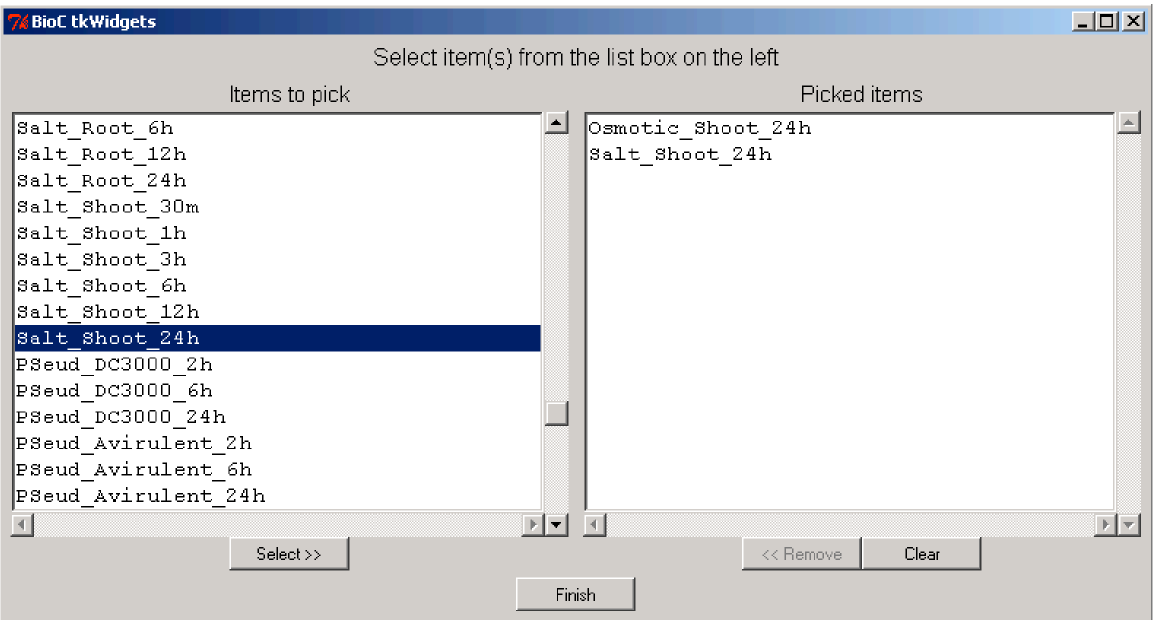

11. Now you will be given the opportunity to change some parameters. Change the “Name for output files” to something like “Tutorial”. The names of any files created by ModuleFinder will begin with this title. Change the maximum p-value to 0.1. Leave the remaining parameters as they are.

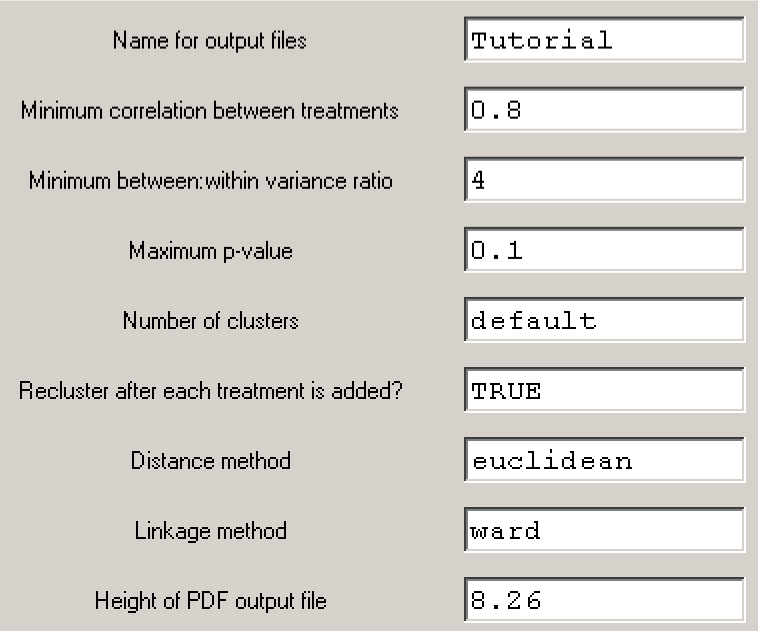

12. Now ModuleFinder will begin to do it's work! Firstly, it will identify the genes with p-vals<0.1 in both the experiments we selected. On the R console, it will print out the number of genes that passed this criteria (in this case 133), and then list these genes (using the unique labels we specified at step 9):

```
number of genes: 133
Selected genes:
 AT2G39795 245063_at ND
 AT2G39725 245064_at ND
 AT2G40890 245101_at CYP98A3
 AT2G47490 245152_at ND
 AT2G33210 245164_at HSP60-2
... ... ... ... ... ... ...
```

13. These genes will then be clustered according to their expression in the two experiments. The number of clusters is printed to the console. This combination of genes and experiments is the beginning of the module identification.

    Then, ModuleFinder will begin to search for other experiments in which these gene modules behave as modules, that is experiments in which the expression of genes in the same module are similar, but genes in different modules are expressed differently. Experiments are added one-by-one, and the progress is reported on the console:

```
Treatments:  Osmotic_Shoot_24h  and  Salt_Shoot_24h
number of clusters: 12
Adding variable 'Osmotic_Shoot_12h'. Between-module/Within-module variance:
14.81115
Adding variable 'Salt_Shoot_12h'. Between-module/Within-module variance: 7.564039
Adding variable 'ColdShoot1h'. Between-module/Within-module variance: 6.71945
Adding variable 'Osmotic_Shoot_6h'. Between-module/Within-module variance:
6.402471
Adding variable 'ABA_1h'. Between-module/Within-module variance: 5.320692
```

14. When ModuleFinder can't find any more experiments to add to the modules, it will report this and end the run:

```
No more treatments to add
null device
          1
>
```

    There may be warning messages reported at this point too. Ignore them.
    The red pointer indicates that R has finished what it was doing and is ready to go again.

15. You are now ready to look at the ModuleFinder output. A number of files will have appeared in the directory you created at step 1. Open the PDF file first. The first page reports what the starting parameters were:

MODULEFINDER RESULTS
Analysis name: Tutorial

Starting treatments: Osmotic_Shoot_24h and Salt_Shoot_24h
Minimum between/within cluster variance ratio for adding treatments: 4
Clustering parameters: euclidean distance, ward linkage
P_value cut_off for genes: 0.1

The second page will show a clustering tree and heatmap for the genes that were differentially expressed (p<0.1 in the p-values file) in both of the starting experiments. The next few pages will contain pie charts displaying the functional breakdown of each of the resulting modules, with the corresponding heatmaps printed below.
After this, you will see a new heatmap, which now includes the first additional experiment to be added into the modules, followed by the new clustering tree that results from reclustering the genes using data from the three experiments now in the module, followed by new pie charts for the resulting modules. The same output is printed for each added experiment. Finally, the last page contains a heatmap displaying the expression of the module genes in the experiments that didn't end up in the module.

16. Go to page 80, where the experiment "ABA_3h" was the last to be added. We'll take this subset of genes and experiments as the final result. (If you have a look at the next few experiments that were added, you'll see that the changes in expression levels were very very low, so we'll ignore these.)

17. Now go back to the directory containing the ModuleFinder output. This contains cluster files (for viewing in TreeView and similar programs, extensions .cdt and .gtr) for each stage of the ModuleFinder run. Start TreeView (http://jtreeview.sourceforge.net) and use it to open the file ending in "AddABA_3h.cdt".
Select "Pixel settings…" from the Settings menu. For consistency with ModuleFinder, CoREG and MapMan output it's best to change the default colour settings so that blue is positive and red is negative. You will also need to adjust the contrast in order to resolve the expression measures (since the data was logged the values are quite small). Set the contrast to 1 and close the settings window. You can now explore the genes, clustering tree and experiments in TreeView, and save any heatmap/clustering images or gene lists for all or part of the tree.

18. ModuleFinder also created files containing the expression data for the gene and experiment subsets at every stage. The file ending with "GeneClusters_AddABA_3h.csv" contains the data for our final modules, and you can now use this file to explore promoter sequence elements in CoREG.

Using ModuleFinder

This guide explains how to use and interpret ModuleFinder.
For a step-by-step example, see the Tutorial first.
You should read the Installation Guide first.

Preparation
Running ModuleFinder
ModuleFinder Output

Preparation

1. Make sure you have your data ready.
   You will need to have two files to run ModuleFinder, which should both be in ***tab-delimited format*** (Excel can save files in this format):
   a. File 1: Expression data from a set of experiments.
      1. Each row in this file should correspond to a gene.
      2. The first few columns should contain gene information, for example the gene name, array element, locus (Agi identifier), gene description and functional category.
         a. If you want to use your ModuleFinder results with CoREG, one of these columns must contain the *Arabidopsis* Agi locus identifier. This column should be labeled "Locus".
         b. One or a combination of these columns must result in a unique identifier for each gene. Note that a single gene locus may be represented by multiple elements on an array. Thus a locus identifier alone may not provide a unique identifier for every data point. However, the combination of locus and array element will provide an informative, unique identifier for every element in your data set, so it is a good idea to have at least these two columns in your data file.
         c. When ModuleFinder creates clustering files for viewing TreeView, it will label genes using the columns named "Locus" and "Description", so if you want to use these files you'll need to make sure you name your columns appropriately.
         d. ModuleFinder will attempt to provide pie charts displaying the functional breakdown of the modules it identifies. However it can only do this if you provide a column in your data file indicating a functional category for each gene.
      3. The remaining columns should contain summarized experimental data from a number of array experiments. It will work best if this is the ***average log ratio*** of gene expression under two or more replicates of experimental vs control conditions.

An example file:

ArrayElement	Locus	Name	Description	Function	ABA_30m	ABA_1h	ABA_3h
245168_at	AT2G33150	ACoAAT-2	acetyl-CoA C-acyltransferase	Metabolism	4.47E-02	1.51E-02	0.27889
263348_at	AT2G05710	ACON-1	aconitate hydratase	Energy	-1.64E-02	1.39E-02	0.15939
253954_at	AT4G26970	ACON-2	aconitate hydratase	Energy	-1.76E-01	-9.51E-02	-0.2532
253135_at	AT4G35830	ACON-3	aconitate hydratase	Energy	1.53E-02	-6.64E-02	-0.0762
256275_at	AT3G12110	ACT11	actin 11	Cellular Structural	4.04E-02	-2.43E-01	-0.2106
252531_at	AT3G46520	ACT12	actin 12	Cellular Structural	-1.43E-02	-2.06E-02	-0.0172
250458_s_at	AT5G09810	ACT2/7	ACTIN 2/7	Cellular Structural	-8.37E-03	-8.62E-02	-0.1998
AFFX-r2-At-Acti	AT5G09810	ACT2/7	ACTIN 2/7	Cellular Structural	2.01E-02	-7.29E-02	-0.1516
247736_at	AT5G59370	ACT4	actin 4	Cellular Structural	8.67E-02	1.32E-03	-0.0328
258680_at	AT3G08580	ANT-1a	adenylate translocator 1	Cellular Transport	-3.47E-03	-1.38E-02	-0.1347
258452_at	AT3G22370	AOX1a	alternative oxidase 1a	Energy	2.91E-01	8.98E-01	1.74759
258451_at	AT3G22360	AOX1b	alternative oxidase 1b	Energy	2.30E-01	2.06E-01	0.28449
258235_at	AT3G27620	AOX1c	alternative oxidase 1c	Energy	8.37E-02	-8.66E-03	-0.0427
260706_at	AT1G32350	AOX1d	alternative oxidase 1d	Energy	7.90E-02	-3.48E-03	0.14193
247269_at	AT5G64210	AOX2	alternative oxidase 2	Energy	4.94E-02	2.51E-02	0.75618

b. File 2: P-values corresponding to the expression data.
   1. This should have the exact same layout as the expression data file, with the genes in rows, and gene information and experimental data in columns. ***All rows and columns should be in the same order as the expression data file.***
   2. The experimental data columns should contain p-values from a statistical test of differential gene expression between the replicated experimental and control samples.

2. Set the directory in which to save ModuleFinder output.
   a. This is done within R, by choosing "Change dir…" from the File menu.

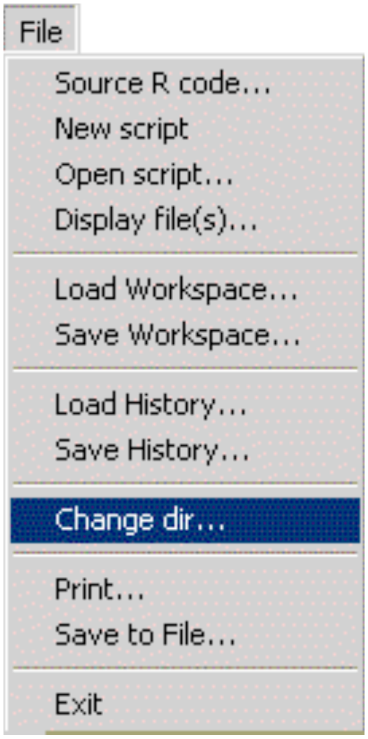

   b. ModuleFinder can create a lot of output files, so it is a good idea to create a new directory for this purpose. Perhaps the best way to organize your use of ModuleFinder is to create a new folder, and copy your expression

data and p-value files into it. Then, set it as the current R directory. If you are using the Mac version you ***have to*** set the current directory to the one containing the data files.

Running ModuleFinder

1. To run ModuleFinder with your data, in R select 'Source R code…' from the 'File' menu, and locate the file "ModuleFinder.R" (GUI version for Windows) or "ModuleFinderMAC.R" (for running on Mac). This will load all the ModuleFinder functions into R, ready for using.

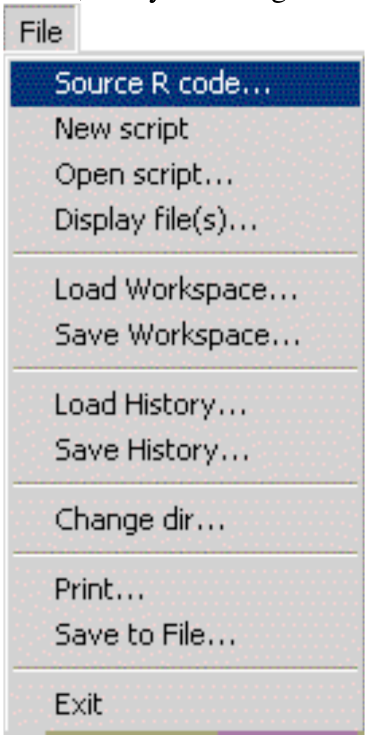

2. You will then be asked for a series of inputs, including the location of your data files and some additional parameters.
   a. In the Windows version, this will be via dialogue boxes which allow you to make selections or enter information.
   b. In the Mac version this will be partly via dialogue boxes and partly via the command line, where questions will be printed, along with some guidance about how to answer them. To respond to these questions you will generally need to enter either a number, a name or a vector of these, then press RETURN.
      In R, all text must be surrounded by quotes to be recognized as such. (e.g. `"text"` or `"datafile.txt"`). Numbers can be entered as-is (i.e. `5` not `"5"`). Vectors in R are surrounded by brackets and preceded by a lowercase 'c', e.g. `c(1,2,3)` or `c("Exp1","Exp2")`.

3. You will first be asked to locate your expression data file, followed by your p-value file.

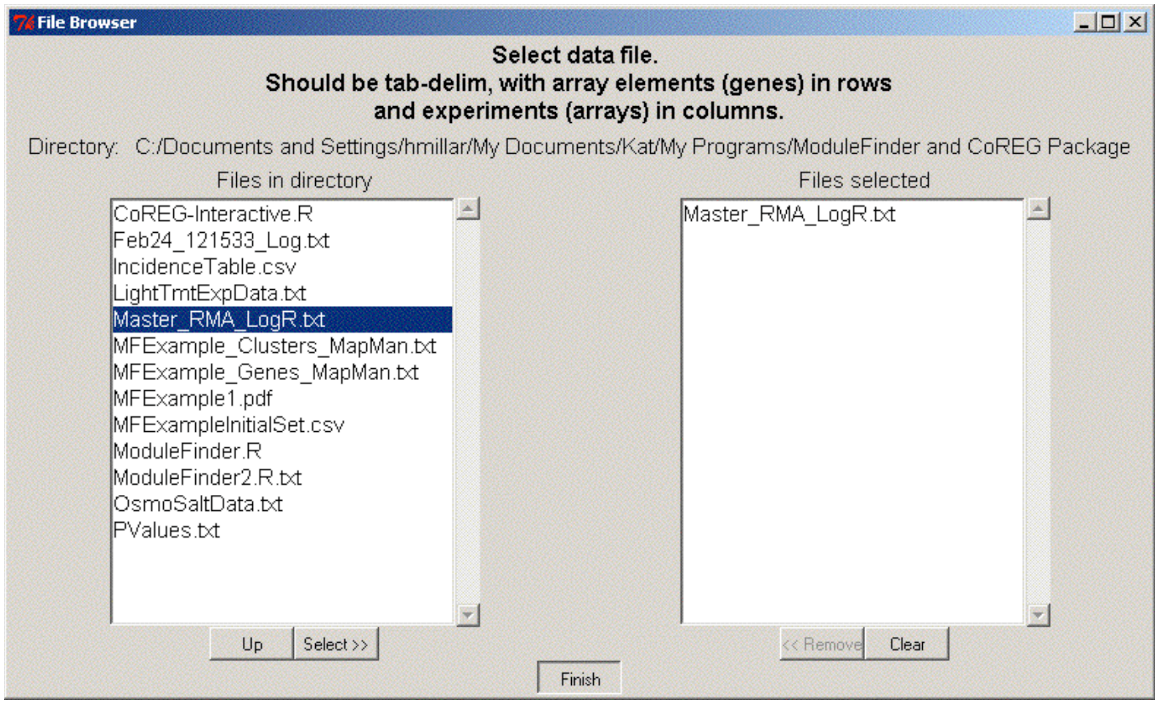

4. Next you will be asked to indicate which columns contain gene information rather than data. For example, in the data file set out above, the file begins with 5 columns containing gene information – Array Element, Locus, Name, Description and Function.

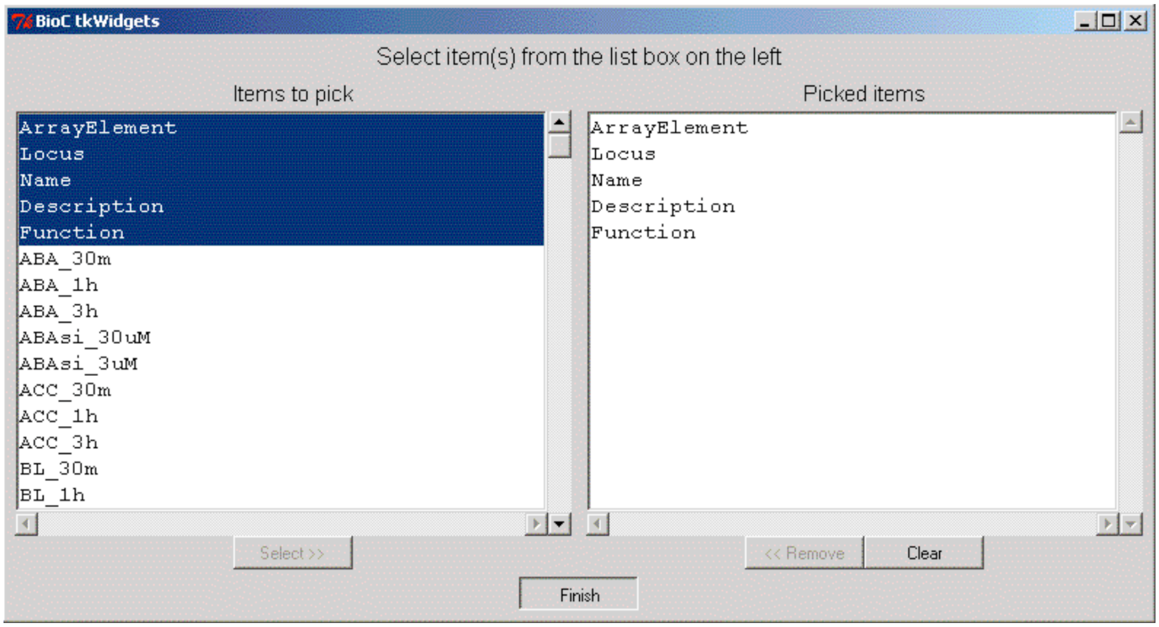

5. Next you will be asked to indicate a combination of these columns which results in a unique identifier for each gene. This is also how the genes will be labeled in the output files, so choose a combination that will uniquely and informatively label each data point. A good choice is the locus, array element and name or description, as the locus and name/description will help you to recognize interesting genes, while the array element will ensure labels are unique.
    a. In the Windows version, you are presented with a list of the columns containing gene info, and asked to select a combination of these. Note that that the order you select them in is the order they will appear in gene labels.

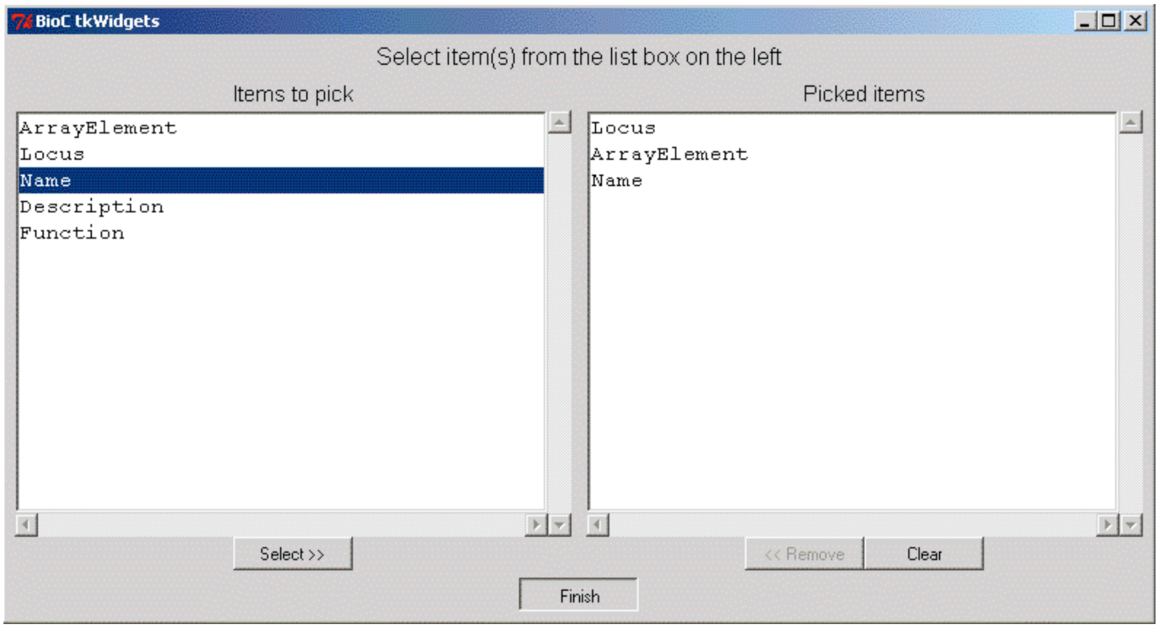

    b. In the Mac version you will need to type a vector indicating the columns that you want to use. In R, vectors are surrounded by brackets and preceded by a lowercase c, like this:
    So if your gene info columns are like this:
        Column 1 = Array Element
        Column 2 = Locus
        Column 3 = Name
    then entering:
        `c(2,1,3)`
    will ensure data points are labeled by locus identifier, array element and gene name (in that order).

6. Next you will be asked to select an initial subset of experiments to run ModuleFinder with. To do an unsupervised run, leave this blank. (See the ModuleFinder Overview for more information about this step.)

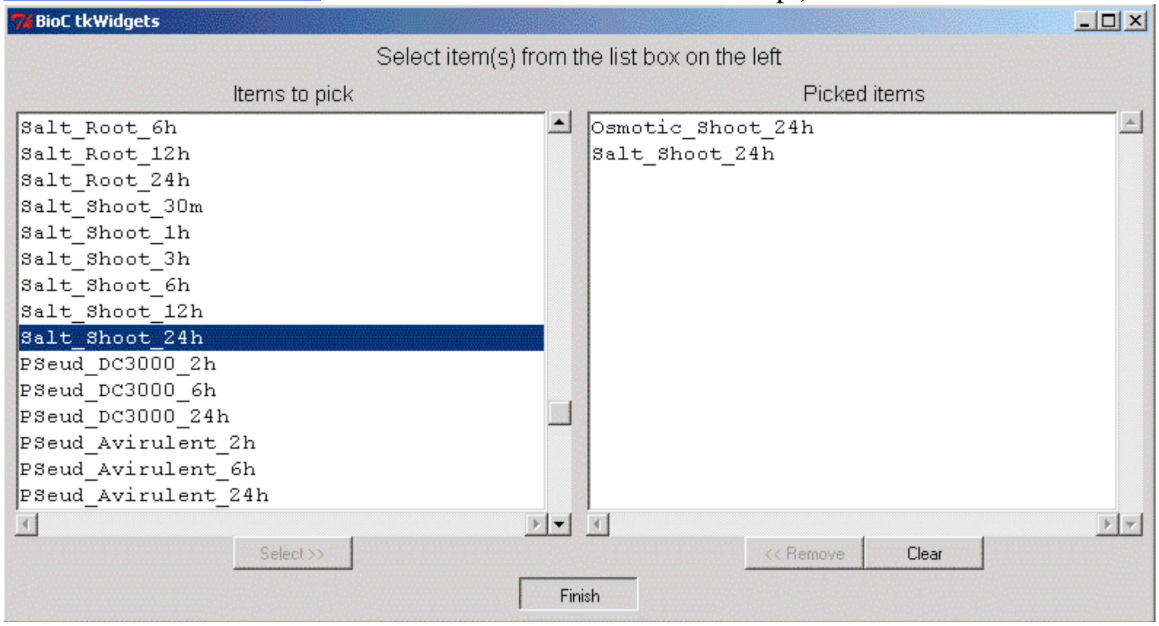

7. Finally, you will asked to set a number of parameters. In the Windows version, this will be via a single dialogue box in which you can leave the default values or enter your own.

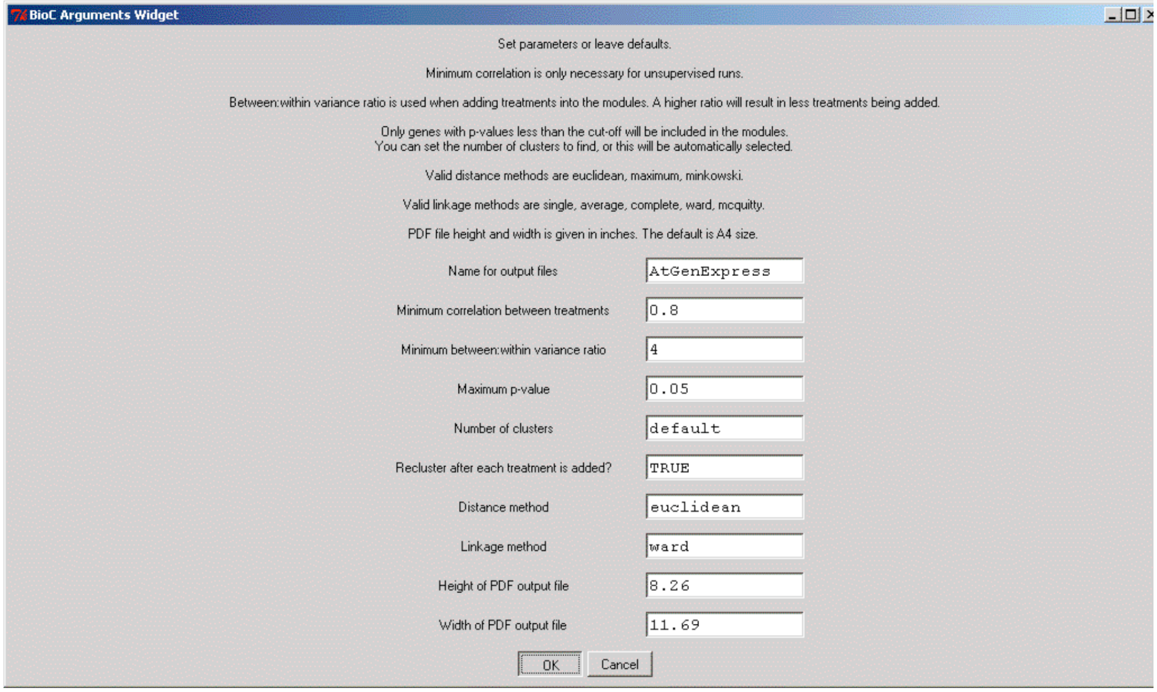

In the Mac version you will be asked about each parameter in turn via the command line. There is also some guidance on the command line about responding to these prompts.

a. *Name for output files*. The ModuleFinder output files will begin with whatever you enter here. It is helpful for keeping track of which files belong to which run of ModuleFinder.
b. *Minimum correlation between treatments*. Relevant only to unsupervised runs. ModuleFinder will be run on every pair of treatments whose raw correlation, across all elements in the data set, is above this cut-off. Depending on how similar your various experiments are, you may want to raise or lower the default value.
c. *Minimum between:within variance ratio*. ModuleFinder chooses which new experiments to add into a module by comparing how co-ordinated the expression of the current gene clusters are in all experiments not already in the modules. The ratio of between-cluster variance to within-cluster variance is used to assess this. There is a bias here towards the selection of experiments in which not only are genes within clusters behaving in a co-ordinated way, but in a way that is different from the behaviour of the other clusters. Therefore the actual value of this ratio will creep ever lower as the number of genes and number of clusters increases. It is a good idea to start with the default value, check the results, then tweak it if you think it necessary.
d. *Maximum p-value*. Only the genes whose p-values are below this cut-off in the initial experiment set will be included in the subsequent modules. If you find the default value leads to too many or too few genes being included, try changing this value.
e. *Number of clusters*. By default, ModuleFinder will split the genes into the number of clusters closest to the square root of the number of included genes. This is an attempt to balance the number and size of the gene clusters that are produced. However, if you find that it is splitting up some decent looking clusters, or lumping together some groups that are internally variable, you can specify here the number of clusters that you want to break the clustering tree into.
f. *Recluster after each treatment is added*? The default is to re-apply the clustering algorithm after each new experiment is added to the modules. This allows the modules to evolve a little as the algorithm progresses. However to stop this, and only include experiments that match the clustering of the initial subset, change this to `FALSE`.
g. *Distance & linkage methods*. These are the methods that will be used to cluster genes. ModuleFinder uses R's `hclust` and `dist` functions for hierarchical clustering function. The available distance methods are "euclidean", "maximum", "manhattan", "canberra", "binary" or "minkowski". The available linkage methods are "". For more information about these, see the R help for the `dist` and `hclust` functions (type `help(dist)` or `help(hclust)` in R).
h. *Height & width of PDF file*. The main output of ModuleFinder is a PDF file including the parameter settings, heatmaps for each stage of clustering and, if you provide a column containing functional categories, pie charts representing the functional breakdown of each module. The default page

size for this file is A4, but you can specify its height and width (in inches) if you want to make it bigger. This may be useful if you find you are getting modules with more genes than can be comfortably displayed on an A4 page.

When the run has completed, you'll be asked if you want to run ModuleFinder again with the same data set. If you answer yes, the same expression data and p-values will be automatically loaded, and you will simply have to select a new starting set of experiments and change any parameters as you wish.

ModuleFinder Output

1. PDF file.

The major output file is a PDF file. The first page states the parameter settings. The subsequent pages provide heat maps with clustering trees for each stage of the process. If you specified functional categories in your data file, there will also be pie charts displaying the functional breakdown of each module into these categories. The last page contains a heat map displaying the expression of module genes in the experiments not included in the module.

2. Data files.

Expression data for the current subset of genes and experiments is saved at each stage as a comma-separated file (you can open these in Excel). The files are named according to the title that you provided at the start of the run, followed by "GeneClusters_Initial.csv" for the initial subset, and "GeneClusters_AddXXXX.csv" for each added experiment XXXX.
These files can also be imported straight into CoREG for promoter analysis

3. Cluster files (.cdt, .gtr) for viewing in TreeView and similar programs.

Cluster files are also saved at each stage. These files can be viewed directly from within TreeView and similar programs (TreeView can be downloaded from http://rana.lbl.gov/EisenSoftware.htm; Java TreeView, which I find particularly stable and flexible, can be downloaded from http://jtreeview.sourceforge.net). The files are named the same as the Data files, but with the extensions .cdt and .gtr.

CoREG Overview

What does CoREG do?
How does it work?

What CoREG does

CoREG aims to identify known and potential regulatory elements in the promoter regions of a set of genes, which explain the observed expression patterns of those genes. It is based on the hypothesis that changes in a gene's expression are the result of combinations of transcription factors binding at specific regulatory elements in the gene's promoter region. The TFs may be positive or negative regulators, thus the resulting changes in gene expression may be induction or repression. Furthermore, *CoREG* assumes that a similar fold change in the expression of two genes in a single microarray experiment can be explained by the binding of similar combinations of transcription factors to the genes' promoters. Finally it assumes there is a relationship between the degree of similarity in the fold changes and the degree of similarity in the combination of transcription factors binding within the promoters.

CoREG takes as its starting point the result of hierarchical clustering of a set of genes according to their expression fold changes during a set of treatments. This result is represented both by a clustering tree and the assignment of genes to discrete groups based on the clustering tree. That is, *CoREG* takes into account both the gene membership of each group and the relationship between the expression patterns of the groups that is characterised in the clustering tree. It then searches for a set of short sequence elements such that, when genes or groups are clustered hierarchically according to the occurrence of these elements in their promoter regions, the clustering tree has the same structure as that of the original clustering according to expression levels. It can then be proposed that the selected set of promoter elements captures the structure of the input gene expression patterns. Thus it can be hypothesised that the promoter elements correspond to regulatory elements that are responsible for these patterns of gene expression, and experiments designed to test this hypothesis in the laboratory.

How CoREG works

In addition to a hierarchical clustering of genes according to expression data and a corresponding assignment of the genes to discrete modules, *CoREG* requires a list of the genes' promoter sequences and a list of sequence elements to search for. The promoter sequences can be of any length and from any source such as TAIR or AGRIS. The list of sequence elements can also be from any source, although the set of all possible 6-base-pair sequences (hexamers) works nicely and doesn't require any prior knowledge of TF binding sites.

The first step is to generate an incidence table representing the occurrence of each element in each promoter sequence. This is simply a table with sequence elements on the horizontal axis, gene names on the vertical axis and values of TRUE or FALSE

indicating whether or not the element was found in a search of the gene's promoter sequence. Simple string matching is used to search for sequence elements in promoter sequences, thus only exact matches are recorded as TRUE. This differs from more complex pattern matching algorithms which can take into account substitutions, insertions and deletions when searching for motifs in DNA sequences.

The clustering tree is first broken down to define discrete groups of genes:

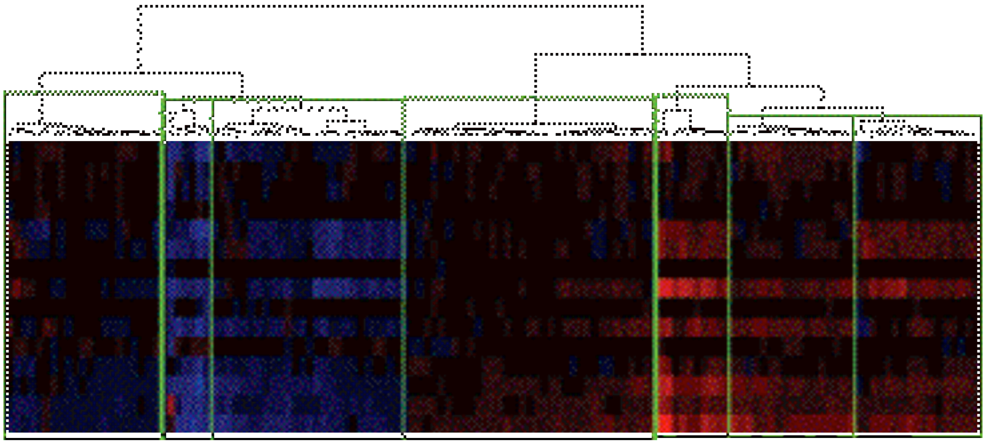

Next, the CoREG algorithm navigates down the tree, stopping at the first split into two branches, and searches for sequence elements whose frequency of occurrence varies widely between the two groups defined by the branches. For example, depending on the parameters set it can identify sequence elements that are present in the promoters of all the genes in one group but none in the other group, or in promoters of >80% of the genes in one group but <20% of the second group. The two branches resulting from the first split are then each broken down into two groups and sequence elements identified, and the process is repeated until the specified groups are reached. This process of breaking down the tree is illustrated below:

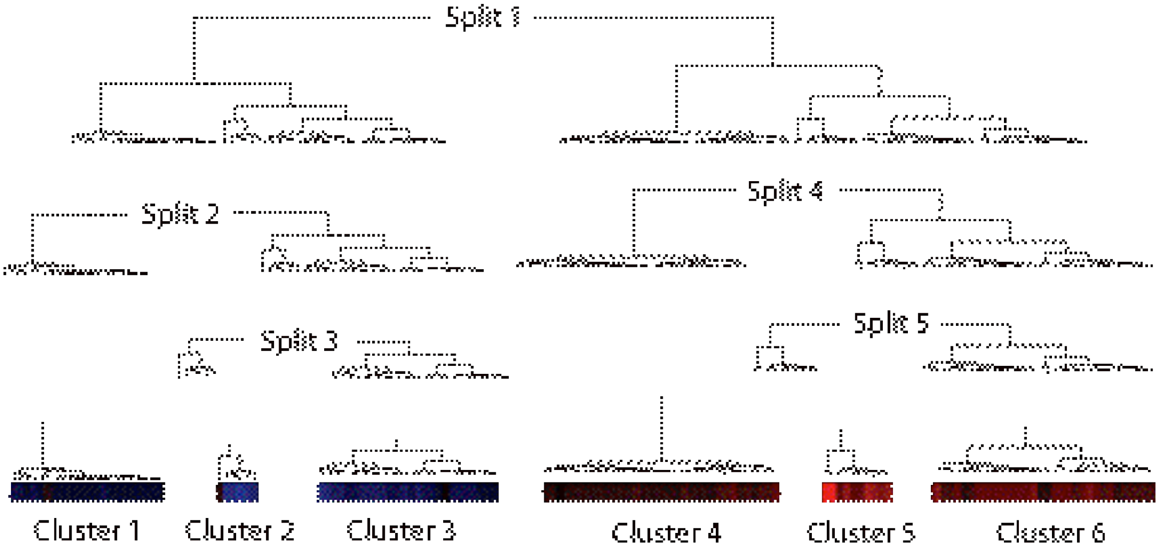

A frequency table is then calculated from the incidence table for this subset of sequence elements, representing the proportion of genes within each group that contain each sequence element. This is calculated using the gene to group assignments (e.g. genes A-F belong in group 1, genes G-M in group 2, etc) that are defined by the breaking down of the tree. This set of frequency data is then used to hierarchically cluster the groups. The resulting clustering tree is then compared to the expression-based clustering tree that was given as input. The goal of this step is to identify a group of sequence elements that cluster the modules into a similar hierarchy as the expression-based clustering. Thus if the clustering tree produced by the initial set of motifs doesn't achieve this, different subsets of sequence elements are trialled until the subset producing the closest matching tree is identified. The final clustering tree is displayed next to a heat map representation of the frequency table, in which squares are coloured according to a scale of white to black to represent sequence element frequencies in zero to 100% of the gene promoters in each group. This allows the occurrence of sequence elements within groups of genes to be visually compared with the expression patterns of the group, facilitating interpretation of results and formulation of models of gene regulation.

CoREG Tutorial

This tutorial will take you step-by-step through a run of CoREG on a Windows PC, using a subset of data from stress-related experiments from AtGenExpress. You will need to locate the two CSV files that came with CoREG – "MitoHexIncidenceTable.csv" and "OsmoSalt_DataSubset.csv". Note that if you have completed the ModuleFinder tutorial, the latter file will be identical to the data file described in Step 18 of that tutorial.

You should already have installed R and the necessary packages as outlined in the installation guide.

1. Create a new folder, call it something like 'CoREG Tutorial' and copy the two CSV data files into it.
2. Open R.
3. Set the current directory in R to the one you just created in step 1.

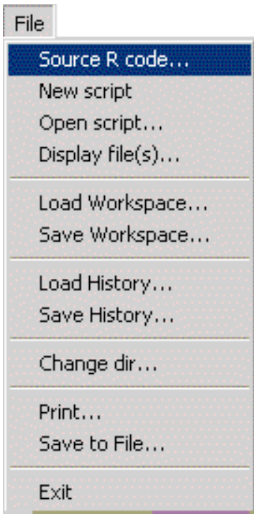

4. Load in the ModuleFinder R code, by going up to the file menu and selecting "Source R Code…"

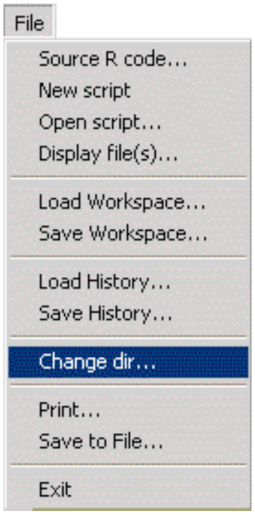

5. R will now load in the packages it requires. If you don't have necessary packages installed, do this now.
   (See the installation guide for details.)

6. A window will pop up, asking if you already have an incidence table or if you need to create one from a set of promoter sequences and a list of sequence elements. For this tutorial, you will use the incidence table "MitoHexIncidenceTable.csv". So click "Yes (Load incidence table now)", and double-click on the file name. Then click Finish.

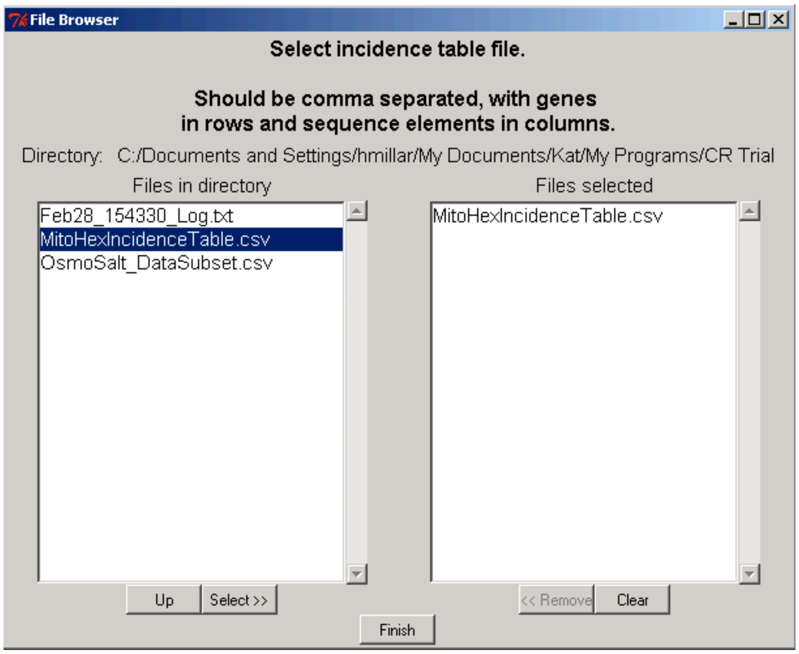

7. Next you will be asked to locate the expression data file. Double-click "OsmoSalt_DataSubset.csv" and click Finish.

8. Now you will be given the option to change the hierarchical clustering methods. Simply click “OK” to use the default settings.

9. An R image window will open, and a heatmap and clustering tree for the expression data will be drawn in it. Now, you need to specify how you want the tree broken down into clusters, by clicking on branches of the tree. Break the tree into 6 clusters, by clicking at the locations of the red crosses below. The boxes will be drawn around the resulting clusters as you go. When you have selected the 6 clusters, right-click in the image window and select “Stop”.

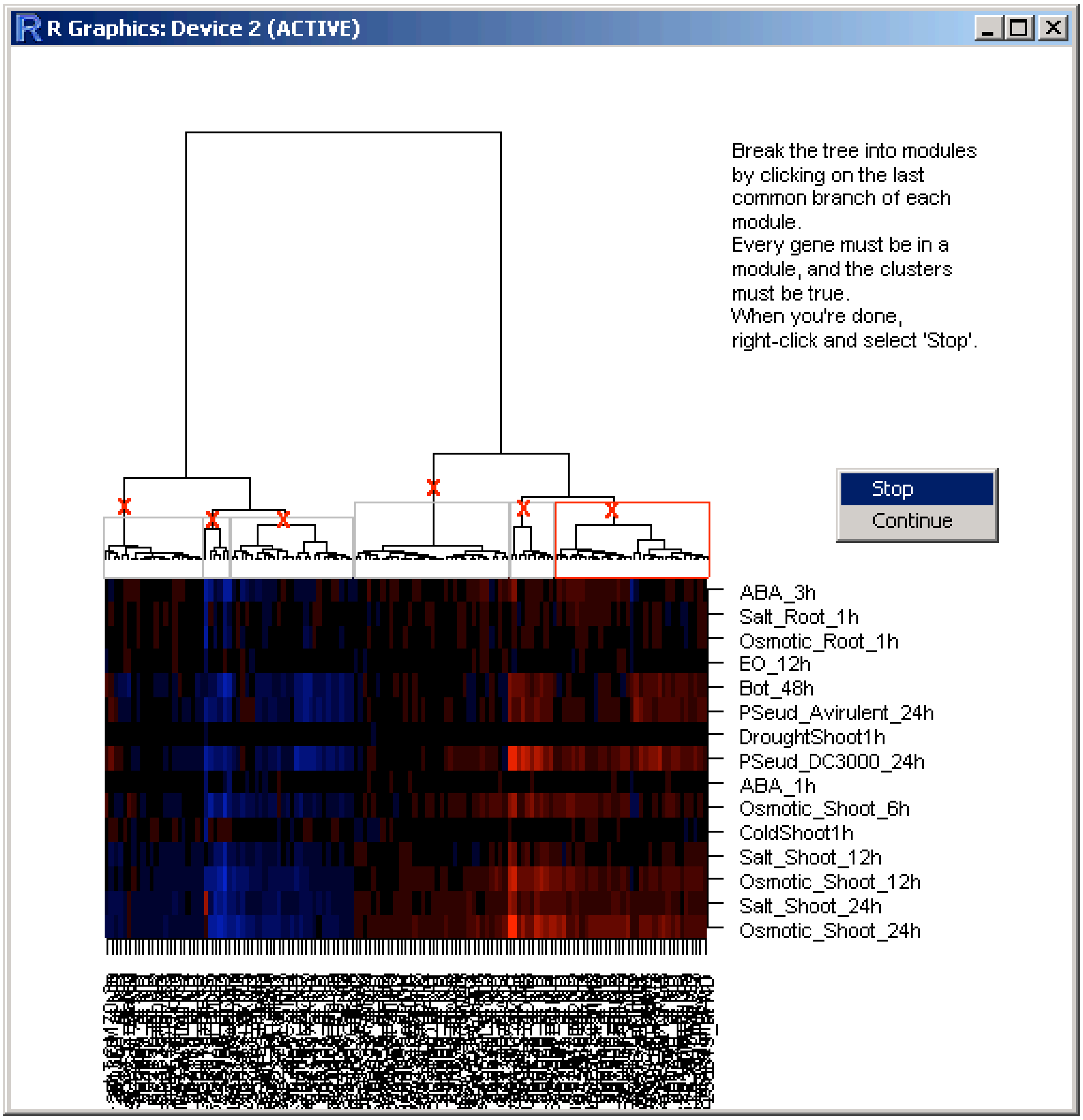

10. You will now be asked if you want to save the resulting clusters for viewing in MapMan. Answer “Yes” and enter a name for these files, e.g. “Tutorial”, so you can look at the output later.
11. Now you will be asked to set some parameters. You can either specify a minimum number of elements to find at each branching of the tree (a), or a frequency tolerance level (b). See the CoREG overview for more information. Here, delete the value in (a) so that the box is now empty. Then enter ‘0.35’ into the box at (b). This means that we will find any sequence elements whose frequency is <0.35 in one branch of the tree and >0.65 in the other.

    Leave the default value of 0.35 at (c). This means that CoREG will also identify any sequence elements with a frequency of <0.35 (or >0.65) in a single cluster but >0.65 (or <0.35) in all other clusters. This helps to identify elements that have a particularly high or low frequency in one cluster compared to all other clusters, and is thus ‘characteristic’ of that cluster. Click OK.

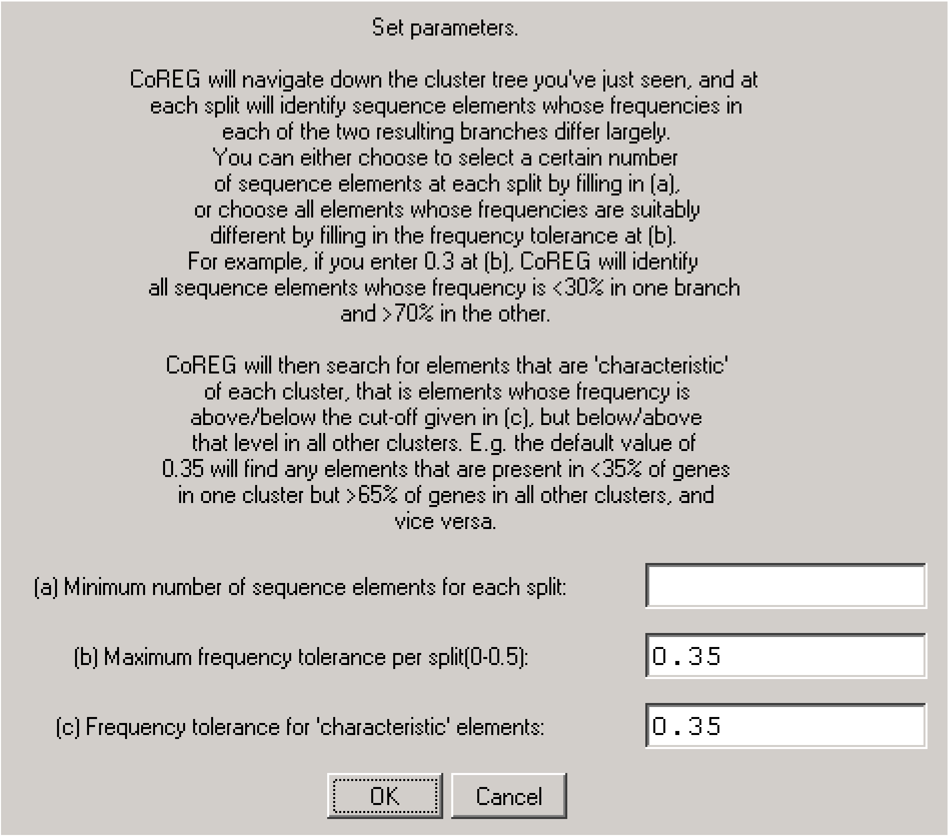

12. CoREG will now begin breaking down the tree into the 6 modules you specified, looking for sequence elements that have different frequencies in the branches created by each split. When it has finished, it will erase the heatmap in the image window and redraw the tree, this time truncated to create the six clusters. Next to this tree it will draw a frequency map for the 90 sequence elements that it identified. This represents the frequency of each of the found sequence elements in the promoters of module genes. Black = occurs in 100% of gene promoters, white = 0%, and shades of grey indicate intermediate values.

    There is also another clustering tree drawn, labeled “Tree Based on Element Frequencies”. This displays the result of clustering the modules using the frequencies of the 90 sequence elements. The structure of this tree is different to the one based on expression data, although there are similarities. Modules 1 and 3 are close together in both trees, as are modules 4 and 6. This suggests that the genes in modules 1 and 3 not only had similar expression levels in the subset of experiments we are looking at, but also share some sequence elements in their promoter regions. The next step will be to try to identify a subset of these elements which cluster the modules into the same structure as the first, expression based, tree.

    Save this image in whatever format you like by clicking on it and selecting “Save as…” from the File menu.

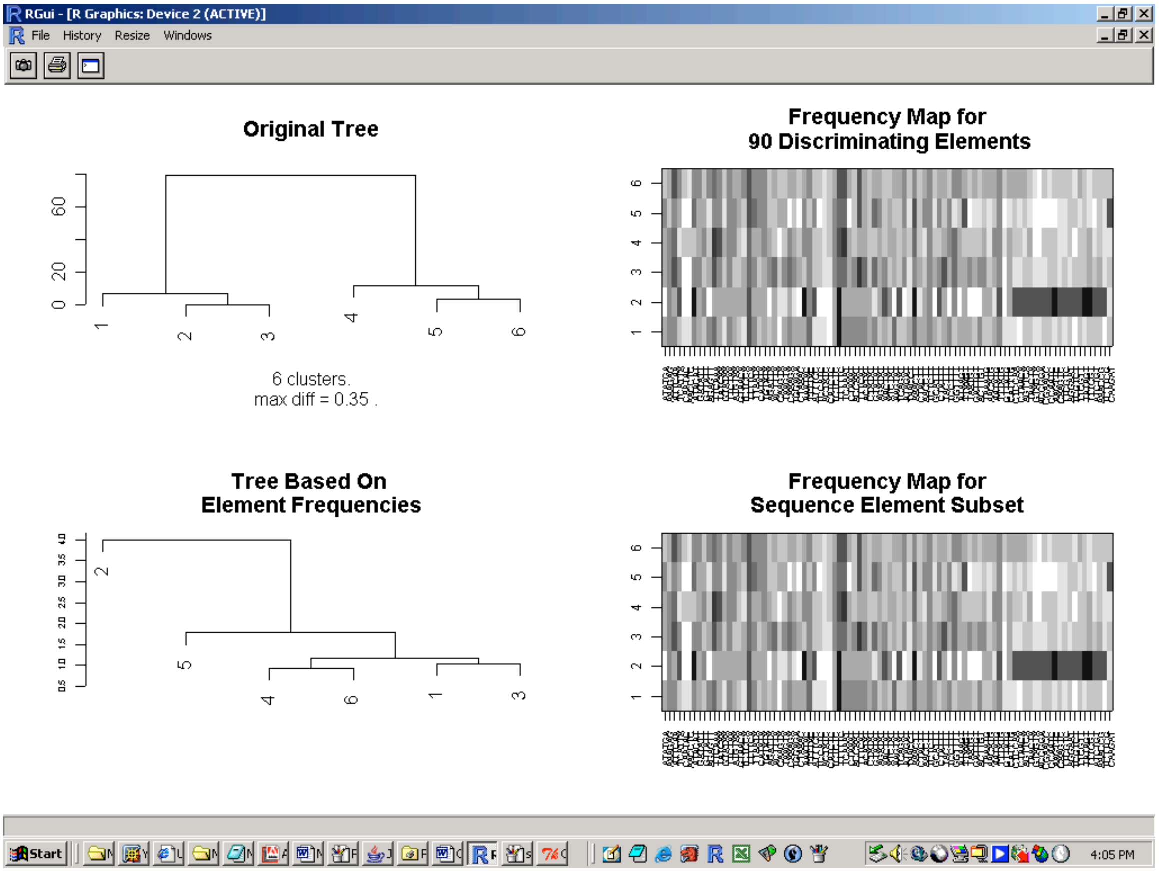

13. You will be asked if you want to change any parameters. This gives you the option to redefine the breakdown of the tree, and change the parameters that you entered first time around. This time answer No.

14. You will then be asked to provide a file name for saving the frequency data. This file will be an Excel-readable table containing a list of the 90 sequence elements, and their frequencies within each of the 6 modules. Call this whatever you like.

15. The next step is to try some different subsets of sequence elements. The aim here is to find a small set of elements which cluster the modules into a similar hierarchy as the expression-based tree. You can either let CoREG pick random subsets from the set of 90 sequence elements and see what you get, or you can look at the frequency map and try and pick out elements whose frequencies differ greatest between modules. Click “Yes (Choose a random subset for me)”. You might get a subset containing 5 elements or 70 elements, it is entirely random.

    The bottom half of the image will change, now displaying the frequency map for just the subset of sequence elements, and the tree that results from hierarchical clustering of the modules according to the frequencies of this subset of elements.

    Repeat this random subset selection a few times and see the different sorts of results you get. Note that you can save Excel-readable tables of frequencies for any of the subsets. You can also save the image at any time via the File menu.

16. You can also specify your own subsets of sequence elements. After you’ve clicked the random button a few times, click the other button “Yes (I will choose a new subset)”. Double-click on some of the elements on the left. Then click Finish.

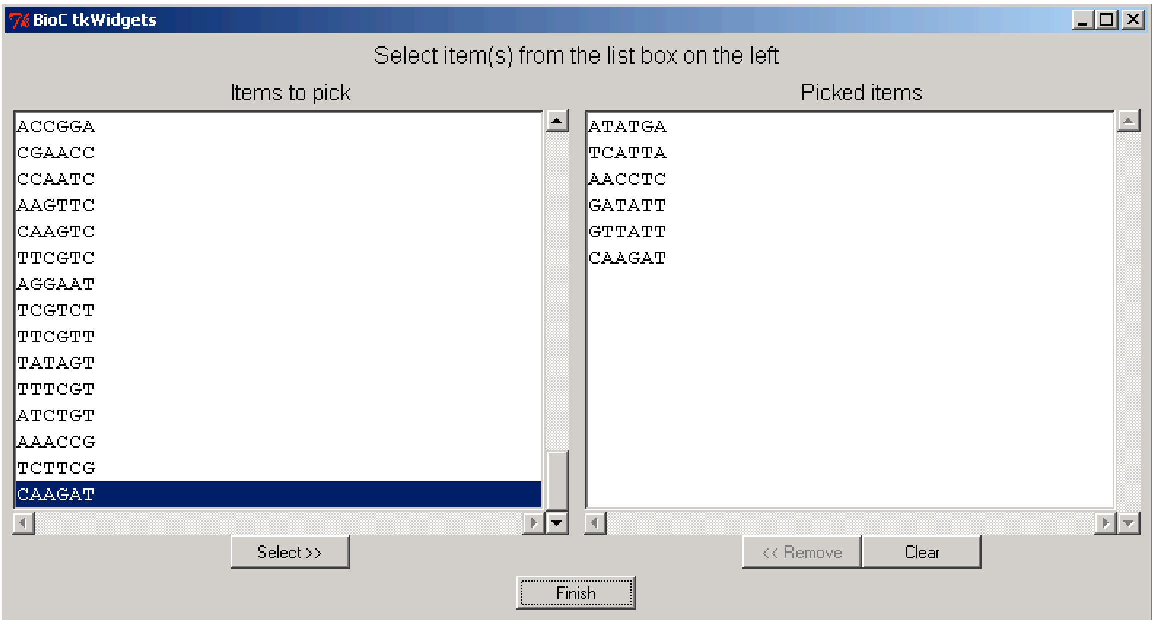

17. Try this a few more times and see if you can improve on the tree via your selection of sequence elements. Below is an example of a tree that looks a bit more similar to the expression-based tree, based on a subset of 8 sequence elements.

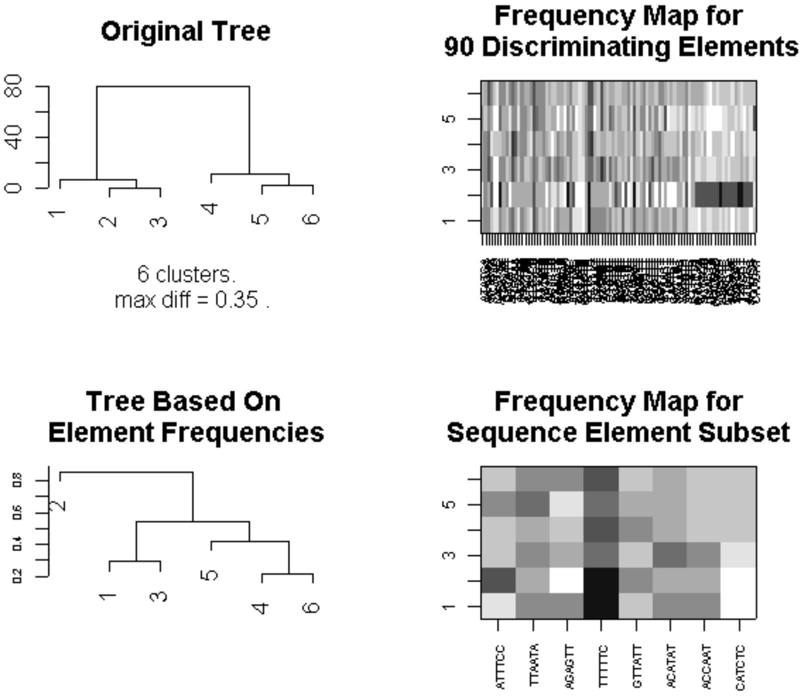

18. When you're happy with the results, click "No (Save current subset and close)", enter a name for the frequency table for your final selection, and save the image.

19. You can now have a look at some of the output files which will have appeared in the file you created in step 1. First have a look at the log file, which will be named according to the date and time you started the CoREG run. This will contain records of the incidence table and data file you loaded in at the start, the sequence elements that were identified, and the names of any files that were saved along the way. There will also be details of the MapMan files that were created. These will have names ending with "_Clusters_MapMan.txt" and "_Genes_MapMan.txt". View these files in MapMan now…

20. You will need to have the Mitochondria MapMan files that came with CoREG already loaded into MapMan. For details on how to do this, see the end of the "Using CoREG" guide. To view the files that CoREG created, open MapMan and double-click on the Mitochondria pathway. Then load in the newly created files. Select "add experiment" from the File menu, click "by name" and type in a name for the new folder, e.g. "CoREG trial". Then right-click on the new folder, and locate the file ending in "_Clusters_MapMan.txt". Click on the new file from within MapMan, and you will see coloured boxes appear on the mitochondria pathways picture. Select "Options" from the Pathway menu, and change the scale to above 7, say 7.5. Change the Datasize to L or XL so it's easier to see.

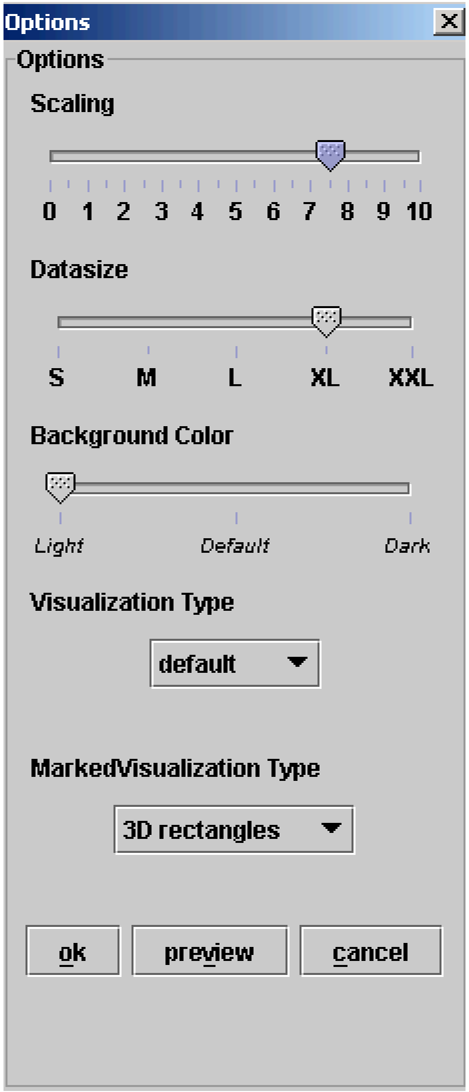

The boxes drawn on the pathway diagram correspond to genes that were among those in the data file you loaded into CoREG. Mouse-over a box to see precisely what that gene is.

The colour of the boxes indicates which of the osmotic stress-related modules the gene was clustered into. The boxes coloured dark blue (an AOX and an external NDH) denote genes that were in the most highly up-regulated module. Those coloured medium blue correspond to genes that were in the next highly up-regulated module (mainly TCA cycle genes in this example), and those coloured pale blue were in the slightly up-regulated module (e.g. 2 complex II genes, 2 defense genes). Similarly, dark red boxes indicate genes belonging to the most highly down-regulated module (e.g. a complex IV gene), bright red indicates the next down-regulated module (e.g. 2 TIM22 components) and pale red indicates the slightly down-regulated module (e.g. 3 protein synthesis genes).

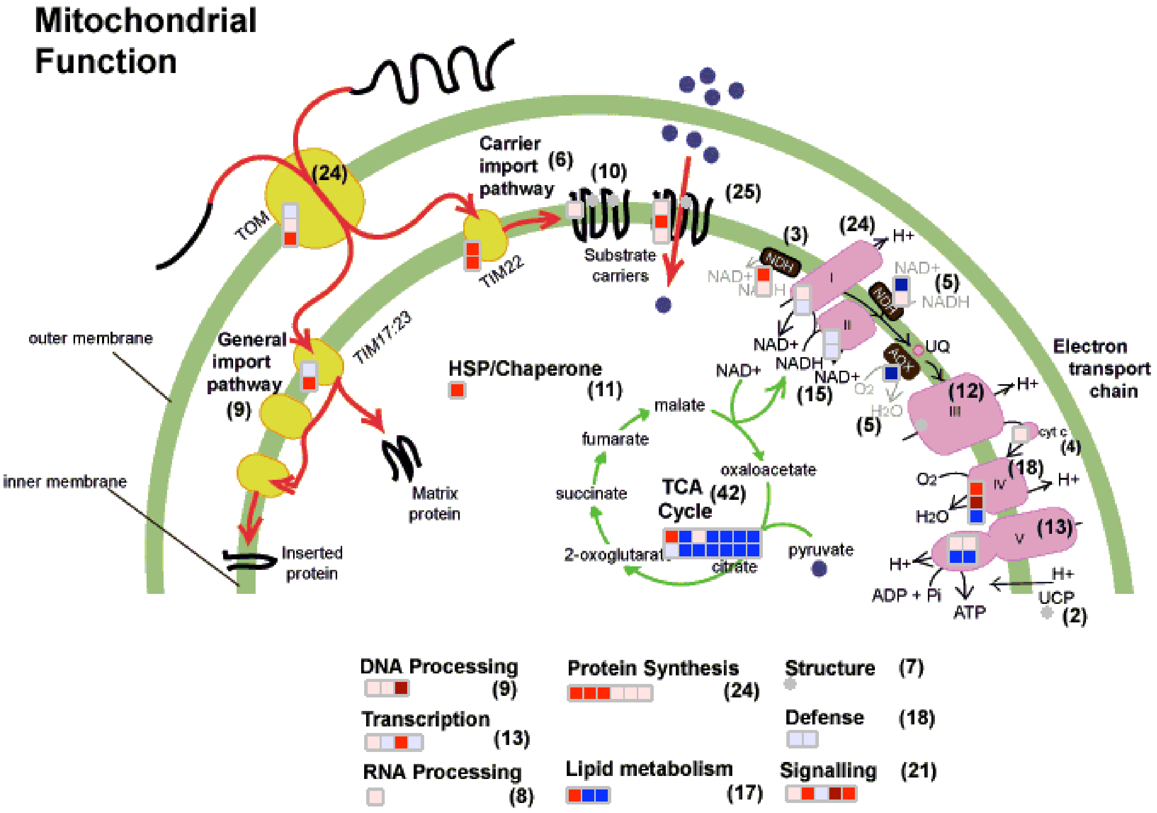

Using CoREG

This guide explains how to use and interpret CoREG.
For details on getting started, read the Installation Guide first.
For a general overview of how CoREG works and what it's for, see the CoREG Overview.

Preparation
Running CoREG
CoREG Output
CoREG and MapMan

Preparation

1. Make sure you have the necessary files ready.
   You will need to have at least two files to run ModuleFinder, which should all be in ***comma-separated format (.csv)*** (Excel can save files in this format):

   a. Incidence data. ***Either*** (1) a set of promoter sequences and a list of potential sequence elements to search for, ***or*** (2) an incidence table. The first time you use CoREG for a particular set of genes, you will need the promoter sequence for each gene and a list of sequence elements, from which an incidence table will be created. This table records the presence or absence of each of the sequence elements in each of the promoters, and will be automatically saved for future use. The next time you use CoREG with the same set of genes, you can simply load this incidence file directly.

      1. ***Promoter sequences & Sequence elements***
         a. The promoter sequences should be in a single file in FASTA format. NOTE: For Arabidopsis genes, these sequences can easily be obtained from TAIR's bulk sequence download facility (http://www.arabidopsis.org/tools/bulk/sequences/index.jsp). Simply paste in a list of locus identifiers, select "Upstream Sequences – 1000bp" as the dataset and FASTA as the output format. Then save the results as a text file – this will be your sequence file, and can be loaded directly into CoREG.
         b. You can either provide a list of sequence elements, or use the built-in set of all hexamers (the 4096 possible 6-bp sequences). If you want to provide your own list, it should be in the form of a comma-separated table, with the first column providing the nucleotide sequences themselves, and the second providing a name for each one. Note that the sequences must only consist of the base pairs (A/C/G/T) in uppercase; wildcards (N/R/Y/Y/etc) won't work. Also note that the more sequences you have, the longer it will take to create the incidence table

(which is quite computer-intensive), and the longer it will take to run CoREG.

2. ***Incidence table***. Whenever you load in promoter sequences and a list of sequence elements to create a new incidence table, this table will be saved to the current directory as a comma-separated file called "CoREG_IncidenceTable.csv". For subsequent runs of CoREG you can just load in this table directly. This is always a good idea since it can take quite a while to calculate a new incidence table every time.

b. File 2: ***Expression data*** for a subset of genes and experiments of interest.

   1. This should have genes in rows, the first column with locus identifiers and experimental data in the rest of the columns. The locus identifiers in the first column are used to lookup information in the incidence table, so should be precisely the same as the locus identifiers of the initial promoter sequences. This is case sensitive. If you follow the advice above about retrieving promoter sequences from TAIR, and make sure you label the expression data using the same locus identifiers (in uppercase), you should have no trouble.

   2. This file ***should not contain all your expression data***. This should be only be data for a subset of genes and experiments. Best results have been obtained with data on around 60 genes, in 10 experiments. The idea is that you use methods such as ModuleFinder to identify genes that respond in co-ordinated ways to a subset of experiments, and extract only the data for these genes and experiments. The output data files that ModuleFinder creates ("RunX_GeneClusters_AddY.csv") are designed to be loaded directly into CoREG, but you could source these data subsets via any method you choose.

2. Set the directory in which to save ModuleFinder output.
   a. This is done within R, by choosing "Change dir…" from the File menu.
   b. CoREG can create a lot of output files, so it is a good idea to create a new directory for this purpose. As with ModuleFinder, the best way to organize your use of CoREG is to create a new folder, and copy your promoter sequences, sequence elements, incidence tables, and expression data subsets into it. Then, set it as the current R directory. If you are using the Mac version you ***have to*** set the current directory to the one containing the input data files.

Running CoREG

1. To runCoREG with your data, in R select 'Source R code…' from the 'File' menu, and locate the file"CoREG.R" (GUI version for Windows) or "CoREGMAC.R" (Mac version).This will load all the CoREG functions into R, ready for using.

   You will then be asked for a series of inputs, including the location of your data files and some additional parameters.
   a. In the Windows version, this will be via dialogue boxes which allow you to make selections or enter information.
   b. In the Mac version this will be via a combination of dialogue boxes and the command line where questions will be printed, along with some guidance about how to answer them. To respond to these questions you will generally need to enter either a number, a name or a vector of these, then press RETURN.
      In R, all text must be surrounded by quotes to be recognized as such. (e.g. `"text"` or `"datafile.txt"`). Numbers can be entered as-is (i.e. `5` not `"5"`). Vectors in R are surrounded by brackets and preceded by a lowercase 'c', e.g. `c(1,2,3)` or `c("Exp1","Exp2")`.

2. You will first be asked whether you have an incidence table ready to load, or whether you need to create a new one from promoter sequences and a list of sequence elements. You will then be asked to locate the appropriate files.

   a. Next, you will be asked to locate the expression data file you wish to use.

   b. CoREG will now offer you the opportunity to change the hierarchical clustering methods it will use to cluster the input expression data.

   c. Then, CoREG will cluster the genes in the data file according to the expression data, and draw a heatmap and clustering tree in the R graphics window. (If you are using ModuleFinder output for your data files, this tree should look just like the ones in the corresponding ModuleFinder output, provided the clustering methods match.)

   Now you need to indicate how you want the tree broken down to form gene clusters. The rest of the CoREG run will be aimed at identifying sequence elements that can discriminate between these clusters; for more information on what you are doing at this step, see the CoREG overview.

      a. In the Mac version, you will simply be asked how many clusters to break the tree into.

      b. In the Windows version, you need to click on branches of the tree, to indicate the clusters you want. The clusters must be true,

meaning they must be able to be formed by truncating the entire tree at some height, and therefore each gene must be in a cluster.

c. When you click on the branches, red boxes are drawn around the resulting clusters. When the tree is appropriately broken down (remember every gene must be in a cluster, you can't leave bits of the tree out), right-click in the image window and select "Stop". If there was a problem with the way you broke down the tree, it will be redrawn and you can start again. Note that if you make an error, just right-click to stop and start again on the newly drawn tree.

d. When you've broken down the tree properly, you will be asked if you want to save a MapMan file of the clusters. If you want to create the file, say yes and enter a file name. In this file, each gene will be labeled with a number to indicate which cluster it belongs in. So, when you load the file into MapMan, the genes will be coloured according to cluster membership. You'll probably need to increase the scaling in MapMan to see the different colours (do this via the options menu). CoREG will also save a second file for viewing in MapMan, in which genes are labeled with their average expression across the set of experiments. So, when you load this file in MapMan, genes will be coloured according to their average expression level. You may need to change the scaling again to see the differential expression, depending on the expression levels in your particular dataset. For more details and information on what MapMan is and how to get it, see the CoREG & MapMan guide below.

e. Next you will be asked to set some parameters. You must set the minimum number of sequence elements to be found at each split of the tree. CoREG will return at least this many sequence elements for each split; if there are multiple elements at the same level, then more might be returned. Then, if you like you can set a maximum frequency tolerance, between 0 and 0.5. For example if you enter 0.3, CoREG will find all sequence elements that are present in at least 70% of one side of the split and no more than 30% of the other side.

f. The results of the search for sequence elements will be printed on the console as the search progresses. When the search is finished, if sequence elements were found, the results will be displayed graphically in the R image window. At this point you will be asked if you want to change any parameters – note this includes reselecting the clusters from the tree. If you aren't happy with the sequence elements that were found, answer yes and you'll be taken through steps 4 to 6 again.

NOTES ON THE IMAGES:
CoREG will have drawn four images in the image window. The one titled 'Original Tree' displays the earlier clustering tree truncated to form gene

expression clusters according to your earlier selections. The branches indicate the resulting set of clusters, labeled with a number. The clusters will be labeled with the same numbers in all subsequent output.
Next there is a frequency map for the discriminating elements that CoREG found. The numbers to the left indicate the gene expression clusters, and the labels on the bottom indicate sequence elements. The blocks are coloured to represent the frequencies of the sequence elements in the promoters of the cluster genes. White indicates a frequency of 0 (i.e. none of the genes in that expression cluster have that element in their promoters), black indicates a 100% frequency, and shades of grey indicate intermediate frequencies.
Below this is the frequency map for the current selection of sequence elements. At this point this will be the same as the first frequency map.
To the right of this is a hierarchical clustering tree displaying how the gene expression clusters are related according to the frequencies of the sequence elements. This can be thought of as the promoter-based tree. The idea is to try to get this tree to match the expression-based tree above it as closely as possible, by identifying the 'right' subset of sequence elements.

NOTE ON SAVING IMAGES:
The graphical output of CoREG will not be saved automatically. If at any time you want to save an image (i.e. whatever is displayed in the R image window), simply click on the image window and choose "Save as…" from the File menu. You can save R images as PDF files (which can then be edited in Adobe Illustrator) or as jpeg, bitmap, png, etc. It's a good idea to save the images of the results that you like, for later consideration.

g. When you are happy with the set of sequence elements that were found, CoREG will save the frequencies of the sequence elements in the chosen clusters, and ask for a name for this file. The file will be in comma-separated format, so you can open it in Excel later if you wish.

h. You will then be asked if you want to try different subsets of the sequence elements. The aim here is to find a subset of these elements that will result in a promoter-based tree (below) with a similar structure to the expression-based tree (above). You can try random subsets if you like, or you can select elements yourself. This step can be repeated as many times as you like, and you will always be able to save the frequencies of the current subset if you wish. And don't forget that you can save an image for the results you like at any time (File/Save as…).

When the run has completed, you'll be asked if you want to run CoREG again with the same incidence table. If you answer yes, the incidence table will be automatically loaded, and you will simply have to select a new expression data file and change any parameters as you wish.

CoREG Output Files

1. ***Log file***. CoREG creates a log file to record the progress of each CoREG run. These are text files, named with the date and time of the run. The file records:
    a. the incidence table, promoters and/or sequence elements that were used
    b. the input data file
    c. details of any MapMan output, including the titles of the files and a record of how clusters were labeled
    d. the parameter settings
    e. results of the search for sequence elements
    f. which sequence elements were used at each stage of clustering
    g. names of any files that are saved during the run
2. ***Frequency tables***. CoREG saves the frequency tables for every sequence element search, the final set of sequence elements, and any subsets you choose to save along the way. These are comma-separated files, named with whatever names you supplied during the run plus the ".csv" extension.
3. ***MapMan files (optional)***. CoREG can save files that contain records of the clusters that you select, which can be loaded into MapMan. See the guide below for details.

CoREG and MapMan

MapMan is a Java program that allows you to annotate images with data from a text file (Download from http://gabi.rzpd.de/projects/MapMan/). Images can be loaded into the program that represent pathways of genes, then mapping and files are loaded which assign gene identifiers into functional categories. Then pathway images can be annotated, which involves mapping gene identifiers to particular positions on the image. Then you can load in a file containing a list of gene identifiers with values assigned to each (e.g. an expression value from a particular experiment). The genes in the file then appear as dots in the appropriate positions on the pathway image, coloured according to the value (e.g. expression level) in the loaded file. This allows you to more easily identify if a number of genes in a pathway were induced or repressed in an experiment. For example, the mapping below shows 6 clusters of Arabidopsis mitochondrial genes mapped to an image depicting mitochondrial functions. The genes coloured blue were in clusters that were up-regulated in response to a set of abiotic treatments; those in red belong to down-regulated clusters. The darker the colour, the greater the average fold change in expression of the cluster. Thus it is easy to spot that there's a cluster of up-regulated TCA cycle genes (medium blue), a cluster of down-regulated protein fate genes (medium red – components of import machinery, heat shock proteins, protein synthesis), and a cluster of highly up-regulated genes including an alternative oxidase and an external class alternative NADH dehydrogenase (dark blue), which together encode an a bypass of the mitochondrial respiratory chain.

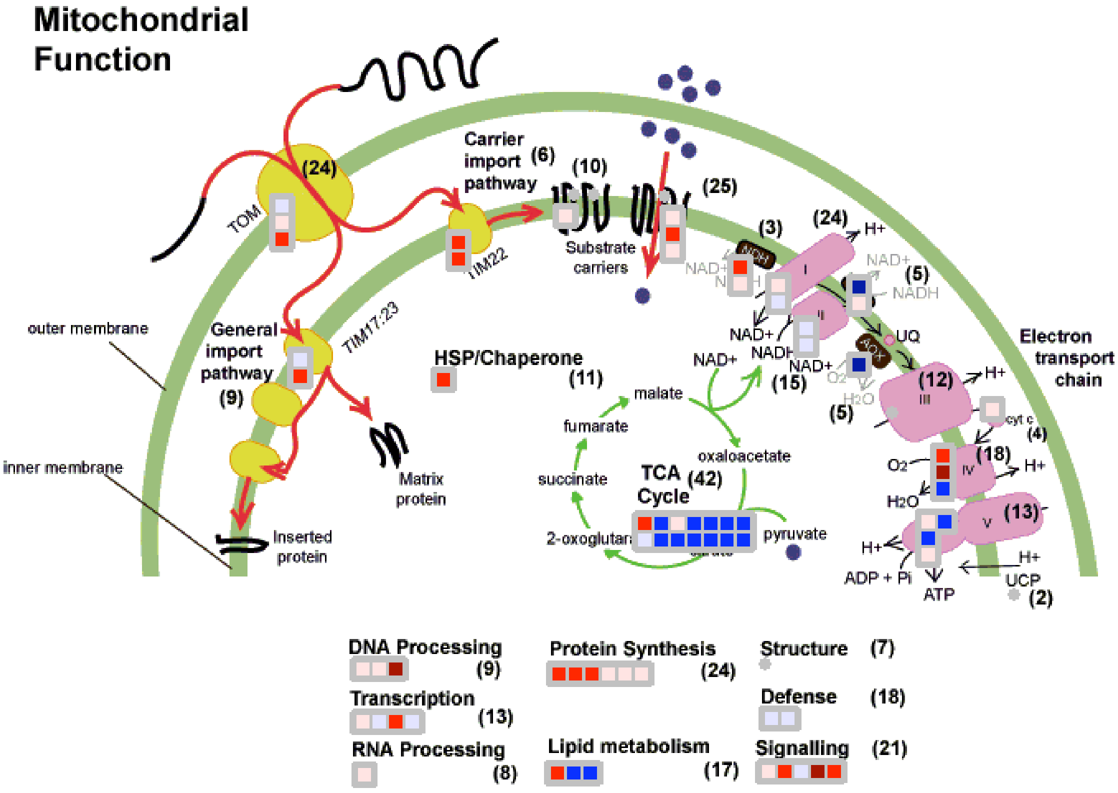

A number of mappings and annotated images come with the standard MapMan download, but you can add your own if you like. The one shown above is included in the ModuleFinder/CoREG zip file. To load it for use with your own data, you need to:

1. Open MapMan.
2. Choose "add mapping" from the file menu, click "from file", and locate the file "MitochondriaMapping.xls".
3. Choose "add pathway" from the file menu, click "from file", and locate the file "Mitochondria.bmp".
4. A dialogue box should appear, asking you to choose a mapping for this pathway. Select "MitochondriaMapping" and click OK.
5. You should now be able to load in data including Arabidopsis genes targeted to the mitochondria, and visualize them on these pathways. See MapMan help for details. Note that the gene identifiers in the mapping file are Agi loci with uppercase letters, so you will need to use the same identifiers in the data files you want to view.
