# Supplementary material for: ModuleFinder and CoReg: alternative tools for linking gene expression modules with promoter sequences motifs to uncover gene regulation mechanisms in plants
Source: Plant Methods. 2006 Apr 11;2:8. doi: 10.1186/1746-4811-2-8 (PMC1479336; doi:10.1186/1746-4811-2-8)
Supplement: Additional File 6 — User guide (htm files).zip Instruction for use in htm format [file 1746-4811-2-8-S6.zip › User guide(htm files)/AboutCR.htm]

CoREG Overview


**CoREG Overview**

***What does CoREG do?***

***How does it work?***

 

## What CoREG does

*CoREG*
aims to identify known and potential regulatory elements in the promoter
regions of a set of genes, which explain the observed expression patterns of
those genes. It is based on the hypothesis that changes in a gene�s expression
are the result of combinations of transcription factors binding at specific regulatory
elements in the gene�s promoter region. The TFs may be positive or negative
regulators, thus the resulting changes in gene expression may be induction or
repression. Furthermore, *CoREG* assumes that a similar fold change in the
expression of two genes in a single microarray experiment can be explained by
the binding of similar combinations of transcription factors to the genes� promoters.
Finally it assumes there is a relationship between the degree of similarity in
the fold changes and the degree of similarity in the combination of
transcription factors binding within the promoters.

*CoREG*
takes as its starting point the result of hierarchical clustering of a set of
genes according to their expression fold changes during a set of treatments.
This result is represented both by a clustering tree and the assignment of
genes to discrete groups based on the clustering tree. That is, *CoREG* takes
into account both the gene membership of each group and the relationship
between the expression patterns of the groups that is characterised in the
clustering tree. It then searches for a set of short sequence elements such
that, when genes or groups are clustered hierarchically according to the
occurrence of these elements in their promoter regions, the clustering tree has
the same structure as that of the original clustering according to expression
levels. It can then be proposed that the selected set of promoter elements captures
the structure of the input gene expression patterns.  Thus it can be hypothesised that the
promoter elements correspond to regulatory elements that are responsible for
these patterns of gene expression, and experiments designed to test this
hypothesis in the laboratory.

 

## How CoREG works

In addition to a
hierarchical clustering of genes according to expression data and a
corresponding assignment of the genes to discrete modules, *CoREG*
requires a list of the genes� promoter sequences and a list of sequence
elements to search for. The promoter sequences can be of any length and from
any source such as TAIR or AGRIS. The list of sequence elements can also be
from any source, although the set of all possible 6-base-pairsequences
(hexamers) works nicely and doesn�t require any prior knowledge of TF binding
sites.

 

The first step
is to generate an incidence table representing the occurrence of each element
in each promoter sequence. This is simply a table with sequence elements on the
horizontal axis, gene names on the vertical axis and values of TRUE or FALSE
indicating whether or not the element was found in a search of the gene�s
promoter sequence. Simple string matching is used to search for sequence
elements in promoter sequences, thus only exact matches are recorded as TRUE.
This differs from more complex pattern matching algorithms which can take into
account substitutions, insertions and deletions when searching for motifs in DNA
sequences.

 

The clustering
tree is first broken down to define discrete groups of genes:

Next, the CoREG algorithm
navigates down the tree, stopping at the first split into two branches, and
searches for sequence elements whose frequency of occurrence varies widely
between the two groups defined by the branches. For example, depending on the
parameters set it can identify sequence elements that are present in the
promoters of all the genes in one group but none in the other group, or in
promoters of >80% of the genes in one group but <20% of the second group.
The two branches resulting from the first split are then each broken down into
two groups and sequence elements identified, and the process is repeated until
the specified groups are reached. This process of breaking down the tree is
illustrated below:

A frequency
table is then calculated from the incidence table for this subset of sequence
elements, representing the proportion of genes within each group that contain
each sequence element. This is calculated using the gene to group assignments (e.g.
genes A-F belong in group 1, genes G-M in group 2, etc) that are defined by the
breaking down of the tree. This set of frequency data is then used to
hierarchically cluster the groups. The resulting clustering tree is then
compared to the expression-based clustering tree that was given as input. The
goal of this step is to identify a group of sequence elements that cluster the
modules into a similar hierarchy as the expression-based clustering. Thus if
the clustering tree produced by the initial set of motifs doesn�t achieve this,
different subsets of sequence elements are trialled until the subset producing
the closest matching tree is identified. The final clustering tree is displayed
next to a heat map representation of the frequency table, in which squares are coloured
according to a scale of white to black to represent sequence element frequencies
in zero to 100% of the gene promoters in each group. This allows the occurrence
of sequence elements within groups of genes to be visually compared with the
expression patterns of the group, facilitating interpretation of results and formulation
of models of gene regulation.

 

**ModuleFinder&
CoREG**
