# Supplementary material for: ModuleFinder and CoReg: alternative tools for linking gene expression modules with promoter sequences motifs to uncover gene regulation mechanisms in plants
Source: Plant Methods. 2006 Apr 11;2:8. doi: 10.1186/1746-4811-2-8 (PMC1479336; doi:10.1186/1746-4811-2-8)
Supplement: Additional File 6 — User guide (htm files).zip Instruction for use in htm format [file 1746-4811-2-8-S6.zip › User guide(htm files)/AboutMF.htm]

In order to better characterise the transcriptional responses of
variouspathways under a variety of conditions, it is desirabl


**ModuleFinder Overview**

***Why ModuleFinder?***

***What are the benefits?***

***How does it work?***

**Overview**

**Schema**

**Specifics**

# Why was ModuleFinder created?

In order to
better characterise the transcriptional responses of various pathways under a
variety of conditions, it is desirable to define subsets of genes whose
expression is co-ordinated during the response to particular subsets of
conditions. In this way, the relationships between conditions can be linked to
the co-ordinated expression of subsets of genes, giving a much clearer picture
of the mechanisms of responses to various conditions at the level of gene
expression. It is also desirable to be able to ask specific questions about any
similarity in expression responses to a subset of conditions of interest, for
example, which genes are regulated in the same way in response to two different
sorts of abiotic stresses. To accomplish these aims, there is a need for a
method of analysis of gene expression data that allows experiments and genes to
be analysed together in an integrated way, capable of identifying subsets of
both genes and experiments under which gene expression is co-ordinated, despite
a lack of overall co-ordination across the entire data set. The resulting
subsets of genes would thus constitute gene expression modules specific to
particular subsets of conditions, which would arguably be more easily
interpretable and more biologically significant than gene clusters derived from
simple clustering methods across a broader set of experiments.

 

# What are the benefits?

1. Shared responses can
be lost in simple cluster analysis

Cluster
analysis is a useful technique for identifying genes whose expression patterns
across a given set of stresses are similar. For example, the analysis will
cluster together all those genes whose expression is upregulated in response to
conditions A, B and C, downregulated in response to conditions D, E and F, and
unaffected by conditions G and H. However since expression data from *all*
experiments is used in the analysis, this cluster won�t include genes that are
upregulated in response to A, B, C and G, and downregulated in response to D,
E, F and H. These will be grouped together into a separate cluster since their
expression patterns differ under stresses G and H. The similarity between the
clusters in response to stresses A to F is lost in the analysis. Yet from a
biological point of view, the fact that both clusters display co-ordinated
expression in response to stresses A to F is very interesting. It may indicate
that they are co-regulated by a factor that is induced or activated under stresses
A-C and repressed or inactivated under stresses D-F. Thus it would be
informative to identify both clusters of genes, and the stresses A-F, as a gene
expression module. Such a module is more likely to have biological significance
than the two separate groups produced by cluster analysis.

 

# 

# | | | --- | | | | | | Shared gene expression responses can be split up in simple cluster analysis

A) Cluster analysis groups together genes whose
expression patterns are similar across all available experiments. Thus cluster
analysis of genes 1 to 12 in treatments A to H above, splits the genes into the
three separate clusters shown in the figure. The splitting of genes 1-4 and9-12
into separate clusters is due only to their differential expression in
experiments G and H. The shared expression response of Cluster 1 and 3 genes,
very interesting from a biological point of view, is lost in this analysis.

B) Clusters 1 and 3 (genes 1-4, 9-12) are
co-ordinately expressed in response to treatments A-F, thus this combination of
genes and treatments can be identified as a single gene expression module in an
alternative method of analysis.

 

2. Existing
approaches are inadequate for identifying shared responses among numerous
non-linear-related sets of conditions

The majority
of methods that involve analysis of both genes and samples from microarray data
are aimed at identifying genes that can discriminate between two or three
groups of samples. These are generally driven by the needs of cancer
researchers, who wish to identify genes that can be used in diagnostic or
prognostic tests in cancer patients. Unfortunately these methods are not
appropriate for analysing sets of conditions that do not fall into discrete
categories which aren�t related in any linear way such as a time course or
multiple dilutions of one or two treatments. In this case, to identify gene
expression patterns that are shared among subsets of the conditions requires
the analysis of genes and samples to be integrated in a way that doesn�t depend
on defined relationships between the samples.

A
handful of techniques have been developed to achieve this, including coupled
two-way clustering (*CTWC*) and conserved gene expression motifs (*XMotif*).
*CTWC* works by clustering genes into subsets, then clustering samples
into subsets. Each gene subset-sample subset pair is then considered as a
submatrix and genes and samples are re-clustered within that submatrix (Getz et al., 2000). The result is a collection
of subsets of genes and samples (gene expression modules), which theoretically
should display co-ordinated expression patterns. However, this fragmentation of
the data into small discrete modules makes it difficult to interpret the
results and particularly difficult to see overall trends in the expression
patterns. *XMotif* also attempts to identify subsets of genes and samples
in which the expression of the genes is co-ordinated, but using a much more
complicated algorithm (Murali and Kasif, 2003). The computer code for this
algorithm was not available for this study but, like *CTWC*, this method
is liable to produce a large set of fragmented modules that is difficult to
interpret. ModuleFinder was developed, with the aim of identifying gene
expression modules in a way that facilitates interpretation of results. It also
needed to allow easy visualisation not only of the expression patterns of
discrete modules, but also of the relationships between the modules.

 

# How does it work?

## 1. General procedure

*a)
Input*

The
algorithm takes as its input a matrix of summarised expression data from a set of
experiments. The data should be average log ratios from replicate experimental
and control samples. It also requires a matrix of p-values associated with each
data point, providing an estimate of how likely the observed gene expression
values would be if there was really no change in experimental compared to
control conditions. These must be calculated from the original microarray data
using t-tests or similar, and loaded into ModuleFinder.

*b)
Processing*

The
algorithm begins with a subset of experiments and extracts the genes whose
expression changed in those experiments. It then clusters the genes and splits
them into co-expressed modules. Next the algorithm searches for another
experiment (outside the initial subset) which fits the expression patterns of
these modules. The new experiment is added to the module and the genes arere-clustered.
Experiments are added one by one in an iterative procedure of searching for
matching experiments and re-clustering the genes, until no more can be found
that fit the module expression patterns. The resulting subsets of genes and
experiments are referred to as gene expression modules, as they define not only
gene clusters but subsets of genes whose expression is co-ordinated under a
subset of experiments. The algorithm is thus named ModuleFinder.

*c)
Output*

The
output of the algorithm is a PDF file containing clustering trees and
expression heat maps produced after the addition of each new experiment. It
also includes pie charts displaying the breakdown of each module according to
the functional categories of its member genes. Figure 3.2 provides a graphical
representation of the algorithm.

## 2. Schema

|  |
| --- |
|  |
|  |  |

 

## 3. Specifics

*a)
Initial phase: identifying gene expression response patterns shared by an
initial subset of* *experiments
(See figure A above)*

1.  The algorithm begins with a subset of one or more experiments, which
can be user-defined or selected by the algorithm. If no subset is defined by
the user, the algorithm will select the pair of experiments that are most
highly correlated across the whole gene set.

2.  The algorithm filters out the genes whose expression did not change
under all experiments in the subset. This is done by considering the matrix of p-values
provided by the user, and filtering out all genes whose p-values are above a
set cut-off in any of the experiments in the subset. The default p-value
cut-off is 0.05, but can be set by the user to any value between 0 and1.

3.     
The remaining genes, whose expression changed under all
experiments in the subset, are clustered based on their expression levels in
those experiments. The default clustering method uses a Euclidean distance
measure and the complete linkage method, but can be set by the user to any of
the hierarchical clustering methods available in *R*.(These include
Minkowski, Canberra, maximum, minimum and Manhattan distances, and the
complete, single, average, centroid, Ward and McQuitty methods of linkage.)

4.     
The resulting clustering tree is used to split the
genes into a given number of clusters. The number of clusters can be
user-defined, or chosen by the algorithm so that the number of clusters is
closest to the average number of genes in each cluster.

5.     
The clustering tree and corresponding expression heat
map is drawn in the PDF output file. In the figures produced, each gene is
labelled by the module it appears in, its functional category, its Agi locus
and a descriptive name.

6.     
The combination of each gene cluster and the experiment
subset produces a gene expression module. For each module, the algorithm draws
in the PDF file a pie chart representing the functional breakdown of its member
genes. Below the pie charts, expression heat maps for the corresponding
module are displayed.

 

*b)
Iterative phase: identifying further experiments that share the identified gene
expression response patterns* *(See figure B above)*

7.     
Having defined modules containing genes that are
co-ordinately expressed in response to a subset of experiments, the algorithm
searches for further experiments in which these modules also display
co-ordinated expression responses. For each experiment not already in the
module, the variance of the gene expression measures within each module is
calculated. A small within-module variance can be interpreted as a high level
of co-expression among the genes in the module. The sum of these within-module
variance measures is calculated as an overall measure of how well gene
expression in the experiment fits the set of modules. A measure of
between-module variance is also calculated for each experiment. Large values
here indicate that the modules had distinct expression patterns in the
experiment. The experiment that most closely �fits� the module structure will
display co-ordinated gene expression within modules and, ideally, distinct
patterns of gene expression between modules. That is, it will have small within-module
variances and a large between-module variance. The algorithm thus looks for the
experiment with the highest ratio of between-module variance to sum of
within-module variances.

8.     
The clustering tree already defined is redrawn in the
PDF file, beside a heat map including expression data from the new experiment.

9.     
The genes are then re-clustered based on expression
data from the new experiment swell as the initial experiment subset. The same
clustering method is used in the initial and iterative phases.

10.  The
new clustering tree is then used to split the genes into a given number of
clusters. The number of clusters is the same in the initial and iterative
phases.

11.  The
new clustering tree and corresponding expression heat map is drawn in the PDF
file.

12.  Pie
charts representing the functional breakdowns of the new modules are drawn in
the PDF file.

Steps7
to 12 are repeated until no more experiments can be found that have a
sufficiently high ratio of between-module to within-module variance. The
default cut-off value for the ratio is 4, but can be user-defined.

 

**Getz, G., Levine, E.,
and Domany, E.** (2000). Coupled two-way clustering
analysis of gene microarray data. Proc Natl Acad Sci
U S A **97,** 12079-12084.

**Murali, T.M., and Kasif, S.**
(2003). Extracting conserved gene expression motifs from gene expression data. Pac Symp Biocomput**,**77-88.

 

 

**Links**

Installation

ModuleFinder Tutorial

Using ModuleFinder

ModuleFinder&
CoREG
