# Supplementary material for: ModuleFinder and CoReg: alternative tools for linking gene expression modules with promoter sequences motifs to uncover gene regulation mechanisms in plants
Source: Plant Methods. 2006 Apr 11;2:8. doi: 10.1186/1746-4811-2-8 (PMC1479336; doi:10.1186/1746-4811-2-8)
Supplement: Additional File 6 — User guide (htm files).zip Instruction for use in htm format [file 1746-4811-2-8-S6.zip › User guide(htm files)/CRTut.htm]

CoREG Tutorial


# CoREG Tutorial

PDF version

 

This
tutorial will take you step-by-step through a run of CoREG on a Windows PC,
using a subset of data from stress-related experiments from AtGenExpress. You
will need to locate the two CSV files that came with CoREG Ð
ÒMitoHexIncidenceTable.csvÓ and ÒOsmoSalt\_DataSubset.csvÓ.
Note that if you have completed the ModuleFinder tutorial, the latter file will
be identical to the data file described in Step 18 of that tutorial.

 

You should
already have installed R and the necessary packages as outlined in the
installation guide.

 

1. Create a new folder, call it
   something like ÔCoREG TutorialÕ and copy the two CSV data files into it.
2. Open R.
3. Set the current directory in R
   to the one you just created in step 1.

 

4. Load in the ModuleFinder R
   code, by going up to the file menu and selecting ÒSource R CodeÉÓ

  

5. R will now load
   in the packages it requires. If you don't have necessary packages
   installed, do this now.  
   (See the installation guide for details.)

 

6. A window will pop up, asking if
   you already have an incidence table or if you need to create one from a
   set of promoter sequences and a list of sequence elements. For this
   tutorial, you will use the incidence table ÒMitoHexIncidenceTable.csvÓ. So
   click ÒYes (Load incidence table now)Ó, and double-click on the file name.
   Then click Finish.

7. Next you will be asked to
   locate the expression data file. Double-click ÒOsmoSalt\_DataSubset.csvÓ
   and click Finish.

 

8. Now you will be given the
   option to change the hierarchical clustering methods. Simply click ÒOKÓ to
   use the default settings.

 

9. An R image window will open,
   and a heatmap and clustering tree for the expression data will be drawn in
   it. Now, you need to specify how you want the tree broken down into
   clusters, by clicking on branches of the tree. Break the tree into 6
   clusters, by clicking at the locations of the red crosses below. The boxes
   will be drawn around the resulting clusters as you go. When you have
   selected the 6 clusters, right-click in the image window and select
   ÒStopÓ.

 

 

10. You will now be asked if you
    want to save the resulting clusters for viewing in MapMan. Answer ÒYesÓ
    and enter a name for these files, e.g. ÒTutorialÓ, so you can look at the
    output later.
11. Now you will be asked to set
    some parameters. You can either specify a minimum number of elements to
    find at each branching of the tree (a), or a frequency tolerance level
    (b). See the CoREG overview for more information. Here, delete the value
    in (a) so that the box is now empty. Then enter Ô0.35Õ into the box at (b).
    This means that we will find any sequence elements whose frequency is
    <0.35 in one branch of the tree and >0.65 in the other.

 

Leave the default value of 0.35 at (c). This
means that CoREG will also identify any sequence elements with a frequency of
<0.35 (or >0.65) in a single cluster but >0.65 (or <0.35) in all
other clusters. This helps to identify elements that have a particularly high
or low frequency in one cluster compared to all other clusters, and is thus
ÔcharacteristicÕ of that cluster. Click OK.

 

 

  

12. CoREG will now
    begin breaking down the tree into the 6 modules you specified, looking for
    sequence elements that have different frequencies in the branches created
    by each split. When it has finished, it will erase the heatmap in the
    image window and redraw the tree, this time truncated to create the six
    clusters. Next to this tree it will draw a frequency map for the 90
    sequence elements that it identified. This represents the frequency of
    each of the found sequence elements in the promoters of module genes.
    Black = occurs in 100% of gene promoters, white = 0%, and shades of grey
    indicate intermediate values.

 

There is also another clustering tree drawn,
labeled ÒTree Based on Element FrequenciesÓ. This displays the result of
clustering the modules using the frequencies of the90 sequence elements. The
structure of this tree is different to the one based on expression data,
although there are similarities. Modules 1 and 3 are close together in both
trees, as are modules 4 and 6. This suggests that the genes in modules 1 and 3
not only had similar expression levels in the subset of experiments we are
looking at, but also share some sequence elements in their promoter regions.
The next step will be to try to identify a subset of these elements which
cluster the modules into the same structure as the first, expression based,
tree.

 

Save this image in whatever format you like by
clicking on it and selecting ÒSave asÉÓ from the File menu.

 

 

13. You will be asked if you want
    to change any parameters. This gives you the option to redefine the
    breakdown of the tree, and change the parameters that you entered first
    time around. This time answer No.

 

14. You will then be asked to
    provide a file name for saving the frequency data. This file will be an
    Excel-readable table containing a list of the 90 sequence elements, and
    their frequencies within each of the 6 modules. Call this whatever you
    like.

 

15. The next step is to try some
    different subsets of sequence elements. The aim here is to find a small
    set of elements which cluster the modules into a similar hierarchy as the
    expression-based tree. You can either let CoREG pick random subsets from
    the set of 90 sequence elements and see what you get, or you can look at
    the frequency map and try and pick out elements whose frequencies differ greatest
    between modules. Click ÒYes (Choose a random subset for me)Ó. You might
    get a subset containing 5 elements or 70 elements, it is entirely random.

 

The bottom half of the image will change, now
displaying the frequency map for just the subset of sequence elements, and the
tree that results from hierarchical clustering of the modules according to the
frequencies of this subset of elements.

 

Repeat this random subset selection a few times
and see the different sorts of results you get. Note that you can save
Excel-readable tables of frequencies for any of the subsets. You can also save
the image at any time via the File menu.

 

16. You can also specify your own
    subsets of sequence elements. After youÕve clicked the random button a few
    times, click the other button ÒYes (I will choose a new subset)Ó.
    Double-click on some of the elements on the left. Then click Finish.

 

17. Try this a few more times and
    see if you can improve on the tree via your selection of sequence
    elements. Below is an example of a tree that looks a bit more similar to
    the expression-based tree, based on a subset of 8 sequence elements.

18. When youÕre happy with the
    results, click ÒNo (Save current subset and close)Ó, enter a name for the
    frequency table for your final selection, and save the image.

 

19. You can now have a look at some
    of the output files which will have appeared in the file you created in
    step 1. First have a look at the log file, which will be named according
    to the date and time you started the CoREG run. This will contain records
    of the incidence table and data file you loaded in at the start, the
    sequence elements that were identified, and the names of any files that
    were saved along the way. There will also be details of the MapMan files
    that were created. These will have names ending with
    Ò\_Clusters\_MapMan.txtÓ and Ò\_Genes\_MapMan.txtÓ. View these files in MapMan
    nowÉ

 

  

20. You will need
    to have the Mitochondria MapMan files that came with CoREG already loaded
    into MapMan. For details on how to do this, see the end of the ÒUsing
    CoREGÓ guide. To view the files that CoREG created, open MapMan and
    double-click on the Mitochondria pathway. Then load in the newly created
    files. Select Òadd experimentÓ from the File menu, click Òby nameÓ and
    type in a name for the new folder, e.g. ÒCoREG trialÓ. Then right-click on
    the new folder, and locate the file ending in Ò\_Clusters\_MapMan.txtÓ.
    Click on the new file from within MapMan, and you will see coloured boxes
    appear on the mitochondria pathways picture. Select ÒOptionsÓ from the
    Pathway menu, and change the scale to above 7, say 7.5. Change the Data
    size to L or XL so itÕs easier to see.

 

 

 

 

           

  

The boxes drawn on the pathway diagram
correspond to genes that were among those in the data file you loaded into
CoREG. Mouse-over a box to see precisely what that gene is.

 

The colour of the boxes indicates which of the
osmotic stress-related modules the gene was clustered into. The boxes coloured
dark blue (an AOX and an external NDH) denote genes that were in the most
highly up-regulated module. Those coloured medium blue correspond to genes that
were in the next highly up-regulated module (mainly TCA cycle genes in this
example), and those coloured pale blue were in the slightly up-regulated module
(e.g. 2 complex II genes, 2 defense genes). Similarly, dark red boxes indicate
genes belonging to the most highly down-regulated module (e.g. a complex IV
gene), bright red indicates the next down-regulated module (e.g. 2 TIM22
components) and pale red indicates the slightly down-regulated module (e.g. 3
protein synthesis genes).

 

 

 

 

ModuleFinder and CoREG
