# Supplementary material for: ModuleFinder and CoReg: alternative tools for linking gene expression modules with promoter sequences motifs to uncover gene regulation mechanisms in plants
Source: Plant Methods. 2006 Apr 11;2:8. doi: 10.1186/1746-4811-2-8 (PMC1479336; doi:10.1186/1746-4811-2-8)
Supplement: Additional File 6 — User guide (htm files).zip Instruction for use in htm format [file 1746-4811-2-8-S6.zip › User guide(htm files)/Index.htm]

ModuleFinder and CoREG


# ModuleFinder and CoREG

 

Complementary R-based programs for the identification of
co-ordinated gene expression modules and promoter elements involved in the
co-regulation of co-expressed genes.

 

## General Installation

### ModuleFinder

## ¥        About ModuleFinder

## ¥        ModuleFinder Tutorial [PDF]

***¥        Using ModuleFinder***

### CoREG

## ¥        About CoREG

## ¥        CoREG Tutorial [PDF]

***¥        Using CoREG***

***¥        CoREG and MapMan***

 

#### Disclaimer

These programs were created for the analysis of small
subsets of genomic expression data from microarray experiments in Arabidopsis,
as part of an Honours project in the Plant
Molecular Biology Group in the Department
of Biochemistry and Molecular Biology at the University of Western Australia.

 

Although the principles and methods employed in this
analysis are applicable to other data sets, the programs have not been tested
with data sets involving more than 1000 genes or more than 300 microarray
experiments, or data from other organisms. Currently, CoREG is not designed for
use with genes other than those from Arabidopsis, which should be labelled with
their Agi locus identifier (ATxGxxxxx). Anyone interested in using CoREG with
other genomes can make the necessary modifications to the code themselves, or
request help from me.

 

Any queries, email Kathryn Holt at katholt@graduate.uwa.edu.au.
