# Supplementary material for: ModuleFinder and CoReg: alternative tools for linking gene expression modules with promoter sequences motifs to uncover gene regulation mechanisms in plants
Source: Plant Methods. 2006 Apr 11;2:8. doi: 10.1186/1746-4811-2-8 (PMC1479336; doi:10.1186/1746-4811-2-8)
Supplement: Additional File 6 — User guide (htm files).zip Instruction for use in htm format [file 1746-4811-2-8-S6.zip › User guide(htm files)/Install.htm]

To run ModuleFinder and CoREG you will need:


# Installation

 

Before you
can run ModuleFinder and CoREG
you will need to do the following:

 

1)      Install a copy of the statistical
package R. You can download the latest version from http://cran.r-project.org/mirrors.html,where
youÕll also find installation instructions and additional help with R.

2)      Install a number of R packages. You
can download packages from CRAN and Bioconductor from within R, via the
Packages menu.

a.       To use the Windows-based graphical
interface for either program you will need the package *tkWidgets* fromBioconductor.

b.      To use ModuleFinder you will need
the packages *gregmisc* from CRAN, and *ctc* fromBioconductor.

c.       To use CoREG you will also need the
following packages from CRAN:

*i.**cluster*

*ii.**combinat*

*iii.**e1071*

*iv.**gregmisc*

*v.**maptree*

                                                          
vi.     
*rpart*

 

# Using the programs

Each time
you want to use the programs, you will need to first open R, then select
ÔSource R codeÉÕ from the ÔFileÕ menu, and locate the file ÒModuleFinder.RÓ
and/or ÒCoREG.RÓ (Windows) or ÒModuleFinderMAC.RÓ and/or ÒCoREGMAC.RÓ (Mac).

You will
then be asked to locate your datafile(s) and set some parameters.

For details, read the tutorials and user guides for ModuleFinder
or CoREG.

 

 

ModuleFinder and
CoREG
