# Supplementary material for: ModuleFinder and CoReg: alternative tools for linking gene expression modules with promoter sequences motifs to uncover gene regulation mechanisms in plants
Source: Plant Methods. 2006 Apr 11;2:8. doi: 10.1186/1746-4811-2-8 (PMC1479336; doi:10.1186/1746-4811-2-8)
Supplement: Additional File 6 — User guide (htm files).zip Instruction for use in htm format [file 1746-4811-2-8-S6.zip › User guide(htm files)/MFTut.htm]

ModuleFinder Tutorial


# ModuleFinder Tutorial

PDF version

 

This
tutorial will take you step-by-step through a run of ModuleFinder on a Windows
PC, using a set of data from stress-related experiments from AtGenExpress. You will
need to locate the two data files that came with ModuleFinder Ð
ÒAtGenExpress\_DataLogR.txtÓ andÒAtGenExpress\_Pvals.txtÓ.

 

You should
already have installed R and the necessary packages as outlined in the
installation guide.

 

1. Create a new folder, call it
   something like ÔModuleFinder TutorialÕ and copy the two data files into
   it.
2. Open R.
3. Set the current directory in R
   to the one you just created in step 1.

 

4. Load in the ModuleFinder R
   code, by going up to the file menu and selecting ÒSource R CodeÉÓ

5. R will now load in the packages
   it requires. If you don't have necessary packages installed, do this now.  
   (See the installation guide for details.)

 

 

6. A window will pop up, asking
   you to locate the data file you want to load.

If you followed Step 1 and set the current
directory correctly, you should only see the two files (data and p-values).
Double-click on the data file, then click Finish:

 

 

7. A second window will pop up,
   this time asking you to locate the file of p-values. Double-click the
   PVals file and click Finish.

 

8. Another window will pop up to
   say you need to indicate which columns contain gene information rather
   than data. Click OK.

In the window that comes up, on the left you
will see a list of the names of all the columns in the data file. The first 5
contain information about the genes, the rest contain average log ratios of the
experimental vs control conditions from lots of different experiments. Select
the first 5 column names, and move them over to the right hand box by clicking
on ÒSelect >>Ó. Then click Finish:

 

 

9. You will now be asked to select
   a combination of these gene information columns which together will
   provide an informative, unique label for each gene. Since the data in this
   file is from Affymetrix GeneChips, on which single genes can be
   represented by more than one array element, the Locus alone will not be a
   unique label. So select Locus, then ArrayElement, and then Name, to create
   our unique label. (ItÕs handy to include Name here, so you can more easily
   recognize genes of interest in the output files.)

 

10. Next you will be asked to select a
    starting set of experiments. You can try different combinations of
    experiments later, but for this example scroll down and double-click on
    ÒOsmotic\_Shoot\_24hÓ and then ÒSalt\_Shoot\_24hÓ. Click Finish.

 

 

11. Now you will be given the
    opportunity to change some parameters.

Change the ÒName for output filesÓ to something
like ÒTutorialÓ. The names of any files created by ModuleFinder will begin with
this title.

Change the maximum p-value to 0.1. Leave the
remaining parameters as they are.

12. Now ModuleFinder will begin to
    do its work! Firstly, it will identify the genes with p-vals<0.1 in
    both the experiments we selected. On the R console, it will print out the
    number of genes that passed this criteria (in this case 133), and then
    list these genes (using the unique labels we specified at step 9):

 

number
of genes: 133

Selected
genes:

 AT2G39795 245063\_at ND

 AT2G39725 245064\_at ND

 AT2G40890 245101\_at CYP98A3

 AT2G47490 245152\_at ND

 AT2G33210 245164\_at HSP60-2

...
... ... ... ... ......

 

13. These genes will then be
    clustered according to their expression in the two experiments. The number
    of clusters is printed to the console. This combination of genes and
    experiments is the beginning of the module identification.

 

Then, ModuleFinder will begin to search for
other experiments in which these gene modules behave as modules, that is
experiments in which the expression of genes in the same module are similar,
but genes in different modules are expressed differently. Experiments are added
one-by-one, and the progress is reported on the console:

 

Treatments:  Osmotic\_Shoot\_24h  and 
Salt\_Shoot\_24h

number
of clusters: 12

Adding
variable'Osmotic\_Shoot\_12h'. Between-module/Within-module variance: 14.81115

Adding
variable'Salt\_Shoot\_12h'. Between-module/Within-module variance: 7.564039

Adding
variable 'ColdShoot1h'.Between-module/Within-module variance: 6.71945

Adding
variable'Osmotic\_Shoot\_6h'. Between-module/Within-module variance: 6.402471

Adding
variable'ABA\_1h'. Between-module/Within-module variance: 5.320692

 

14. When ModuleFinder canÕt find any
    more experiments to add to the modules, it will report this and end the
    run:

 

No
more treatments to add

null
device

         
1

> 

 

            There
may be warning messages reported at this point too. Ignore them.

The red pointer indicates that R has finished
what it was doing and is ready to go again.

 

  

15. You are now ready to look at the
    ModuleFinder output. A number of files will have appeared in the directory
    you created at step 1. Open the PDF file first. The first page reports
    what the starting parameters were:

 

**MODULEFINDER
RESULTS**

**Analysis
name: Tutorial**

**Starting
treatments:Osmotic\_Shoot\_24h and Salt\_Shoot\_24h**

**Minimum
between/within cluster variance ratio for adding treatments: 4**

**Clustering
parameters: euclidean distance, ward linkage**

## P−value cut−off for genes: 0.1

 

The second page will show a clustering tree and
heatmap for the genes that were differentially expressed (p<0.1 in the
p-values file) in both of the starting experiments. The next few pages will
contain pie charts displaying the functional breakdown of each of the resulting
modules, with the corresponding heatmaps printed below.

After this, you will see a new heatmap, which
now includes the first additional experiment to be added into the modules,
followed by the new clustering tree that results from reclustering the genes
using data from the three experiments now in the module, followed by new pie
charts for the resulting modules. The same output is printed for each added
experiment. Finally, the last page contains a heatmap displaying the expression
of the module genes in the experiments that didnÕt end up in the module.

 

16. Go to page 80, where the
    experiment ÒABA\_3hÓ was the last to be added. WeÕll take this subset of
    genes and experiments as the final result.  (If you have a look at the next few
    experiments that were added, youÕll see that the changes in expression
    levels were very very low, so weÕll ignore these.)

 

17. Now go back to the directory
    containing the ModuleFinder output. This contains cluster files (for
    viewing in TreeView and similar programs, extensions .cdt and .gtr) for
    each stage of the ModuleFinder run. Start TreeView (http://jtreeview.sourceforge.net)
    and use it to open the file ending in ÒAddABA\_3h.cdtÓ.

Select ÒPixel settingsÉÓ from the Settings menu.
For consistency with ModuleFinder, CoREG and MapMan output itÕs best to change
the default colour settings so that blue is positive and red is negative. You
will also need to adjust the contrast in order to resolve the expression
measures (since the data was logged the values are quite small). Set the
contrast to 1 and close the settings window. You can now explore the genes,
clustering tree and experiments in TreeView, and save any heatmap/clustering
images or gene lists for all or part of the tree.

 

18. ModuleFinder also created files
    containing the expression data for the gene and experiment subsets at
    every stage. The file ending with ÒGeneClusters\_AddABA\_3h.csvÓ contains
    the data for our final modules, and you can now use this file to explore
    promoter sequence elements in CoREG.

ModuleFinder and
CoREG
