# Supplementary material for: ModuleFinder and CoReg: alternative tools for linking gene expression modules with promoter sequences motifs to uncover gene regulation mechanisms in plants
Source: Plant Methods. 2006 Apr 11;2:8. doi: 10.1186/1746-4811-2-8 (PMC1479336; doi:10.1186/1746-4811-2-8)
Supplement: Additional File 6 — User guide (htm files).zip Instruction for use in htm format [file 1746-4811-2-8-S6.zip › User guide(htm files)/UseCR.htm]

Using CoREG


# Using CoREG

 

## This guide explains how to use and interpret CoREG.

For details
on getting started, read the Installation Guide
first.

For a
general overview of how CoREG works and what itÕs for, see the CoREG Overview.

 

## Preparation

***Running CoREG***

***CoREG Output***

***CoREG and MapMan***

 

## Preparation

1. Make sure you have the
   necessary files ready.

You will need to have at least two files to run
ModuleFinder, which should all be in ***comma-separated format*** ***(.csv)***
(Excel can save files in this format):

 

1. Incidence data. ***Either***  (1) a set of promoter sequences
   and a list of potential sequence elements to search for, ***or***
   (2) an incidence table. The first time you use CoREG for a particular set
   of genes, you will need the promoter sequence for each gene and a list of
   sequence elements, from which an incidence table will be created. This
   table records the presence or absence of each of the sequence elements in
   each of the promoters, and will be automatically saved for future use.
   The next time you use CoREG with the same set of genes, you can simply
   load this incidence file directly.

1.                                         
***Promoter
sequences &Sequence elements***

a.                                          
The
promoter sequences should be in a single file in FASTA format. NOTE: For
Arabidopsis genes, these sequences can easily be obtained from TAIRÕs bulk
sequence download facility (http://www.arabidopsis.org/tools/bulk/sequences/index.jsp).Simply
paste in a list of locus identifiers, select ÒUpstream Sequences Ð1000bpÓ as
the dataset and FASTA as the output format. Then save the results as a text
file Ð this will be your sequence file, and can be loaded directly into CoREG.

b.                                         
You
can either provide a list of sequence elements, or use the built-in set of all
hexamers (the 4096 possible6-bp sequences). If you want to provide your own
list, it should be in the form of a comma-separated table, with the first
column providing the nucleotide sequences themselves, and the second providing
a name for each one. Note that the sequences must only consist of the base
pairs (A/C/G/T) in uppercase; wildcards (N/R/Y/Y/etc) wonÕt work. Also note
that the more sequences you have, the longer it will take to create the
incidence table (which is quite computer-intensive), and the longer it will
take to run CoREG.

2.                                         
***Incidence
table***. Whenever
you load in promoter sequences and a list of sequence elements to create a new
incidence table, this table will be saved to the current directory as a
comma-separated file called ÒCoREG\_IncidenceTable.csvÓ. For subsequent runs of
CoREG you can just load in this table directly. This is always a good idea since
it can take quite a while to calculate a new incidence table every time.

 

2. File 2: ***Expression data***
   for a subset of genes and experiments of interest.

 

1.                                         
This
should have genes in rows, the first column with locus identifiers and
experimental data in the rest of the columns. The locus identifiers in the
first column are used to look up information in the incidence table, so should
be precisely the same as the locus identifiers of the initial promoter
sequences. This is case sensitive. If you follow the advice above about
retrieving promoter sequences from TAIR, and make sure you label the expression
data using the same locus identifiers (in uppercase), you should have no
trouble.

 

2.                                         
This
file ***should not contain all your expression data***. This should be
only the data for a subset of genes and experiments. Best results have been obtained
with data on around 60 genes, in 10 experiments. The idea is that you use
methods such as ModuleFinder to identify genes that respond in co-ordinated
ways to a subset of experiments, and extract only the data for these genes and
experiments. The output data files that ModuleFinder creates (ÒRunX\_GeneClusters\_AddY.csvÓ)
are designed to be loaded directly into CoREG, but you could source these data
subsets via any method you choose.

 

2. Set the directory in which to
   save ModuleFinder output.

1. This is done within R, by
   choosing ÒChange dirÉÓ from the File menu.

(Click for
screenshot)

2. CoREG can create a lot of
   output files, so it is a good idea to create a new directory for this
   purpose. As with ModuleFinder, the best way to organize your use of CoREG
   is to create a new folder, and copy your promoter sequences, sequence
   elements, incidence tables, and expression data subsets into it. Then,
   set it as the current R directory. If you are using the Mac (non GUI)
   version you ***have to*** set the current directory to the one
   containing the input data files.

 

 

  

## Running CoREG

 

1.   To
run CoREG with your data, in R select ÔSource R codeÉÕ from the ÔFileÕ menu,
and locate the file ÒCoREG.RÓ (GUI version for Windows) or ÒCoREGMAC.RÓ (any
other platform).This will load all the CoREG functions into R, ready for using.(Click for screenshot)

 

You will then be asked for a series of inputs,
including the location of your data files and some additional parameters.

a.      
In
the GUI version, this will be via dialogue boxes which allow you to make
selections or enter information.

b.     
In
the non-GUI version this will be via the command line, where questions will be
printed, along with some guidance about how to answer them. To respond to these
questions you will generally need to enter either a number, a name or a vector
of these, then press RETURN.

In R, all text must be surrounded by quotes to
be recognized as such. (e.g. ÒtextÓ or Òdatafile.txtÓ).Numbers
can be entered as-is (i.e. 5 not Ò5Ó). Vectors in
R are surrounded by brackets and preceded by a lowercase ÔcÕ, e.g. c(1,2,3) or c(ÒExp1Ó,ÒExp2Ó).

 

2.   You
will first be asked whether you have an incidence table ready to load, or
whether you need to create a new one from promoter sequences and a list of
sequence elements. You will then be asked to locate the appropriate files.

a.      
In
the GUI version you do this via a file browser. (Click for screenshot)

b.     
In
the non-GUI version you will need to type y or n (yes or no) in answer to the question, then the names of the relevant
files, surrounded by quotes.

 

3. Next, you will be asked to
   locate the expression data file you wish to use.

(Click
for screenshot)

 

4. Then, CoREG will cluster the
   genes in the data file according to the expression data, and draw a
   heatmap and clustering tree in the R graphics window. (If you are using
   ModuleFinder output for your data files, this tree should look just like
   the ones in the corresponding ModuleFinder output.)

1. Now, you need to click on
   branches of the tree, to indicate how you want the tree broken down to
   form gene clusters. The clusters must be true, meaning they must be able
   to be formed by truncating the entire tree at some height, and therefore
   each gene must be in a cluster. The rest of the CoREG run will be aimed
   at identifying sequence elements that can discriminate between these
   clusters; for more information on what you are doing at this step, see
   the CoREG overview.
2. When you click on the
   branches, red boxes are drawn around the resulting clusters. When the
   tree is appropriately broken down (remember every gene must be in a
   cluster, you canÕt leave bits of the tree out), right-click in the image
   window and select ÒStopÓ. (On a Mac, option-click instead of
   right-click.) If there was a problem with the way you broke down the tree,
   it will be redrawn and you can start again. Note that if you make an
   error, just right-click to stop and start again on the newly drawn tree.

(Click
for screenshot)

 

5. When youÕve broken down the
   tree properly, you will be asked if you want to save a MapMan file of the
   clusters. If you want to create the file, say yes and enter a file name.
   In this file, each gene will be labeled with a number to indicate which
   cluster it belongs in. So, when you load the file into MapMan, the genes
   will be coloured according to cluster membership. YouÕll probably need to
   increase the scaling in MapMan to see the different colours (do this via
   the options menu). CoREG will also save a second file for viewing in
   MapMan, in which genes are labeled with their average expression across
   the set of experiments. So, when you load this file in MapMan, genes will
   be coloured according to their average expression level. You may need to
   change the scaling again to see the differential expression, depending on
   the expression levels in your particular dataset. For more details and
   information on what MapMan is and how to get it, see the CoREG & MapMan guide below.

 

6. Next you will be asked to set
   some parameters. You must set the minimum number of sequence elements to
   be found at each split of the tree. CoREG will return at least this many
   sequence elements for each split; if there are multiple elements at the
   same level, then more might be returned. Then, if you like you can set a
   maximum frequency tolerance, between 0 and 0.5. For example if you enter
   0.3, CoREG will find all sequence elements that are present in at least
   70% of one side of the split and no more than 30% of the other side.

(Click for
screenshot)

 

7. The results of the search for
   sequence elements will be printed on the console as the search progresses.
   When the search is finished, if sequence elements were found, the results
   will be displayed graphically in the R image window. At this point you
   will be asked if you want to change any parameters Ð note this includes
   reselecting the clusters from the tree. If you arenÕt happy with the
   sequence elements that were found, answer yes and youÕll be taken through
   steps 4 to 6 again.

 

NOTES ON THEIMAGES (Click for screenshot):

CoREG will have drawn four images in the image
window. The one titled ÔOriginal TreeÕ displays the earlier clustering tree
truncated to form gene expression clusters according to your earlier
selections. The branches indicate the resulting set of clusters, labeled with a
number. The clusters will be labeled with the same numbers in all subsequent
output.

Next there is a frequency map for the
discriminating elements that CoREG found. The numbers to the left indicate the
gene expression clusters, and the labels on the bottom indicate sequence
elements. The blocks are coloured to represent the frequencies of the sequence
elements in the promoters of the cluster genes. White indicates a frequency of
0 (i.e. none of the genes in that expression cluster have that element in their
promoters), black indicates a 100%frequency, and shades of grey indicate
intermediate frequencies.

Below this is the frequency map for the current
selection of sequence elements. At this point this will be the same as the
first frequency map.

To the right of this is a hierarchical
clustering tree displaying how the gene expression clusters are related
according to the frequencies of the sequence elements. This can be thought of
as the promoter-based tree. The idea is to try to get this tree to match the
expression-based tree above it as closely as possible, by identifying the
ÔrightÕ subset of sequence elements.

 

NOTE ON SAVINGIMAGES (Click for screenshot):

The graphical output of CoREG will not be saved
automatically. If at any time you want to save an image (i.e. whatever is
displayed in the R image window), simply click on the image window and choose
ÒSave asÉÓ from the File menu. You can save R images as PDF files (which can
then be edited in Adobe Illustrator) or as jpeg, bitmap, png, etc. ItÕs a good
idea to save the images of the results that you like, for later consideration.

 

8. When you are happy with the set
   of sequence elements that were found, CoREG will save the frequencies of
   the sequence elements in the chosen clusters, and ask for a name for this
   file. The file will be in comma-separated format, so you can open it in
   Excel later if you wish.

 

9. You will then be asked if you
   want to try different subsets of the sequence elements. The aim here is to
   find a subset of these elements that will result in a promoter-based tree
   (below) with a similar structure to the expression-based tree (above). You
   can try random subsets if you like, or you can select elements yourself.
   This step can be repeated as many times as you like, and you will always
   be able to save the frequencies of the current subset if you wish. And
   donÕt forget that you can save an image for the results you like at any
   time (File/Save asÉ).

When the run has completed, youÕll be asked if you want to run CoREG again with
the same incidence table. If you answer yes, the incidence table will be
automatically loaded, and you will simply have to select a new expression data
file and change any parameters as you wish.

 

 

## CoREG Output Files

 

1.   ***Log
file***. CoREG creates a log file to record the progress of each CoREG run.
These are text files, named with the date and time of the run. The file records:

1. the incidence table, promoters
   and/or sequence elements that were used
2. the input data file
3. details of any MapMan output,
   including the titles of the files and a record of how clusters were
   labeled
4. the parameter settings
5. results of the search for
   sequence elements
6. which sequence elements were
   used at each stage of clustering
7. names of any files that are
   saved during the run

2.   ***Frequency
tables***. CoREG saves the frequency tables for every sequence element
search, the final set of sequence elements, and any subsets you choose to save
along the way. These are comma-separated files, named with whatever names you
supplied during the run plus the Ò.CSVÓ extension.

3.   ***MapMan
files (optional)***. CoREG can save files that contain records of the
clusters that you select, which can be loaded into MapMan. See the guide below
for details.

 

  

## CoREG and MapMan

MapMan is a
Java program that allows you to annotate images with data from a text file
(Download from http://gabi.rzpd.de/projects/MapMan/).Images
can be loaded into the program that represent pathways of genes, then mapping
and files are loaded which assign gene identifiers into functional categories.
Then pathway images can be annotated, which involves mapping gene identifiers
to particular positions on the image. Then you can load in a file containing a
list of gene identifiers with values assigned to each (e.g. an expression value
from a particular experiment). The genes in the file then appear as dots in the
appropriate positions on the pathway image, coloured according to the value
(e.g. expression level) in the loaded file. This allows you to more easily
identify if a number of genes in a pathway were induced or repressed in an
experiment. For example, the mapping below shows 6 clusters of Arabidopsis
mitochondrial genes mapped to an image depicting mitochondrial functions. The
genes coloured blue were in clusters that were up-regulated in response to a
set of abiotic treatments; those in red belong to down-regulated clusters. The
darker the colour, the greater the average fold change in expression of the
cluster. Thus it is easy to spot that thereÕs a cluster of up-regulated TCA
cycle genes (medium blue), a cluster of down-regulated protein fate genes
(medium red Ðcomponents of import machinery, heat shock proteins, protein
synthesis), and a cluster of highly up-regulated genes including an alternative
oxidase and an external class alternative NADH dehydrogenase (dark blue), which
together encode an a bypass of the mitochondrial respiratory chain.

A number of
mappings and annotated images come with the standard MapMan download, but you
can add your own if you like. The one shown above is included in the
ModuleFinder/CoREG zip file. To load it for use with your own data, you need
to:

1.                                                           
Open
MapMan.

2.                                                           
Choose
Òadd mappingÓ from the file menu, click Òfrom fileÓ, and locate the file
ÒMitochondriaMapping.xlsÓ.

3.                                                           
Choose
Òadd pathwayÓ from the file menu, click Òfrom fileÓ, and locate the file
ÒMitochondria.bmpÓ.

4.                                                           
A
dialogue box should appear, asking you to choose a mapping for this pathway.
Select ÒMitochondriaMappingÓ and click OK.

5.                                                           
You
should now be able to load in data including Arabidopsis genes targeted to the
mitochondria, and visualize them on these pathways. See MapMan help for
details. Note that the gene identifiers in the mapping file are Agi loci with
uppercase letters, so you will need to use the same identifiers in the data
files you want to view.

 

 

**ModuleFinder & CoREG**
