# Supplementary material for: ModuleFinder and CoReg: alternative tools for linking gene expression modules with promoter sequences motifs to uncover gene regulation mechanisms in plants
Source: Plant Methods. 2006 Apr 11;2:8. doi: 10.1186/1746-4811-2-8 (PMC1479336; doi:10.1186/1746-4811-2-8)
Supplement: Additional File 6 — User guide (htm files).zip Instruction for use in htm format [file 1746-4811-2-8-S6.zip › User guide(htm files)/UseMF.htm]

Using ModuleFinder


# Using ModuleFinder

 

## This guide explains how to use and interpret ModuleFinder.

For a
step-by-step example, see the Tutorial first.

You should
read the Installation Guide first.

 

## Preparation

***Running ModuleFinder***

***ModuleFinder Output***

 

## Preparation

1. Make sure you have your data
   ready.

You will need to have two files to run
ModuleFinder, which should both be in ***tab-delimited format*** (Excel
can save files in this format):

1. File 1: Expression data from a
   set of experiments.

1.                                         
Each
row in this file should correspond to a gene.

2.                                         
The
first few columns should contain gene information, for example the gene name,
array element, locus (Agi identifier), gene description and functional
category.

a.                                          
If you
want to use your ModuleFinder results with CoREG, one
of these columns must contain the *Arabidopsis* Agi locus identifier. This
column should be labeled ÒLocusÓ.

b.                                         
One or
a combination of these columns must result in a unique identifier for each
gene. Note that a single gene locus may be represented by multiple elements on
an array. Thus a locus identifier alone may not provide a unique identifier for
every data point. However, the combination of locus and array element will
provide an informative, unique identifier for every element in your data set,
so it is a good idea to have at least these two columns in your data file.

c.                                          
When
ModuleFinder creates clustering files for viewing TreeView, it will label genes
using the columns named ÒLocusÓ and ÒDescriptionÓ, so if you want to use these
files youÕll need to make sure you name your columns appropriately.

d.                                         
ModuleFinder
will attempt to provide pie charts displaying the functional breakdown of the
modules it identifies. However it can only do this if you provide a column in
your data file indicating a functional category for each gene.

3.                                         
The
remaining columns should contain summarized experimental data from a number of
array experiments. It will work best if this is the ***average log ratio***
of gene expression under two or more replicates of experimental vs control
conditions.

 

An example file:

|  |
| --- |
|  |
|  |  |

2. File 2: P-values corresponding
   to the expression data.

1.                                         
This
should have the exact same layout as the expression data file, with the genes
in rows, and gene information and experimental data in columns. ***All rows
and columns should be in the same order as the expression data file.***

2.                                         
The
experimental data columns should contain p-values from a statistical test of
differential gene expression between the replicated experimental and control
samples.

 

2. Set the directory in which to
   save ModuleFinder output.

1. This is done within R, by
   choosing ÒChange dirÉÓ from the File menu.

(Click for screenshot)

2. ModuleFinder can create a lot
   of output files, so it is a good idea to create a new directory for this
   purpose. Perhaps the best way to organize your use of ModuleFinder is to
   create a new folder, and copy your expression data and p-value files into
   it. Then, set it as the current R directory. If you are using the Mac
   version you ***have to*** set the current directory to the one
   containing the data files.

 

 

## Running ModuleFinder

1.   To
run ModuleFinder with your data, in R select ÔSource R codeÉÕ from the ÔFileÕ
menu, and locate the file ÒModuleFinder.RÓ (GUI version for Windows) or
ÒModuleFinderMAC.RÓ (for running on Mac). This will load all the ModuleFinder
functions into R, ready for using. (Click for
screenshot)

2.   You
will then be asked for a series of inputs, including the location of your data
files and some additional parameters.

a.       In the Windows version, this will be
via dialogue boxes which allow you to make selections or enter information.

b.      In the Mac version this will be
partly via dialogue boxes and partly via the command line, where questions will
be printed, along with some guidance about how to answer them. To respond to
these questions you will generally need to enter either a number, a name or a
vector of these, then press RETURN.

In R, all text must be surrounded by quotes to
be recognized as such. (e.g. ÒtextÓ or Òdatafile.txtÓ).Numbers
can be entered as-is (i.e. 5 not Ò5Ó). Vectors in
R are surrounded by brackets and preceded by a lowercase ÔcÕ, e.g. c(1,2,3) or c(ÒExp1Ó,ÒExp2Ó).

3.   You
will first be asked to locate your expression data file, followed by your
p-value file. (Click for screenshot)

4.   Next
you will be asked to indicate which columns contain gene information rather
than data. For example, in the data file set out above, the file begins with 5 columns
containing gene information Ð Array Element, Locus, Name, Description and
Function. (Click for screenshot)

5. Next you will be asked to
   indicate a combination of these columns which results in a unique
   identifier for each gene. This is also how the genes will be labeled in
   the output files, so choose a combination that will uniquely and
   informatively label each data point. A good choice is the locus, array
   element and name or description, as the locus and name/description will
   help you to recognize interesting genes, while the array element will
   ensure labels are unique.

1. In the Windows version, you
   are presented with a list of the columns containing gene info, and asked
   to select a combination of these. Note that that the order you select
   them in is the order they will appear in gene labels. (Click for screenshot)
2. In the Mac version you will
   need to type a vector indicating the columns that you want to use. In R,
   vectors are surrounded by brackets and preceded by a lowercase c, like
   this:

                        So
if your gene info columns are like this:

                                    Column1
= Array Element

                                    Column2
= Locus

                                    Column3
= Name

                        then
entering:

                                    c(2,1,3)

will ensure data points are labeled by locus
identifier, array element and gene name (in that order).

6. Next you will be asked to
   select an initial subset of experiments to run ModuleFinder with. To do an
   unsupervised run, leave this blank. (See the ModuleFinder
   Overview for more information about this step.)

(Click for screenshot)

7. Finally, you will be asked to
   set a number of parameters.

In the Windows version, this will be via a
single dialogue box in which you can leave the default values or enter your
own. (Click for screenshot)

In the Mac version you will be asked about each
parameter in turn via the command line. There is also some guidance on the
command line about responding to these prompts.

1. *Name for output files*. The ModuleFinder output
   files will begin with whatever you enter here. It is helpful for keeping
   track of which files belong to which run of ModuleFinder.
2. *Minimum correlation between
   treatments*.
   Relevant only to unsupervised runs. ModuleFinder will be run on every
   pair of treatments whose raw correlation, across all elements in the data
   set, is above this cut-off. Depending on how similar your various experiments
   are, you may want to raise or lower the default value.
3. *Minimum between:within
   variance ratio*.
   ModuleFinder chooses which new experiments to add into a module by
   comparing how co-ordinated the expression of the current gene clusters
   are in all experiments not already in the modules. The ratio of
   between-cluster variance to within-cluster variance is used to assess
   this. There is a bias here towards the selection of experiments in which
   not only are genes within clusters behaving in a co-ordinated way, but in
   a way that is different from the behaviour of the other clusters.
   Therefore the actual value of this ratio will creep ever lower as the
   number of genes and number of clusters increases. It is a good idea to
   start with the default value, check the results, then tweak it if you
   think it necessary.
4. *Maximum p-value*. Only the genes whose
   p-values are below this cut-off in the initial experiment set will be
   included in the subsequent modules. If you find the default value leads
   to too many or too few genes being included, try changing this value.
5. *Number of clusters*. By default, ModuleFinder
   will split the genes into the number of clusters closest to the square
   root of the number of included genes. This is an attempt to balance the
   number and size of the gene clusters that are produced. However, if you
   find that it is splitting up some decent looking clusters, or lumping
   together some groups that are internally variable, you can specify here
   the number of clusters that you want to break the clustering tree into.
6. *Recluster after each treatment
   is added*? The
   default is to re-apply the clustering algorithm after each new experiment
   is added to the modules. This allows the modules to evolve a little as
   the algorithm progresses. However to stop this, and only include
   experiments that match the clustering of the initial subset, change this
   to FALSE.
7. *Distance & linkage methods*. These are the methods that
   will be used to cluster genes. ModuleFinder uses RÕs hclust and dist functions for hierarchical
   clustering function. The available distance methods are ÒeuclideanÓ,
   ÒmaximumÓ, ÒmanhattanÓ, ÒcanberraÓ,
   ÒbinaryÓ or ÒminkowskiÓ. The available linkage methods are ÒÓ. For more
   information about these, see the R help for the dist and hclust functions (type help(dist) or help(hclust)in R).
8. *Height & width of PDF file*. The main output of
   ModuleFinder is a PDF file including the parameter settings, heatmaps for
   each stage of clustering and, if you provide a column containing
   functional categories, pie charts representing the functional breakdown
   of each module. The default page size for this file is A4, but you can
   specify its height and width (in inches) if you want to make it bigger.
   This may be useful if you find you are getting modules with more genes
   than can be comfortably displayed on an A4 page.

When
the run has completed, youÕll be asked if you want to run ModuleFinder again
with the same data set. If you answer yes, the same expression data and
p-values will be automatically loaded, and you will simply have to select a new
starting set of experiments and change any parameters as you wish.

 

## ModuleFinder Output

1.   PDF
file.

The
major output file is a PDF file. The first page states the parameter settings.
The subsequent pages provide heat maps with clustering trees for each stage of
the process. If you specified functional categories in your data file, there
will also be pie charts displaying the functional breakdown of each module into
these categories. The last page contains a heat map displaying the expression
of module genes in the experiments not included in the module.

2.   Data
files.

Expression
data for the current subset of genes and experiments is saved at each stage as
a comma-separated file (you can open these in Excel). The files are named
according to the title that you provided at the start of the run, followed by ÒGeneClusters\_Initial.csvÓ
for the initial subset, and ÒGeneClusters\_AddXXXX.csvÓ for each added
experiment XXXX.

These
files can also be imported straight into CoREG for
promoter analysis

3.   Cluster
files (.cdt,.gtr) for viewing in TreeView and similar programs.

Cluster
files are also saved at each stage. These files can be viewed directly from
within TreeView and similar programs (TreeView can be downloaded from http://rana.lbl.gov/EisenSoftware.htm;
Java TreeView, which I find particularly stable and flexible, can be downloaded
from http://jtreeview.sourceforge.net).The
files are named the same as the Data files, but with the extensions .cdt and
.gtr.

 

 

 

**ModuleFinder&
CoREG**
